# Quadruple Bonding of Alkaline Earth Atoms in AeCLi_4_ (Ae = Be − Ba) Complexes

**DOI:** 10.1002/jcc.70449

**Published:** 2026-07-03

**Authors:** Yahui Li, Chengxiang Ding, Sudip Pan, Gernot Frenking

**Affiliations:** ^1^ Institute of Atomic and Molecular Physics Jilin University Changchun China; ^2^ Centre for Interdisciplinary Research SRM University‐AP Amaravati Andhra Pradesh India; ^3^ Philipps‐Universität Marburg Marburg Germany

## Abstract

The results of quantum chemical calculations of the complexes AeCLi_4_ (Ae = Be − Ba) are reported at the BP86‐D3(BJ)/def2‐QZVPP and CCSD(T)/def2‐QZVPP level. The calculated equilibrium geometries with Ae = Be, Mg have a trigonal bipyramidal geometry (*C*_*3v*_ symmetry) as the energetically lowest‐lying form. A slightly higher‐lying isomer has a square pyramidal geometry (*C*_4*v*_ symmetry), which is only < 1 kcal/mol less stable than the *C*_3*v*_ form. In contrast, only the square pyramidal structure is an energy minimum of the heavier homologues with Ae = Ca, Sr, Ba. The calculated bond dissociation energies of the Ae‐CLi_4_ bond are very high. The strongest bond is computed for the Be‐CLi_4_ bond (*D*_e_ = 82.9 kcal/mol at CCSD(T)/def2‐QZVPP). The weakest bond is calculated for the Mg‐CLi_4_ bond (*D*_e_ = 40.6 kcal/mol). The heavier homologues have values between *D*_e_ = 63.1 kcal/mol (Sr‐CLi_4_) and *D*_e_ = 72.0 kcal/mol (Ba‐CLi_4_). Inspection of the occupied valence orbitals and the AdNDP results suggests that there are four Ae‐CLi_4_ bonds in the complexes. This is supported by the EDA‐NOCV analysis, which reveals that there is a dominant Ae → CLi_4_ σ‐donation, which is enhanced by weaker Ae ← CLi_4_ σ‐backdonation and degenerate Ae ← CLi_4_ π‐backdonation. The best signature of the chemical bonds is Ae 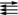 CLi_4_. The lighter atoms, Be, Mg, use their (n)s and (n)p AOs for the covalent bonds, whereas the heavier atoms, Ca, Sr. Ba, employ their (n)s and (n‐1)d AOs for the covalent interactions. The NBO method does not provide a reasonable account of the covalent bonds, because it does not consider the (n)p and (n‐1)d AOs of Ae atoms as genuine valence orbitals.

## Graphical abstract

Quantum chemical calculations of the complexes AeCLi_4_ (Ae = Be—Ba) show that the molecules have strong Ae‐Cli_4_ bonds, which consist of four dative bonds. The best signature of the chemical bonds is Ae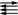 CLi_4_.

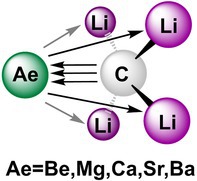

## 1 Introduction

**FIGURE 1:**
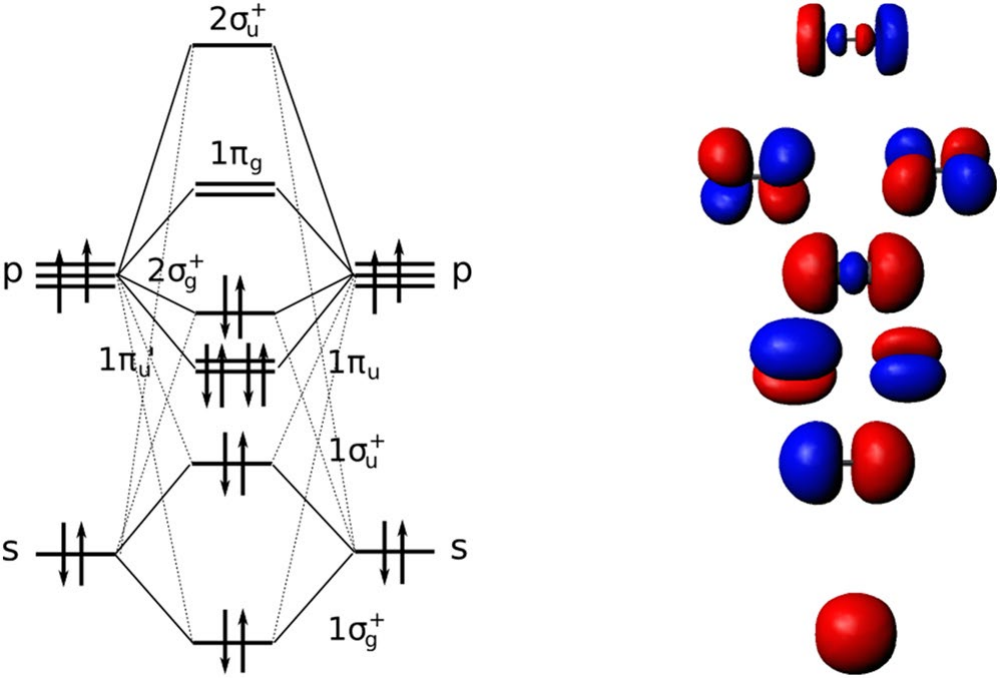
MO correlation diagram between two main‐group atoms with electron occupation of N_2_ and the associated orbitals.

The bond order, which is indicated by the number of bonding electron pairs between two atoms, is one of the fundamental concepts in chemistry. The aufbau principle for chemical bonding between two main‐group atoms, which use their valence s and p orbitals for the formation of chemical bonds following the octet rule, suggests that the highest possible bond order is three. This follows from the fact that the molecular orbital (MO) for diatomic molecules with the second lowest energy is an antibonding σ orbital. This is shown by the MO correlation diagram in Figure 1 where N_2_ is used as an example. It nicely explains why dimeric alkaline‐earth atoms Ae_2_ (Ae = Be–Ba) do not have a covalent electron‐pair bond. Diatomic C_2_ was previously suggested to have a quadruple bond based on the valence bond theory calculation [1, 2, 3], which was criticized by several groups [4, 5, 6, 7, 8, 9]. The controversy was solved when Laws et al. reported the results of a high‐resolution photoelectron imaging spectrum, which showed that the chemical bond in C_2_ originates from two π bonds without any accompanied σ bond [10]. This is consistent with the picture that emerges from the MO correlation diagram when the four orbitals with the lowest energy are occupied (Figure 1).

Chemical bonds of transition metals may have higher bond orders than three due to their use of valence d orbitals, and the formal bond orders up to six have been suggested [11]. The electronic structure of transition metal (TM) compounds is more complicated and often requires multireference methods. But CASSCF calculations of diatomic TM_2_, where the occupation of antibonding MOs is considered, showed that even the effective bond order may be higher than five [12].

It was recently shown that the heavier Ae atoms, Ca, Sr. Ba, behave like transition metals in chemical reactions following the 18‐electron rule for chemical bonding. The octa‐coordinated complexes Ae(CO)_8_ [13] and Ae(N_2_)_8_ [14] and the benzene (bz) complexes Ae(bz)_3_ [15] (Ae = Ca − Ba) were synthesized and spectroscopically identified in the gas phase and in inert matrices [16]. A systematic theoretical study has shown that the heavier Ae atoms prefer to use their (n)s and (n‐1)d orbitals as valence functions for covalent bonds [17]. In contrast, the lighter atoms Be and Mg employ only their (n)s and (n)p AOs, and they obey the octet rule in the formation of stable molecules. The difference in chemical bonding between the lighter Ae atoms Be, Mg, and the heavier homologues Ca, Sr. Ba came clearly to the fore when the diatomic molecules AeB^−^, AeC, AeN^−^, and AeF^−^ were calculated, and the bonding situation was analyzed with a variety of methods [18, 19, 20, 21]. Four bonding orbitals were identified in AeF^−^ (Ae = Ca, Sr, Ba), which give a formal bond order of four for the dative bonds Ae 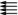 F^−^ where the electronic charge of F^−^ is donated into the vacant (n)p_σ_, (n‐1)d_σ_, and the degenerate (n‐1)d_π_ AOs of the Ae atom. In contrast, the lighter complexes, where Ae = Be, Mg, have only three dative bonds Ae 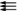 F^−^ since only the vacant (n)p_σ_ and the degenerate (n)p_π_ AOs are available as acceptor orbitals [19, 20].

**FIGURE 2:**
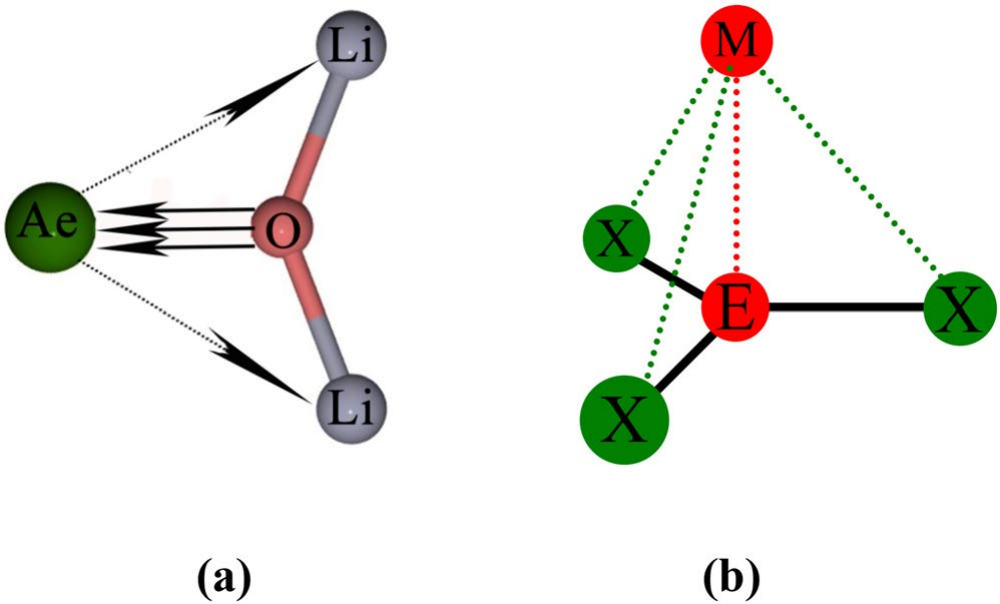
Schematic representation of (a) covalent bonding in AeOLi_2_, which consists of three (σ and 2 π) dative interactions Ae 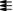 OLi_2_, and one bifurcated σ donation Ae → OLi_2_ and (b) the proposed nature of the bonding in collective interactions suggested in ref. [23], where the red dashed line indicates Coulomb repulsion and the green dashed line indicates electrostatic attraction.

A surprising result was obtained when the chemical bonds in AeOLi_2_, which is (valence) isoelectronic to AeF^−^, were analyzed. In contrast to the F^−^ anion, the donor fragment OLi_2_ has empty valence orbitals that can serve as acceptor orbitals. The doubly occupied (n)s AO of all Ae atoms is engaged in strong dative interactions with the vacant O‐Li_2_ σ* orbitals, which leads to quadruple bonds Ae 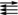 OLi_2_ even for Ae = Be, Mg [22]. The bonding situation of the combined donor‐acceptor interactions in AeOLi_2_ is schematically shown in Figure 2a. It bears similarities to the collective interactions recently proposed by Martin‐Pendas and Foroutan‐Nejad (Figure 2b) [23, 24, 25]. The dative bonds in AeF^−^ and AeOLi_2_ are very polar, and most of the electronic charge remains located at the donor atom. The triple and quadruple bonds in AeF^−^ have been questioned, because a topological analysis of the electronic structure suggests that the electron pair of the donor atom remains localized in the domain of one atom, and it should not be considered as a bond [26, 27]. But the assignment of an electron pair as a bonding orbital comes from the strength of the interatomic interactions and not from the shape of the orbital [28]. The significant contributions of the dative interactions to the overall strong bonds of AeF^−^ and AeOLi_2_ clearly suggest that there are triple and quadruple bonds in the molecules.

The unusual bonding in the complexes AeOLi_2_ led us to search for other molecules which might possess such a type of quadruple bond with Ae atoms, and which might be experimentally accessible. A possible set of candidates is the adducts Ae‐CLi_4_. CLi_4_ is experimentally known, and it has been observed in the gas phase [29], but its adducts, AeCLi_4_ (Ae = Be–Ba), have not been studied so far. Here we report on quantum chemical calculations using density functional theory and ab initio methods for the structure and stability of AeCLi_4_ and present the results of analyzing the bonding situation using various approaches.

## 2 Computational Methods

The geometry optimizations followed by the harmonic frequency calculations of the AeCLi_4_ (Ae = Be − Ba) complexes in their singlet spin states were performed at the BP86‐D3(BJ)/def2‐QZVPP level [30, 31, 32, 33]. Further reoptimization and frequency calculations were carried out at the CCSD(T)/def2‐QZVPP level [34]. These calculations were carried out using the GAUSSIAN 16 package [35]. The adaptive natural density partitioning (AdNDP) [36] analysis was performed using the Multiwfn program [37].

The atomic partial charges were computed employing various methods, viz., the natural bond orbital (NBO) [38], Hirshfeld [39], and Voronoi [40] approaches. While the NBO charge was calculated using the NBO7 program [41, 42], the Hirshfeld and Voronoi charges were computed using the Gaussian 16 and Multiwfn programs, respectively. The bond orders were evaluated using the Mayer approach and the Nalewajski‐Mrozek method [43, 44].

The bonding situations in these complexes were explored using the quantum theory of atoms in molecules (QTAIM) [45] and through an energy decomposition analysis (EDA) [46] together with the natural orbitals for chemical valence (NOCV) [47, 48] method by using the ADF 2020 program package [49, 50]. The EDA‐NOCV [51, 52] calculations were carried out at the BP86‐D3(BJ)/TZ2P‐ZORA//BP86‐D3(BJ)/def2‐QZVPP level [53, 54, 55]. In this analysis, the intrinsic interaction energy (Δ*Ε*_int_) between two fragments can be divided into three energy components as follows:

(1) $$\Delta {Ε}_{\mathrm{int}}=\Delta E_{\text{elstat}}+\Delta E_{\text{Pauli}}+\Delta E_{\mathrm{orb}}+\Delta E_{\text{disp}}$$

The electrostatic Δ*E*_elstat_ term represents the quasiclassical electrostatic interaction between the unperturbed charge distributions of the prepared fragments, whereas the Pauli repulsion, Δ*E*_Pauli_, corresponds to the energy change associated with the transformation from the superposition of the unperturbed electron densities of the isolated fragments to the wavefunction that properly obeys the Pauli principle through explicit antisymmetrization and renormalization of the production wavefunction. The orbital term Δ*E*_orb_ is originated from the mixing of orbitals, charge transfer, and polarization between the isolated fragments. Since D3(BJ) was used, it also computes the dispersion contribution (Δ*E*_disp_) to the overall interaction between the fragments.

The EDA‐NOCV enables the partition of the total Δ*E*_orb_ into pairwise contributions of the orbital interactions that are very important to get a complete picture of the bonding. The charge deformation Δ*ρ*_*k*_(*r*), resulting from the mixing of the orbital pairs 𝜓_*k*_(*r*) and 𝜓_−*k*_(*r*) of the interacting fragments, presents the amount and the shape of the charge flow due to the orbital interactions (Equation 2), and the associated energy term Δ*E*_orb_ provides the size of stabilizing orbital energy originated from such interaction (Equation 3).
(2)

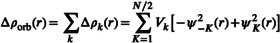

(3) $$\begin{array}{c} \Delta E_{\mathrm{orb}}=\sum_{k}\Delta E_{k}^{\mathrm{orb}}=\sum_{k=1}^{N/2}\nu_{k}\left[-F_{-k}^{\mathrm{TS}}+F_{k}^{\mathrm{TS}}\right]△E_{\mathrm{orb}}=\sum_{k}△E_{\mathrm{orb}}^{k}\\ \;=\sum_{k}\nu_{k}\left[-F_{-k,-k}^{\mathrm{TS}}+F_{K.K}^{\mathrm{TS}}\right] \end{array}$$

More details about the EDA‐NOCV method and its application are given in pertinent review articles [56, 57, 58, 59, 60, 61, 62].

## 3 Geometries and Energies

**FIGURE 3:**
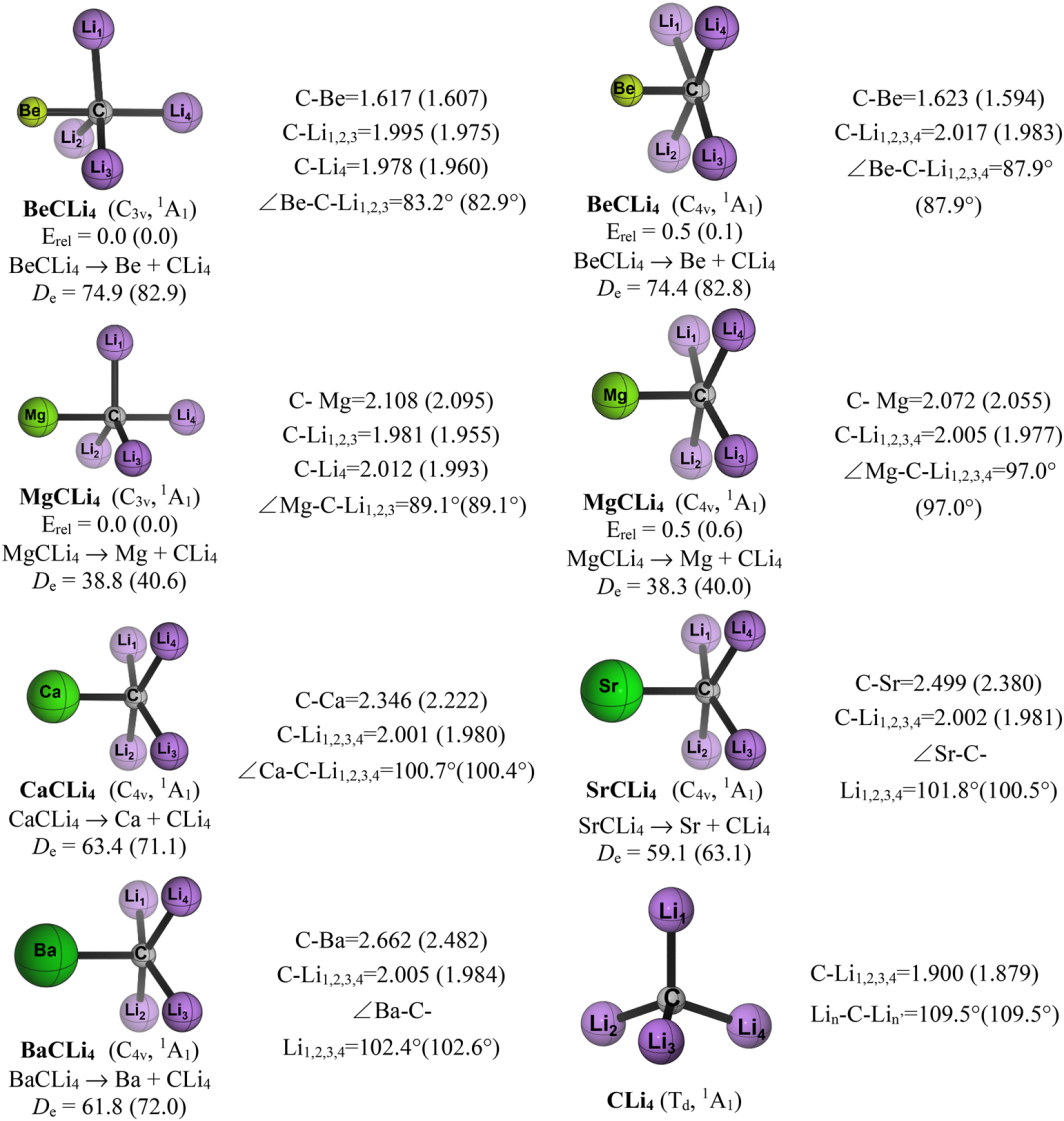
The minimum energy geometries of AeCLi_4_ (Ae = Be–Ba) computed at the CCSD(T) and BP86‐D3(BJ) (in parentheses) levels with def2‐QZVPP basis set. The bond lengths are given in Å and the bond angles in degrees. The bond dissociation energies (*D*_e_) are in kcal/mol.

Figure 3 shows the minimum energy isomers of AeCLi_4_ complexes at the CCSD(T)/def2‐QZVPP and BP86‐D3(BJ)/def2‐QZVPP levels of theory in the electronic singlet state. Geometry optimizations of AeCLi_4_ in the electronic triplet states give structures that are significantly higher in energy (see Table S1). They are not further considered in our work. Tables S3–S5 give the coordinates and energies of all calculated species.

For Ae = Be and Mg, there are, at both levels of theory, two almost isoenergetic isomers on the potential energy surfaces (PES). The lowest‐energy isomer has a trigonal bipyramidal geometry (*C*_3*v*_ symmetry), and the slightly higher‐lying form has a square pyramidal geometry (*C*_4*v*_ symmetry). The energy difference between the two forms is < 1 kcal/mol. The transition state for the interconversion of the two isomers is also very small (< 3 kcal/mol, see Figure S1). It means that the actual geometries of BeCLi_4_ and MgCLi_4_ are quite flexible.

In contrast, for the heavier complexes (Ae = Ca–Ba), only the square pyramidal form with *C*_4*v*_ geometry is an energy minimum structure. The optimized structures with *C*_3*v*_ symmetry constraint have two imaginary frequencies that lead to the *C*_4*v*_ isomer. The calculated geometry of CLi_4_ (*T*_d_) shows that the C‐Li bonds become longer in the Ae‐CLi_4_ complexes. Note that the axial C‐Li^4^ bond in BeCLi_4_ (*C*_3*v*_) is shorter than the equatorial bonds C‐Li^1,2,3^, whereas the axial C‐Li^4^ bond in MgCLi_4_ (*C*_3*v*_) is longer than the equatorial bonds C‐Li^1,2,3^ (Figure 3). There are further peculiar features of the calculated geometries. The basal C‐Li^1,2,3,4^ bonds of the square pyramidal (*C*_4*v*_) structures of AeCLi_4_ are tilted away from the Ae atom (bond angle Ae‐C‐Li > 90°) except for BeCLi_4_, which has bond angles of 87.9°. We do not discuss this subtle detail further since it is found for the energetically higher‐lying isomer.

Figure 1 also shows the bond dissociation energies (BDEs) for the reaction AeCLi_4_ → Ae + CLi_4_, which indicate the strength of the Ae‐CLi_4_ bond. The calculated values at the CCSD(T)/def2‐QZVPP level suggest that Be‐CLi_4_ has the strongest bond (*D*_e_ = 74.9 kcal/mol), whereas Mg‐CLi_4_ has the weakest bond (*D*_e_ = 38.8 kcal/mol). The Ae‐CLi_4_ bonds of the heavier homologues have intermediate values that vary only slightly between Ca‐CLi_4_ (*D*_e_ = 63.4 kcal/mol), Sr‐CLi_4_ (*D*_e_ = 59.1 kcal/mol), and Ba‐CLi_4_ (*D*_e_ = 61.8 kcal/mol). The BDE values at the BP86‐D3(BJ)/def2‐QZVPP level are uniformly a bit higher. Table S2 gives the calculated vibrational frequencies and IR intensities of the AeCLi_4_ complexes, which will be helpful to identify the molecules. The wave numbers of the Ae‐C vibrational stretching mode are in the range of 1011 cm^−1^ for Ae = Be and 374 cm^−1^ for Ae = Ba, showing the usual trend of gradual decrease with the increase in the reduced mass of the bonded atoms.

We estimated the correlation energy contribution to the Ae‐CLi_4_ interactions by calculating the BDE at the Hartree‐Fock (HF/def2‐QZVPP) and CCSD(T)/def2‐QZVPP levels. Table 1 shows that the correlation component to the metal–ligand bond, which results from the difference ΔE between the CCSD(T) and HF values, is rather small in the lighter systems with Ae = Be, Mg—particularly in the beryllium complex—while it is significantly larger in the heavier systems with Ae = Ca, Sr, Ba. This is an interesting result because a previous study of the alkaline complexes E‐BX_3_ (E = Li, Na, K) where X is a halogen atom showed that the correlation energy contribution to all systems is uniformly high [24]. We think that the large contributions of the correlation component in the heavier alkaline earth compounds come from the low‐lying (n‐1)d AOs of the metal atoms.

**TABLE 1:** Calculated bond dissociation energies (*D*_e_, kcal/mol) of the structure of AeCLi_4_ (Ae = Be, Mg, Ca, Sr. Ba) CLi_4_ computed at the Hartree‐Fock (HF) and CCSD(T) levels with def2‐QZVPP basis set and energy differences Δ*E*.

AeCLi_4_ → Ae + CLi_4_			
	HF	CCSD(T)	Δ*E*
BeCLi_4_ (C_3v_)	71.5	74.9	3.4
BeCLi_4_ (C_4v_)	71.3	74.4	3.1
MgCLi_4_ (C_3v_)	30.8	38.8	8.0
MgCLi_4_ (C_4v_)	30.8	38.3	7.5
CaCLi_4_ (C_4v_)	37.1	63.4	26.3
SrCLi_4_ (C_4v_)	34.3	59.1	24.8
BaCLi_4_ (C_4v_)	40.1	61.8	21.7

## 4 Bonding Analysis

**FIGURE 4:**
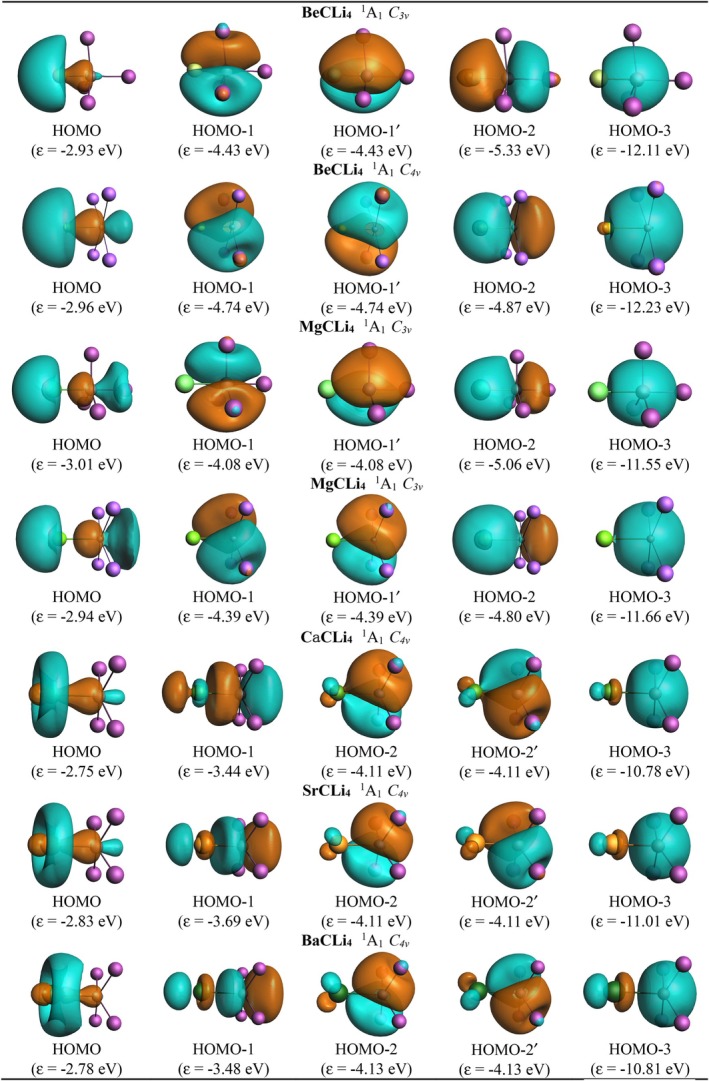
Shape of the five highest lying occupied Kohn‐Sham MOs of AeCLi_4_ at the BP86‐D3(BJ)/def2‐QZVPP level.

We analyzed the electronic structure of the penta‐coordinated carbon complexes AeCLi_4_ with a variety of methods focusing on the nature of the Ae‐CLi_4_ bonds. Visual inspection of the five occupied valence MOs already provides some interesting insight on the bonding situation. They are shown in Figure 4. The highest occupied MO (HOMO) is, in all cases, mainly localized at the Ae atom with a bonding contribution to the carbon atom. Interestingly, the HOMOs of the *C*_3*v*_ and *C*_4*v*_ isomers of BeCLi_4_ and MgCLi_4_ appear very similar. It is an sp_σ_ hybridized orbital at Ae and C (Ae = Be, Mg). In contrast, the HOMOs of the heavier *C*_4*v*_ complexes of AeCLi_4_ (Ae = Ca, Sr, Ba) have an sd_z_2 hybridized orbital at Ae. The HOMO‐1 and HOMO‐1′ are, for the lighter homologues BeCLi_4_ and MgCLi_4_, a pair of energetically degenerate Ae‐CLi_4_ π‐bonding orbitals, which comprise the *p*_π_ AOs of Ae atoms. The HOMO‐2 of the latter species is another Ae‐CLi_4_ σ‐bonding orbital, whereas the HOMO‐3 is mainly a 2 s core AO at carbon.

The occupied valence orbitals of the heavier AeCLi_4_ homologues with Ae = Ca, Sr, Ba exhibit related features but show specific differences compared with the lighter systems. The HOMO is also mainly a lone‐pair MO at the Ae atom with a bonding component to carbon, but the shape shows that it is an sd_z_2 hybridized orbital. There is also a degenerate pair of π‐bonding orbitals, HOMO‐2 and HOMO‐2′, which comprise the d_π_ AOs of Ae atoms that are strongly polarized toward CLi_4_. The second σ‐bonding MO appears as HOMO‐1, which is also an sd_z_2 hybridized orbital at the Ae atom, but it is strongly polarized toward CLi_4_. The HOMO‐3 is mainly a carbon 2 s AO with some minor contribution from the (n)p_σ_ AO of Ae.

**FIGURE 5:**
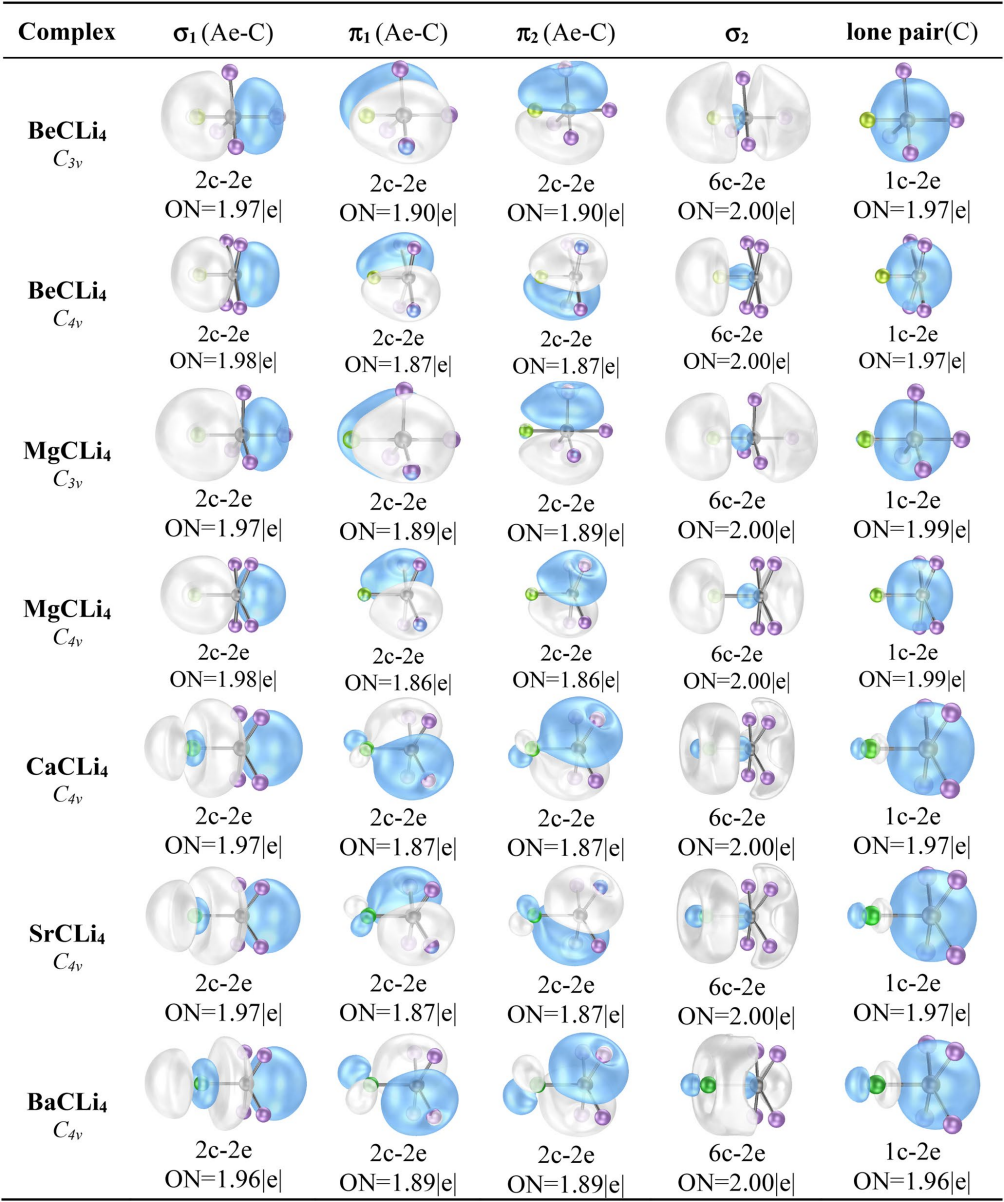
AdNDP results of AeCLi_4_ at the BP86‐D3(BJ)/def2‐QZVPP level showing the occupation numbers (ON) for 2c‐2e and 6c‐2e orbitals involving the Ae‐C moiety. Shape of the two‐center and six‐center orbitals of AeCLi_4_. Occupation numbers (ON) are in |e|.

The canonical MOs can be converted into localized MOs with the help of the AdNDP method introduced by Zubarev and Boldyrev [36]. It is particularly suited for delocalized bonds, which are found in AeCLi_4_. The shapes of the five AdNDP orbitals are shown in Figure 5. There is a 2‐center 2‐electron (2c‐2e) σ bond orbital for all systems with an occupation number (ON) close to 2. There is a second σ bonding orbital which extends over all atoms (6c‐2e) with ON = 2.0. Furthermore, there are two 2c‐2e π‐bonding orbitals with ON between 1.86 and 1.90. Finally, there is a carbon centered 1c‐2e orbital with ON = 1.97–1.99. The AdNDP method supports the visual impression of the canonical MOs that all AeCLi_4_ complexes have four Ae‐CLi_4_ bonds.

The AdNDP results suggest the number and the shape of the Ae‐CLi_4_ bonds, but they do not provide information about the strength of the chemical bonds. This is available from the EDA‐NOCV method, where the interaction energies (Δ*E*_int_) between neutral Ae atoms and the CLi_4_ fragments are calculated. The numerical results for the *C*_3*v*_ and *C*_4*v*_ isomers of BeCLi_4_ and MgCLi_4_ are given in Table 2. The Δ*E*_int_ values between the fragments in the frozen geometries are higher for the *C*_4*v*_ isomers than for the *C*_3*v*_ form, but the preparation energy of the CLi_4_ fragments in the former species is also higher than in the latter, which leads to a slightly lower energy of the *C*_3*v*_ form. The covalent (orbital) interaction, Δ*E*_orb_, is in both isomers of BeCLi_4_ a bit stronger than the electrostatic (Coulomb) interaction, Δ*E*_elstat_. The latter term dominates the attractive interactions in MgCLi_4_, but the covalent bonding still contributes ~37% of the total attraction.

**TABLE 2:** EDA‐NOCV results of BeCLi_4_ and MgCLi_4_ with *C*_3v_ and *C*_4v_ geometries at the BP86‐D3(BJ)/TZ2P‐ZORA//BP86‐D3(BJ)/def2‐QZVPP level using Ae (ns^2^, 1S) + CLi_4_ (^1^A_1_) as interacting fragments. Energy values are given in kcal/mol.

Energy	Orbital interaction	Ae (ns^2^, ^1^S) + CLi_4_ (^1^A_1_)			
		BeCLi_4_/*C*_3v_	BeCLi_4_/*C*_4v_	MgCLi_4_/*C*_3v_	MgCLi_4_/*C*_4v_
Δ*E*_int_		−86.6	−96.0	−44.6	−52.3
Δ*E*_Pauli_		280.3	322.0	142.8	153.8
Δ*E*_disp_ ^a^		−3.1 (0.9%)	−3.2 (0.8%)	−4.2 (2.2%)	−4.4 (2.1%)
Δ*E*_elstat_ ^a^		−180.3 (49.1%)	−188.4 (45.1%)	−114.5 (61.1%)	−122.8 (59.6%)
Δ*E*_orb_ ^a^		−183.5 (50.0%)	−226.4 (54.2%)	−68.7 (36.7%)	−78.9 (38.3%)
Δ*E*_orb(1)_ ^b^	Ae → CLi_4_ σ donation	−96.8 (52.7%)	−154.5 (68.2%)	−34.5 (50.2%)	−47.3 (59.9%)
Δ*E*_orb(2)_ ^b^	Ae ← CLi_4_ σ backdonation	−28.1 (15.3%)	−13.5 (6.0%)	−13.1 (19.1%)	−10.9 (13.8%)
Δ*E*_orb(3)_ ^b^	Ae ← CLi_4_ π backdonation	−28.2 (15.4%)	−27.2 (12.0%)	−10.1 (14.7%)	−9.8 (12.4%)
Δ*E*_orb(4)_ ^b^	Ae ← CLi_4_ π′ backdonation	−28.2 (15.4%)	−27.2 (12.0%)	−10.1 (14.7%)	−9.8 (12.4%)
Δ*E*_orb(rest)_ ^b^		−2.2 (1.2%)	−4.0 (1.8%)	−0.9 (1.3%)	−1.1 (1.4%)
Δ*E*_prep_		4.4	13.3	4.3	13.0
*D*_e_ = −(Δ*E*_int_ + Δ*E*_prep_)		82.2	82.7	40.3	39.3

^a^ The percentage contribution with respect to total attraction is given in parentheses.

^b^ The percentage contribution in parentheses is given with respect to total orbital interaction.

**FIGURE 6:**
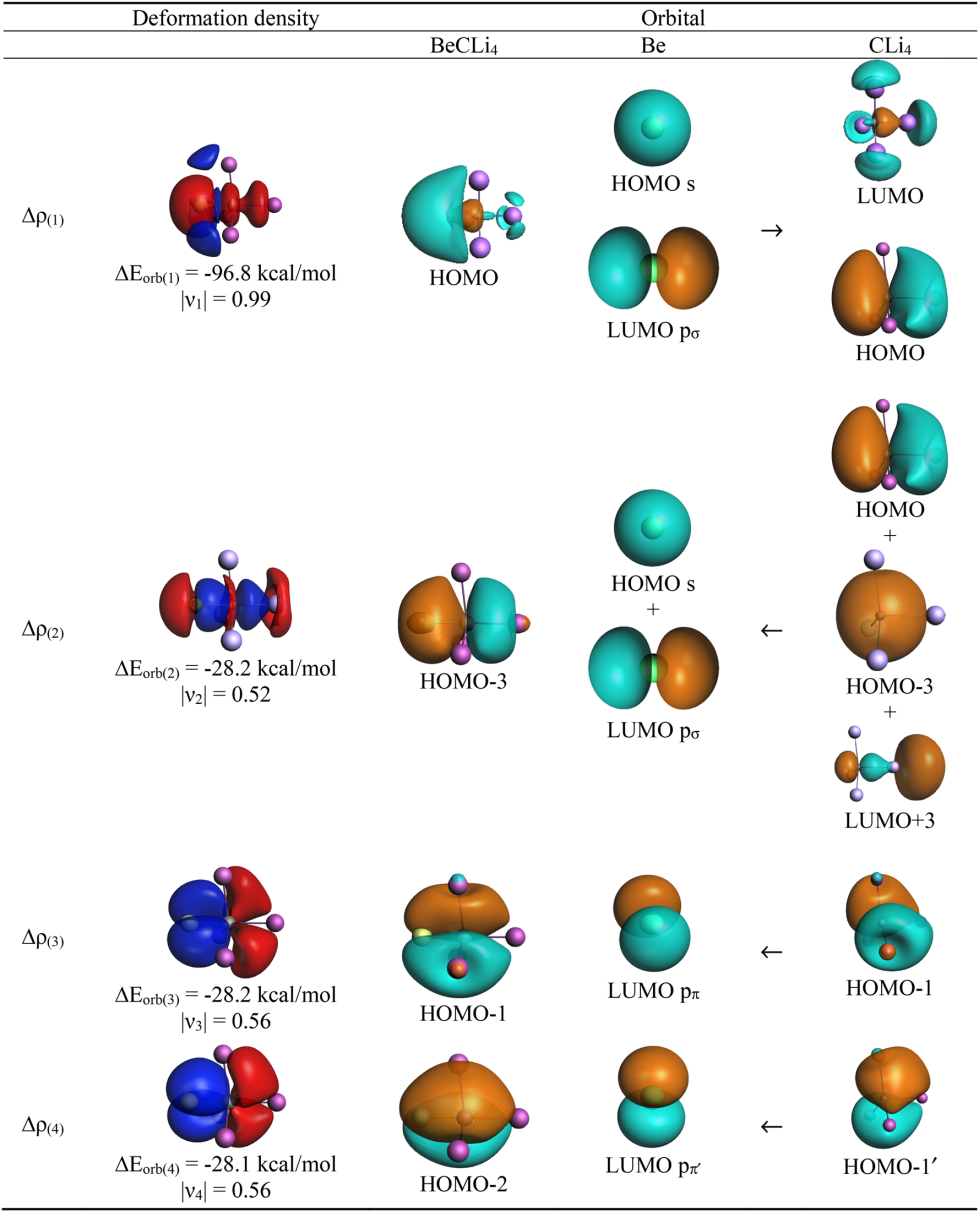
Plot of the deformation densities, ∆*ρ*_(1)–(4)_ shown as the sum of α and β electronic charge corresponding to ∆*E*_orb(1)–(4)_ and the related interacting orbitals in the singlet states of BeCLi_4_ (*C*_3v_) at the BP86‐D3(BJ)/TZ2P‐ZORA//BP86‐D3(BJ)/def2‐QZVPP level using Be (2s^2^, ^1^S) + CLi_4_ (^1^A_1_) as interacting fragments. The eigenvalues ν indicate the size of the charge flow. The direction of charge flow is red → blue. The isovalue for ∆*ρ*_(1,3,4)_ is 0.001 au and the isovalue for ∆*ρ*_(2)_ is 0.0003 au.

**FIGURE 7:**
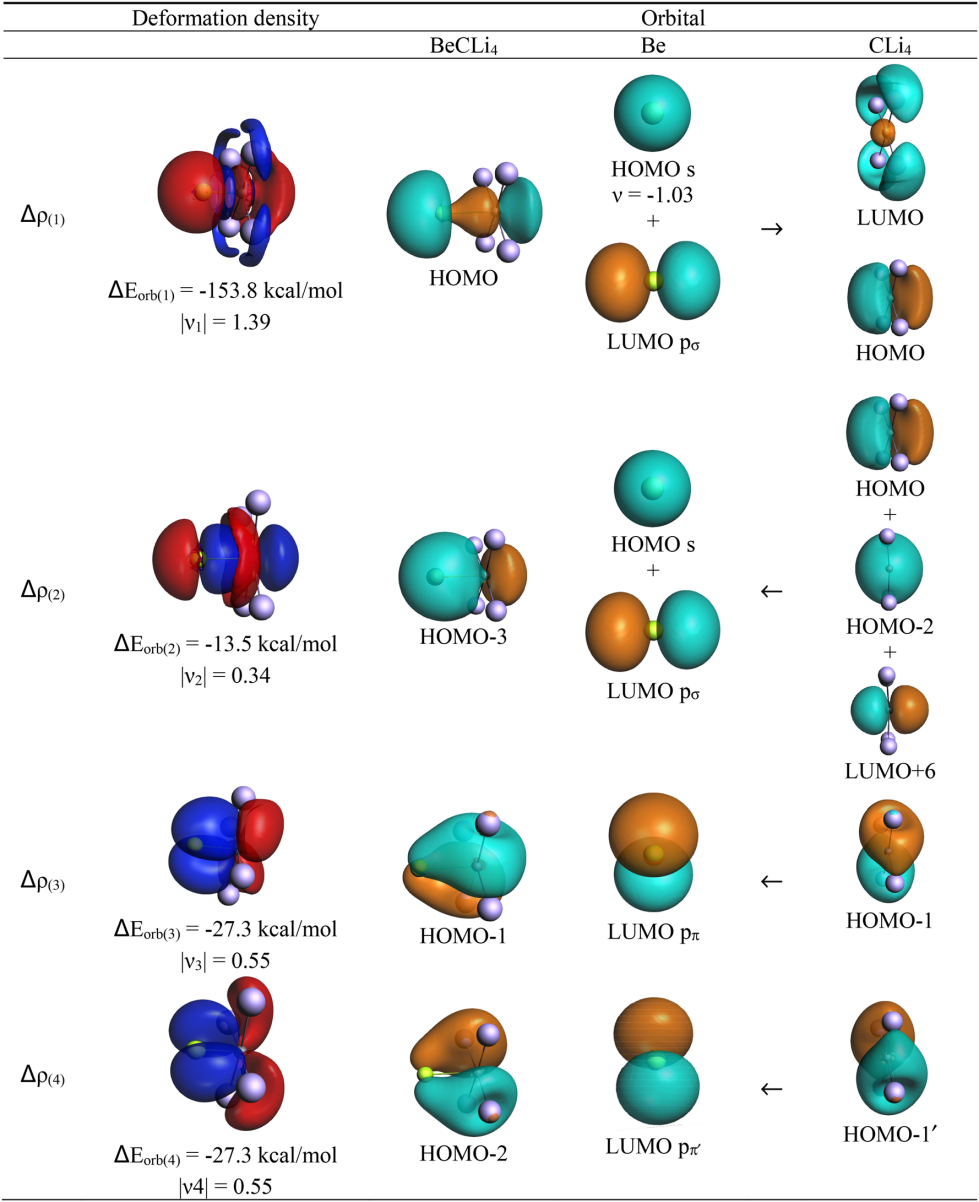
Plot of the deformation densities, ∆*ρ*_(1)–(4)_ shown as the sum of α and β electronic charge corresponding to ∆*E*_orb(1)–(4)_ and the related interacting orbitals in the singlet states of BeCLi_4_ (*C*_4v_) at the BP86‐D3(BJ)/TZ2P‐ZORA//BP86‐D3(BJ)/def2‐QZVPP level using Be (2s^2^, ^1^S) + CLi_4_ (^1^A_1_) as interacting fragments. The eigenvalues ν indicate the size of the charge flow. The direction of charge flow is red → blue. The isovalue for ∆*ρ*_(1,3,4)_ is 0.001 au and the isovalue for ∆*ρ*_(2)_ is 0.0003 au.

The most interesting information of the EDA‐NOCV results comes from the breakdown of the total covalent term, Δ*E*_orb_ into the pair‐wise orbital interactions, Δ*E*_orb(n)_. There are four major contributions, Δ*E*_orb(1)_ − Δ*E*_orb(4)_, which comprise > 98% of Δ*E*_orb_. The remaining terms, Δ*E*_orb(rest)_, are due to core orbital relaxation. The nature of the pair‐wise orbital interactions, Δ*E*_orb(1)_ − Δ*E*_orb(4)_, becomes obvious by the visual inspection of the associated deformation densities and the connected fragment orbitals, which are shown for BeCLi_4_ in Figure 6 (*C*_3*v*_ isomer) and Figure 7 (*C*_4*v*_ isomer). The deformation densities and connected orbitals of MgCLi_4_ are very similar. They are shown in Figures S2 and S3. The strongest orbital interaction, Δ*E*_orb(1)_, in the *C*_3*v*_ and *C*_4*v*_ isomers comes from the donation of the occupied (n)s AO of Be (Mg) into the LUMO of CLi_4_, which has its largest components at the Li atoms. There are further minor contributions from vacant and occupied fragment orbitals, but the overall description of the strongest bond, Δ*E*_orb(1)_, is a Be(Mg) → CLi_4_ σ‐donation toward the equatorial Li atoms. This is evident in the blue region where charge accumulates between Ae and Li atoms (Figures 6, 7, S2, and S3). It is a similar situation to that found in AeOLi_2_, which resembles the collective interactions recently proposed by Martin‐Pendas and Foroutan‐Nejad. The stabilization energy of Δ*E*_orb(1)_ provides more than half of the total orbital interactions in both isomers, where the contribution of the *C*_4*v*_ isomer is higher than in the *C*_3*v*_ form (Table 2).

The orbital interactions Δ*E*_orb(2)_ in the *C*_3*v*_ and *C*_4*v*_ isomers are due to reverse σ‐donation Be(Mg) ← CLi_4_ from the HOMO of CLi_4_ to the vacant (n)p_σ_ AO of Ae atom, along with some polarization. The orbital interaction Δ*E*_orb(2)_ is clearly weaker than Δ*E*_orb(1)_, but it is not negligible. Unlike Δ*E*_orb(1)_, the contribution of Δ*E*_orb(2)_ in the *C*_4*v*_ isomer is smaller than in the *C*_3*v*_ form. The orbital interactions, Δ*E*_orb(3)_ and Δ*E*_orb(4)_, are due to degenerate π‐backdonation Be(Mg) ← CLi_4_ into the vacant (n)p_π_ AOs of the metal atom.

**FIGURE 8:**
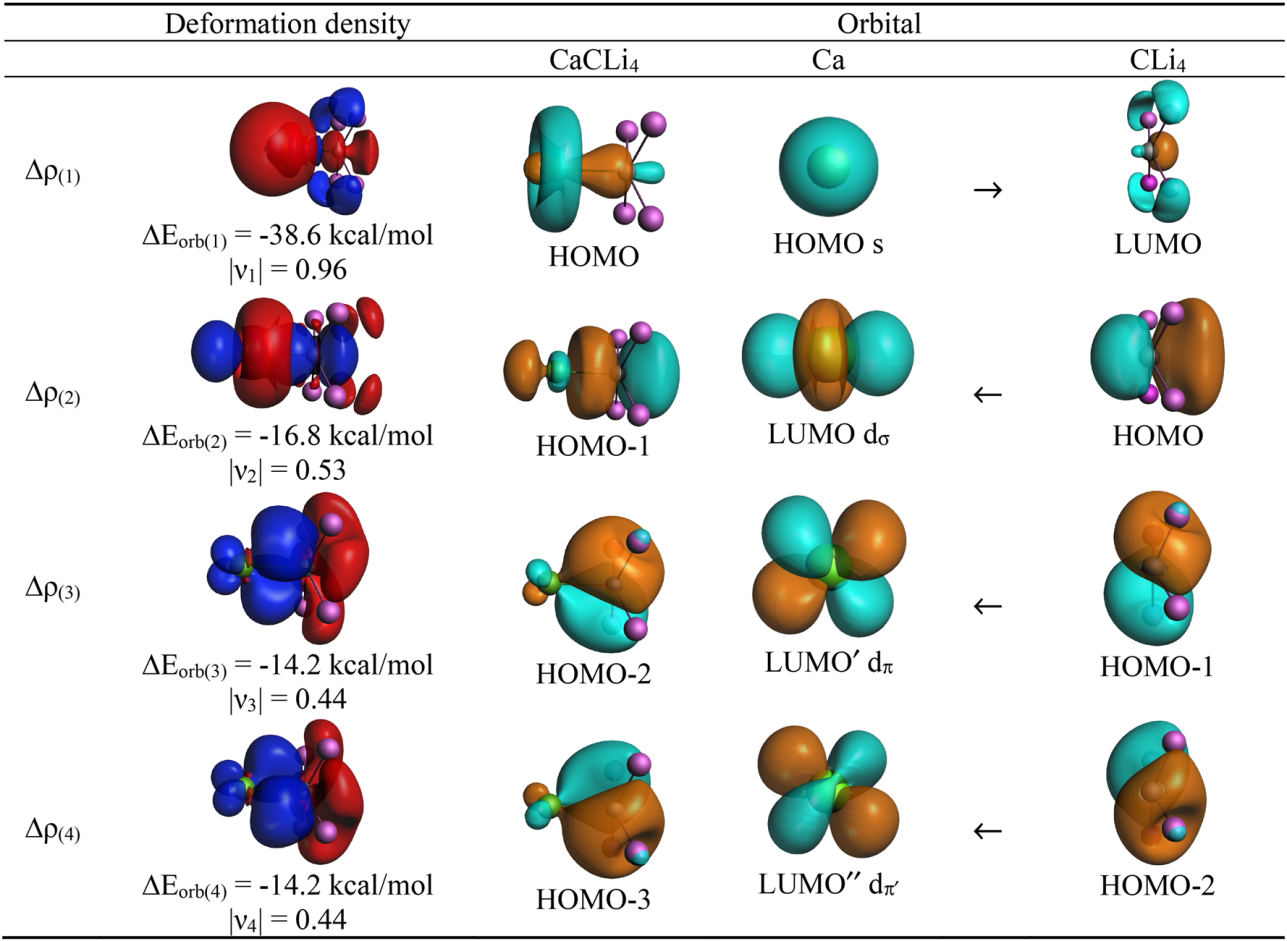
Plot of the deformation densities, ∆*ρ*_(1)–(4)_ shown as the sum of α and β electronic charge corresponding to ∆*E*_orb(1)–(4)_ and the related interacting orbitals in the singlet states of CaCLi_4_ at the BP86‐D3(BJ)/TZ2P‐ZORA//BP86‐D3(BJ)/def2‐QZVPP level using Ca (4s^2^, ^1^S) + CLi_4_ (^1^A_1_) as interacting fragments. The eigenvalues ν indicate the size of the charge flow. The direction of charge flow is red → blue. The isovalue for ∆*ρ*_(1)_ is 0.001 au for ∆*ρ*_(2)–(4)_ it is 0.0005 au.

The orbital interactions of the heavier homologues of AeCLi_4_ (Ae = Ca, Sr, Ba) exhibit a similar pattern to that of the lighter species. Table 3 shows the numerical results, and Figure 8 displays the deformation densities and orbitals of CaCLi_4_. The deformation densities and orbitals of the heavier complexes SrCLi_4_ and BaCLi_4_ are very similar, and they are shown in Figures S4 and S5. The strongest orbital interaction Δ*E*_orb(1)_ is a Ae → CLi_4_ (Ae = Ca, Sr, Ba) σ‐donation from the occupied (n)s AO of Ae atom with some contribution of the (n‐1)d_z_2 AO into the LUMO of CLi_4_. The shape of the deformation density indicates that the bonding interaction involves the lithium atoms, as in the case of the lighter homologues. This orbital interaction, Δ*E*_orb(1)_, is clearly an example of the collective interactions proposed by Martin‐Pendas and Foroutan‐Nejad [23, 24, 25]. The orbital interaction, Δ*E*_orb(2)_, comes from a reverse σ‐donation Ae ← CLi_4_ from the HOMO of CLi_4_ to the vacant (n‐1)d_z_2 AO of Ae atom along with some polarization. The orbital interactions, Δ*E*_orb(3)_ and Δ*E*_orb(4)_, are due to degenerate π‐backdonation Ae ← CLi_4_ into the vacant (n‐1)d_π_ AOs of the metal atom (Figures 8, S4, and S5).

**TABLE 3:** EDA results of AeCLi_4_ (Ae = Ca, Sr, Ba) at the BP86‐D3(BJ)/TZ2P‐ZORA//BP86‐D3(BJ)/def2‐QZVPP level using Ae (ns^2^, ^1^S) + CLi_4_ (^1^A_1_) as interacting fragments. Energy values are given in kcal/mol.

Energy	Orbital interaction	Ae (ns^2^, ^1^S) + CLi_4_ (^1^A_1_)		
		CaCLi_4_	SrCLi_4_	BaCLi_4_
Δ*E*_int_		−71.9	−64.3	−75.0
Δ*E*_Pauli_		165.4	155.1	171.3
Δ*E*_disp_ ^a^		−5.0 (2.1%)	−4.9 (2.2%)	−5.1 (2.0%)
Δ*E*_elstat_ ^a^		−147.1 (62.0%)	−139.8 (63.8%)	−152.9 (62.1%)
Δ*E*_orb_ ^a^		−85.2 (35.9%)	−74.7 (34.0%)	−88.3 (35.9%)
Δ*E*_orb(1)_ ^b^	Ae → CLi_4_ σ donation	−38.6 (45.3%)	−33.4 (44.7%)	−34.9 (39.5%)
Δ*E*_orb(2)_ ^b^	Ae ← CLi_4_ σ backdonation	−16.9 (19.8%)	−15.4 (20.6%)	−21.2 (24.0%)
Δ*E*_orb(3)_ ^b^	Ae ← CLi_4_ π backdonation	−14.2 (16.7%)	−12.3 (16.5%)	−14.9 (16.9%)
Δ*E*_orb(4)_ ^b^	Ae ← CLi_4_ π backdonation	−14.2 (16.7%)	−12.3 (16.5%)	−14.9 (16.9%)
Δ*E*_orb(rest)_ ^b^		−1.3 (1.5%)	−1.3 (1.7%)	−2.4 (2.7%)
Δ*E*_prep_		13.0	13.1	13.2
*D*_e_ = −(Δ*E*_int_ + Δ*E*_prep_)		58.9	51.2	61.8

^a^ The percentage contribution with respect to total attraction is given in parentheses.

^b^ The percentage contribution in parentheses is given with respect to total orbital interaction.

Tables 2 and 3 also show the preparation energies Δ*E*_prep_, which come from the geometrical distortion of the CLi_4_ fragment from the optimized structure to the geometry in the complexes AeCLi_4_. The calculated values suggest a rather uniform small value of Δ*E*_prep_ 4.3–4.4 kcal/mol for the lighter complexes where Ae = Be, Mg, but larger values of 13.0–13.2 kcal/mol for the heavier systems where Ae = Ca, Sr, Ba. Note that the calculated *D*_e_ values given in Tables 2 and 3 using BP86‐D3(BJ) are slightly different from the results given in Figure 3, because the former values are obtained with Slater functions whereas the latter ones are calculated using Gaussian basis sets.

The pattern of the orbital interaction in the AeCLi_4_ complexes is qualitatively the same for all species Ae = Be − Ba. There are two σ‐donor bonds in reverse directions Ae → CLi_4_ and Ae ← CLi_4_, and a set of degenerate π‐backdonation Ae ← CLi_4_. The difference between the lighter species with Ae = Be, Mg, and the heavier homologues with Ae = Ca, Sr, Ba comes from the valence orbitals of the metal atom. The atoms Be, Mg use their (n)s and (n)p AOs, whereas the heavier atoms Ca, Sr. Ba use their (n)s and (n‐1)d AOs for bonding. It has previously been shown that the heavier alkaline earth atoms built covalent bonds through s/d hybridized orbitals like transition metals [13, 14, 15, 16, 17, 18, 19, 20, 21]. The difference between the lighter homologues with Be, Mg and the heavier systems with Ca, Sr. Ba comes among others to the fore by the different Δ*E*_prep_ values.

**FIGURE 9:**
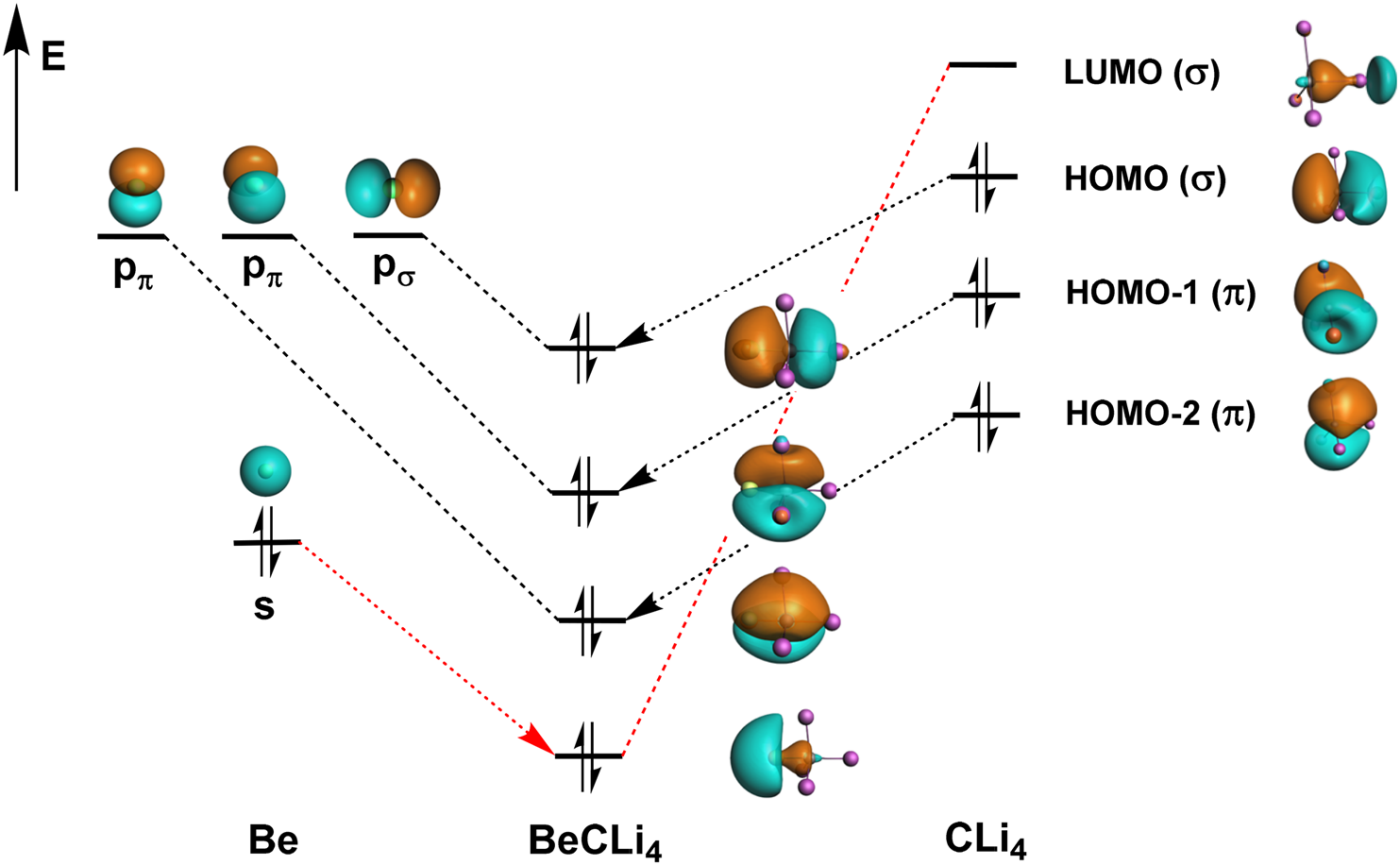
The MO correlation diagram of BeCLi_4_ from Be and CLi_4_ fragments.

How is it possible that the main‐group atoms, Be, Mg, are able to form four bonds with their sp. valence orbitals? The MO correlation diagram shown in Figure 1 suggests that the maximum bond order for main‐group atoms can only be three. But the orbital correlation diagram of dative bonds differs from the diagram for electron‐sharing bonds. Figure 9 shows the dative interactions between Be atom with the electron configuration 2s^2^2p_σ_^0^2p_π_^0^2p_π’_^0^ and the CLi_4_ fragments with the LUMO, HOMO, HOMO‐1, and HOMO‐1^′^ orbitals. There is one dative bond for the donation Be(2s^2^) → CLi_4_(LUMO) and three dative bonds for backdonation Be(2p_σ_^0^) ← CLi_4_(HOMO), Be(2p_π_^0^) ← CLi_4_(HOMO‐1), Be(2p_π’_^0^) ← CLi_4_(HOMO‐1′), which built a quadruple bond between the two fragments. A similar diagram applies for MgCLi_4_. Thus, it is possible that a quadruple bond is formed between two main‐group atoms if the bonding interactions come from dative bonds.

The eigenvalues ν of the deformation densities, which are shown in Figures 6, 7, 8 and S2–S5, are a measure of the associated charge flow. The numerical values suggest that the charge donation from the alkaline earth atoms Ae → CLi_4_ and the backdonation Ae ← CLi_4_ have a similar magnitude. Table 4 shows the computed atomic partial charges using NBO, QTAIM, Hirshfeld, and Voronoi methods. The NBO method gives a highly positive charge on the Ae atom and an extremely high negative charge (> −3) at the carbon atom, whereas the Hirshfeld and Voronoi methods give a negative charge on Be, almost zero charge on Mg, and a small positive charge (0.07–0.21 *e*) on the heavier Ae atoms. The latter methods give also a physically reasonable negative charge (< −1) at carbon. The highly positive charges of the Ae atom and the unphysically large negative charge at carbon, suggested by the NBO method, come from the failure to recognize the vacant 2p AOs of Li and the (n)p and (n‐1)d AOs of Ae as genuine valence orbitals. The NBO method considers only those orbitals as valence orbitals, which are occupied in the electronic ground state of the atoms [38]. Vacant AOs are considered as Rydberg orbitals, which are treated with lower weight in the NBO algorithm. Figure 4 shows that the occupied valence orbitals of AeCLi_4_ exhibit a significant sp. or sd hybridization at Ae atoms in the Ae‐CLi_4_ σ‐region and p_π_ or d_π_ contributions in the π region. The NBO algorithm ignores the hybridization at the Li and Ae atoms and gives a distorted picture of the bonding situation. This has been noted in previous studies of alkaline earth molecules [18, 19, 20, 21]. According to the NBO bond orbitals, there are no Ae‐CLi_4_ bonds but only a lone‐pair orbital at Ae.

**TABLE 4:** Partial charges (*q*) and bond orders (*P*) of AeCLi_4_ (Ae = Be, Mg, Ca, Sr. Ba) computed at the BP86‐D3(BJ)/def2‐QZVPP level.

Molecules	*q*_Ae_ */q*_C_				*P*_Ae‐C_	
	NBO	QTAIM	Hirshfeld	Voronoi	Mayer	N–M
BeCLi_4_/C_3v_	0.67/−3.44	0.56/−3.74	−0.18/−0.66	−0.23/−0.59	0.93	1.59
BeCLi_4_/C_4v_	0.64/−3.37	0.67/−3.82	−0.17/−0.64	−0.20/−0.58	0.92	1.58
MgCLi_4_/C_3v_	0.64/−3.47	0.36/−3.52	−0.02/−0.68	0.01/−0.64	0.70	1.24
MgCLi_4_/C_4v_	0.69/−3.42	0.47/−3.57	0.00/−0.67	0.04/−0.63	0.77	1.28
CaCLi_4_/C_4v_	0.72/−3.31	0.60/−3.64	0.09/−0.69	0.17/−0.67	0.67	1.40
SrCLi_4_/C_4v_	0.76/−3.32	0.60/−3.62	0.14/−0.69	0.21/−0.67	0.91	1.41
BaCLi_4_/C_4v_	0.80/−3.26	0.61/−3.55	0.07/−0.69	0.20/−0.74	0.85	1.58

The QTAIM partial charges are similar to the NBO charges, which seems to support the NBO picture of the chemical bonds. But the partitioning of the molecular structures is based on the topological analysis of the electronic structure, where the curvature of the electronic charge determines the zero‐flux surfaces, which separate the atomic regions. This leads to highly charged atoms, where the electronic charge of polar bonds is exclusively assigned to the more electronegative atom, ignoring the contribution of the electropositive atom.

The calculated partial charges by the Hirshfeld and Voronoi methods suggest that the Be atom is a weak acceptor, the donation and backdonation at Mg nearly cancel each other, whereas the heavier Ae atoms Ca, Sr., and Ba are slightly stronger donors than acceptors. This is in agreement with the trend of the electronegativities, which continuously decrease from Be (1.57) to Ba (0.89) using the Pauling scale. Table 4 also gives the Mayer bond orders [43] for the Ae‐Li_4_ bonds. The low values between 0.7 and 0.9 do not contradict quadruple bonding. Polar bonds have significantly lower bond orders than unipolar bonds [28]. The bond orders N–M calculated by the Nalewajski‐Mrozek method [44] are much higher because they comprise the electrostatic contributions to the bonding interactions. This agrees with the EDA‐NOCV results, which suggest that Coulombic interactions provide a significant part of the Ae‐CLi_4_ attraction.

The crucial role of the electropositive lithium atoms, which provide energetically low‐lying vacant orbitals that serve as acceptor orbitals for strong dative Ae → CLi_4_ interactions and enhance the electrostatic attraction between Ae and carbon, becomes clearly apparent when considering the hydrogen‐containing parent systems AeCH_4_. Geometry optimization at the BP86‐D3(BJ)/def2‐QZVPP level of these species leads to dissociated Ae atoms and CH_4_.

**FIGURE 10:**
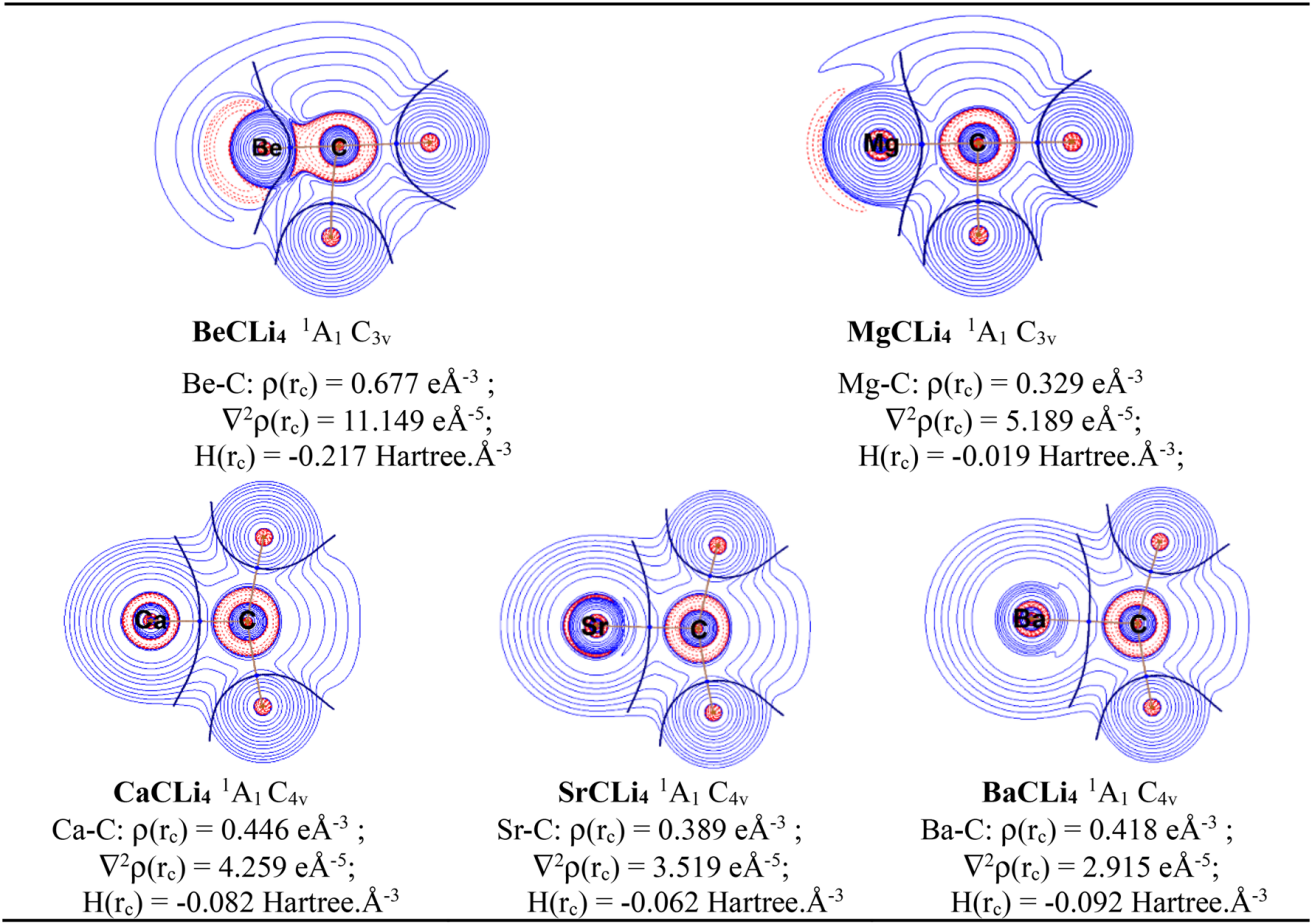
Laplacian distribution ∇^2^ *ρ*(*r*) of AeCLi_4_ (Ae = Be, Mg, Ca, Sr. Ba) at the CCSD/def2‐QZVPP//CCSD(T)/def2‐QZVPP level. Red lines indicate the areas of charge concentration (∇^2^ *ρ*(*r*) < 0), while blue lines show the areas of charge depletion (∇^2^ *ρ*(*r*) > 0). The black solid lines connecting the atomic nuclei are the bond paths. Blue dots are bond critical points (bcp). The thick lines which cross the bcp show the zero‐flux surfaces in the molecular plane that separate the atomic basins. The alkaline earth metal atoms are given at the left.

We also analyzed the electronic structure of the molecules with the QTAIM method [45]. Figure 10 shows the Laplacian distribution ∇^2^ *ρ*(*r*) of the energetically lowest lying complexes AeCLi_4_ at the CCSD/def2‐QZVPP//CCSD(T)/def2‐QZVPP level. The shape of the area of charge accumulation in the Ae‐C bonding region exhibits a significant distortion where Ae = Be, but there is highly symmetric charge distribution at carbon in the heavier complexes Ae = Mg − Ba. The energy density at the bond critical points (bcp) has a negative value H(*r*_*c*_) = −0.217 Hartree.Å^−3^ which, according to the criterion suggested by Cremer and Kraka, indicates a covalent bond [63]. However, the H(*r*_*c*_) values and the shape of the Laplacian distribution suggest closed‐shell (ionic) interactions between all atoms of the heavier complexes AeCLi_4_ where Ae = Mg − Ba. This is an artifact of the topological analysis of the electronic structure, in which the physical nature of the interatomic interaction resulting from the interference of the wave functions is ignored. In particular, the position of the bcp in polar bonds does not accurately reflect the nature of the chemical bond.

We calculated the dipole moments of the complexes AeCLi_4_, which provide further information about the electronic structure of the molecules. Note that dipole moments are vector quantities and should not be confused with atomic partial charges, which are scalar quantities. The direction of the dipole moment may not always agree with the atomic partial charges. Striking examples are the dipole moments of CO (−0.11 D) [64] and BF (−0.80 D) [65] where the minus sign indicates that the negative pole is on the less electronegative atoms C and B, respectively. Even larger values were reported for the alkaline earth molecules AeF^−^ (Ae = Be − Ba), which have values between −5.97 D (BeF^−^) and −1.78 D (BaF^−^), where the negative end is at the Ae atom [20]. The detailed analysis of the individual orbital components of the dipole moments in AeF^−^ showed that the electronic charge of the HOMO has a decisive influence on the overall dipole moment. It has been shown that the total dipole moment of a molecule can be broken down into individual orbital components [66].

**FIGURE 11:**
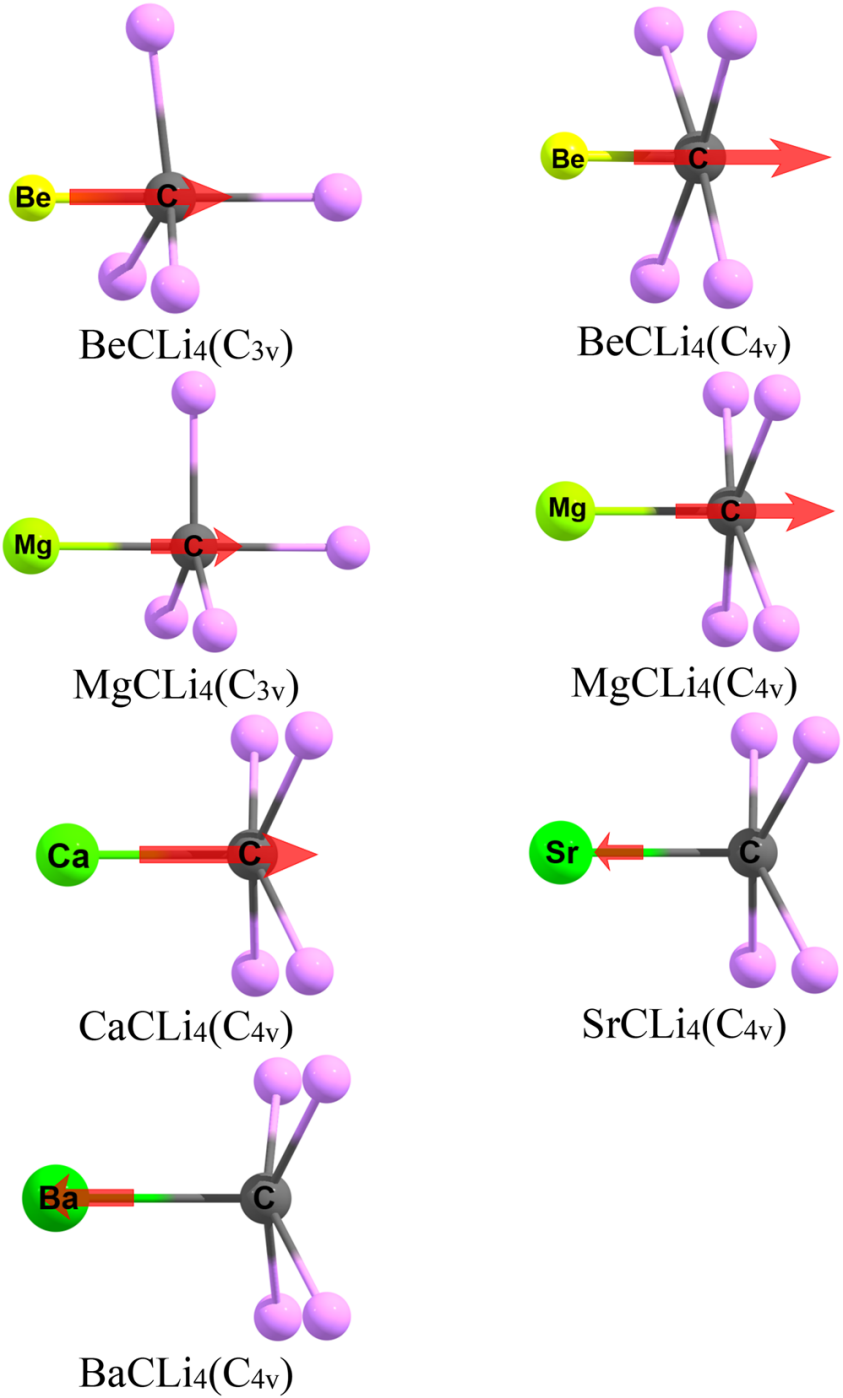
Orientation and direction of the dipole moment of AeCLi_4_ given by the red arrow. For the numerical values see Table 5.

Our previous study of the related complexes AeOLi_2_ showed that the molecules have large dipole moments between −8.18 D (Ae = Mg) and −3.78 D (Ae = Ba) calculated at CCSD(T)/def2‐QZVPP where the negative end is at the Ae atom with the polarity Ae → OLi_2_. Table 5 gives the calculated dipole moments of AeCLi_4_. The absolute values at the CCSD(T)/def2‐QZVPP level are bigger than at BP86‐D3(BJ)/def2‐QZVPP, but the trend at both levels of theory is the same. Large negative values of decreasing magnitude for the heavier systems with the negative end at the Ae atom are computed for Ae = Be, Mg, Ca. The direction of the dipole moments in these systems is Ae → CLi_4_. The direction of the dipole moment in the heavier system reverses and becomes Ae ← CLi_4_ when Ae = Sr. Ba. This might be due to the increase of the Ae‐CLi_4_ distance and a concomitant change in the polarization of the valence orbitals. Figure 11 displays the orientation and direction of the dipole moments.

**TABLE 5:** Calculated dipole moments *(Debye*) of AeCLi_4_ (Ae = Be, Mg, Ca, Sr. Ba) at two levels of theory using def2‐QZVPP basis sets.

Molecules	Dipole moment		
	BP86‐D3(BJ)	CCSD(T)	Negative End
BeCLi_4_(C_3v_)	−4.93	−8.41	Be
BeCLi_4_(C_4v_)	−3.14	−4.77	Be
MgCLi_4_(C_3v_)	−3.51	−6.31	Mg
MgCLi_4_(C_4v_)	−2.18	−3.60	Mg
CaCLi_4_(C_4v_)	−0.55	−2.56	Ca
SrCLi_4_(C_4v_)	0.11	1.57	C
BaCLi_4_(C_4v_)	0.24	0.53	C

## 5 Summary and Conclusion

The results of this work can be summarized as follows:

1. The calculated equilibrium geometries of the complexes AeCLi_4_ isomers of the lighter species with Ae = Be, Mg have a trigonal bipyramidal geometry (*C*_3*v*_ symmetry) as the energetically lowest‐lying form. There is a slightly higher‐lying isomer, which has a square pyramidal geometry (*C*_4*v*_ symmetry), which is only < 1 kcal/mol higher in energy than the *C*_3*v*_ isomer. In contrast, only the square pyramidal structure (*C*_4*v*_ symmetry) is an energy minimum for the heavier homologues with Ae = Ca, Sr, Ba.
2. The calculated BDEs of the Ae‐CLi_4_ bond are very high. The strongest bond is computed for the Be‐CLi_4_ bond, which has a BDE of *D*_e_ = 82.9 kcal/mol at the CCSD(T)/def2‐QZVPP level. The weakest bond is calculated for the Mg‐CLi_4_ bond, which has *D*_e_ = 40.6 kcal/mol. The heavier homologues have BDE values between *D*_e_ = 63.1 kcal/mol (Sr‐CLi_4_) and *D*_e_ = 72.0 kcal/mol (Ba‐CLi_4_). The BDE values at the BP86‐D3(BJ)/def2‐QZVPP level are a bit larger than the CCSD(T), but they show the same order.
3. Inspection of the occupied valence orbitals and the AdNDP results suggests that there are four Ae‐CLi_4_ bonds in the complexes. This is supported by the EDA‐NOCV analysis, which reveals that there is a dominant Ae → CLi_4_ σ‐donation, which resembles the collective interactions proposed by Martin‐Pendas and Foroutan‐Nejad. The Ae → CLi_4_ σ‐donation is enhanced by weaker Ae ← CLi_4_ σ‐backdonation along with degenerate Ae ← CLi_4_ π‐backdonation. The best signature of the chemical bonds is Ae 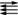 CLi_4_.
4. Inspection of the atomic orbitals of Ae atoms shows that the lighter atoms Be, Mg use their (n)s and (n)p AOs for the chemical bonds, whereas the heavier atoms Ca, Sr. Ba employ their (n)s and (n‐1)d AOs for the covalent interactions. The NBO method does not provide a reasonable account of the covalent bonds because it does not consider the 2p AOs of Li and the (n)p and (n‐1)d AOs of Ae atoms as genuine valence orbitals.
5. The QTAIM analysis of the chemical bonds, which is based on the topological analysis of the electronic structure, fails to account for the physical nature of the interatomic interaction resulting from the interference of the wave functions. In particular, the position of the bcp in polar bonds does not accurately reflect the nature of the chemical bond.

## Conflicts of Interest

The authors declare no conflicts of interest.

## Supporting information


**Table S1:** Computed the electronic energies (ΔE) of molecules using BP86‐D3(BJ)/def2‐QZVPP level. All energetic values are given in kcal/mol.
**Figure S1:** Plot of the transition state from C_3v_ to C_4v_ for singlet BeCLi_4_ and MgCLi_4_ calculated at the BP86‐D3(BJ)/def2‐QZVPP level.
**Table S2:** Computed IR frequencies and intensities (km mol^−1^) for AeCLi_4_ ν (stretching) and δ (bending).
**Table S3:** Coordinates of singlet AeCLi_4_ calculated at BP86‐D3(BJ)/def2‐QZVPP.
**Table S4:** Coordinates of singlet AeCLi_4_ calculated at CCSD(T)/def2‐QZVPP.
**Table S5:** Coordinates of the transition state from C_3v_ to C_4v_ for singlet BeCLi_4_ and MgCLi_4_ calculated at the BP86‐D3(BJ)/def2‐QZVPP level.
**Table S6:** Coordinates of singlet AeCLi4 calculated at HF/def2‐QZVPP.

## Data Availability

The data that supports the findings of this study are available in the Supporting Information of this article.
