# Supplementary material for: Quadruple Bonding of Alkaline Earth Atoms in AeCLi4 (Ae = Be − Ba) Complexes
Source: J Comput Chem. 2026 Jul 3;47(18):e70449. doi: 10.1002/jcc.70449 (PMC13332347; doi:10.1002/jcc.70449)
Supplement: Supplementary file 1 — Table S1: Computed the electronic energies (ΔE) of molecules using BP86‐D3(BJ)/def2‐QZVPP level. All energetic values are given in kcal/mol. Figure S1: Plot of the transition state from C3v to C4v for singlet BeCLi4 and MgCLi4 calculated at the BP86‐D3(BJ)/def2‐QZVPP level. Table S2: Computed IR frequencies and intensities (km mol−1) for AeCLi4 ν (stretching) and δ (bending). Table S3: Coordinates of singlet AeCLi4 calculated at BP86‐D3(BJ)/def2‐QZVPP. Table S4: Coordinates of singlet AeCLi4 calculated at CCSD(T)/def2‐QZVPP. Table S5: Coordinates of the transition state from C3v to C4v for singlet BeCLi4 and MgCLi4 calculated at the BP86‐D3(BJ)/def2‐QZVPP level. Table S6: Coordinates of singlet AeCLi4 calculated at HF/def2‐QZVPP. [file JCC-47-0-s001.docx]

**Supporting Information for**

Quadruple Bonding of Alkaline Earth Atoms in AeCLi_4_ (Ae = Be - Ba) Complexes

Yahui Li, Chengxiang Ding, Sudip Pan and Gernot Frenking

**Table S1.** Computed the electronic energies (ΔE) of molecules using BP86-D3(BJ)/def2-QZVPP level. All energetic values are given in kcal/mol.

| AeCLi_4_ | | | | |
| --- | --- | --- | --- | --- |
| BP86+D3(BJ)  /def2-QZVPP | C_3v_ (Singlet)  ΔE [ΔE_0_] | C_3v_ (Triplet)  ΔE [ΔE_0_] | C_4v_ (Singlet)  ΔE [ΔE_0_] | C_4v_ (Triplet)  ΔE [ΔE_0_] |
| BeCLi_4_ | 0.0 [0.0] | 10.8 [10.1] | 0.1 [0.3] |  |
| MgCLi_4_ | 0.0 [0.0] | 13.9 [13.5] | 0.6 [0.7] | 14.2 [13.8] |
| CaCLi_4_ |  |  | 0.0 [0.0] | 16.9 [16.4] |
| SrCLi_4_ |  |  | 0.0 [0.0] | 17.3 [16.9] |
| BaCLi_4_ |  |  | 0.0 [0.0] | 15.0 [14.7] |

**Figure S1.** Plot of the transition state from C_3v_ to C_4v_ for singlet BeCLi_4_ and MgCLi_4_ calculated at the BP86-D3(BJ)/def2-QZVPP level.

**Table S2.** Computed IR frequencies and intensities (km mol^–1^) for AeCLi_4_ ν (stretching) and δ (bending).

(For specific Ae-C stretching)

| Bond |  | AeCLi_4_ | | |
| --- | --- | --- | --- | --- |
|  |  | BP86-D3(BJ) | CCSD (T) |  |
| Be-C (C_3v_) |  | 1016.3(7) | 1010.6 |  |
| Mg-C (C_3v_) |  | 620.4(1) | 626.4 |  |
| Ca-C |  | 550.3(17) | 520.9 |  |
| Sr-C |  | 415.2(13) | 393.8 |  |
| Ba-C |  | 400.2(19) | 373.8 |  |

For complete set at the BP86-D3(BJ) level

| 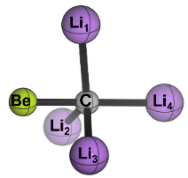  **BeCLi_4_ C_3v_** | | | |
| --- | --- | --- | --- |
| Assignment | Frequencies | Intensities |  |
| δ(Li-C-Li) | 72.1 | 6.2 | 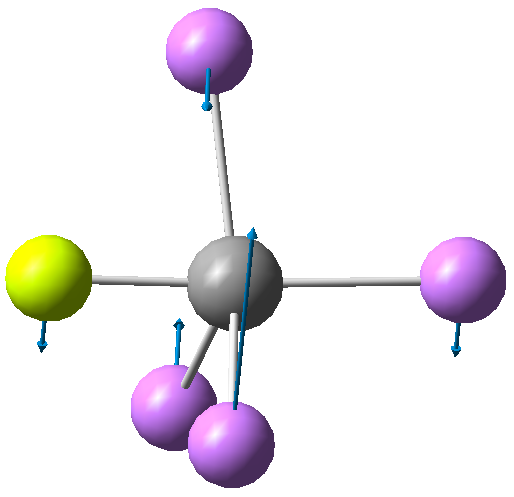 |
| δ(Li-C-Li) | 72.1 | 6.2 | 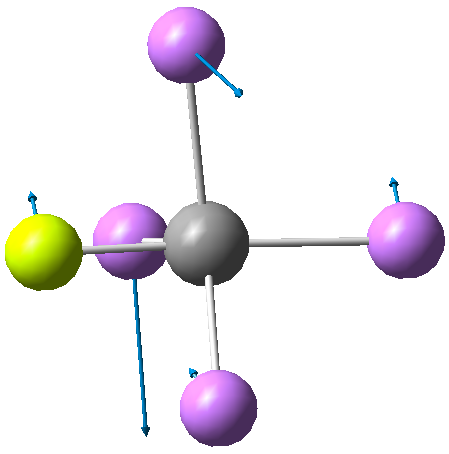 |
| δ(Li-C-Li) | 219.9 | 7.4 | 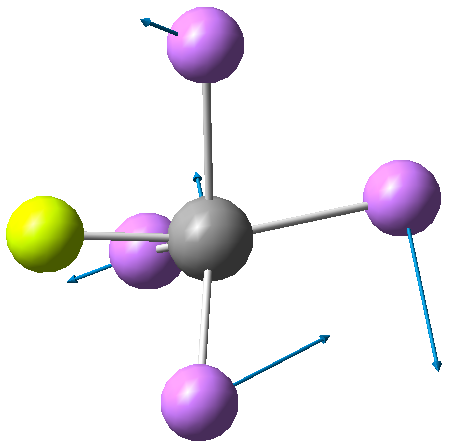 |
| δ(Li-C-Li) | 219.9 | 7.4 | 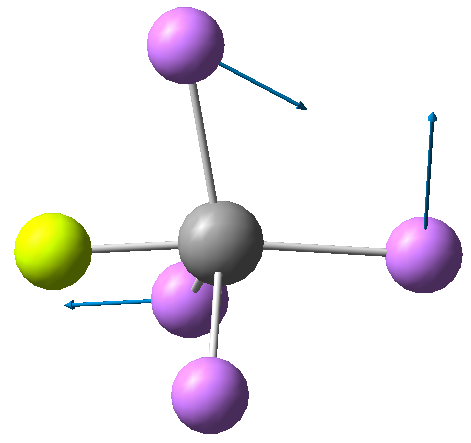 |
| δ(CLi_3_) | 261.9 | 17.2 | 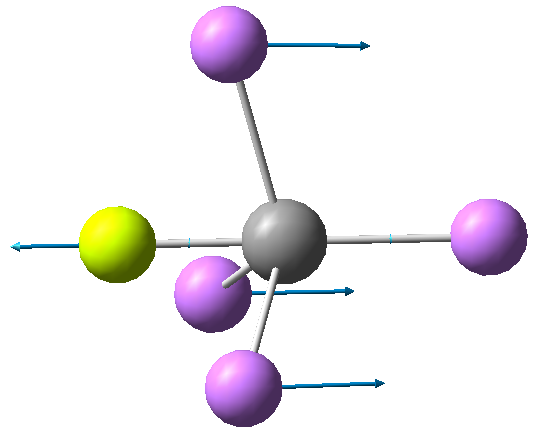 |
| δ(Be-C-Li) | 328.3 | 45.0 | 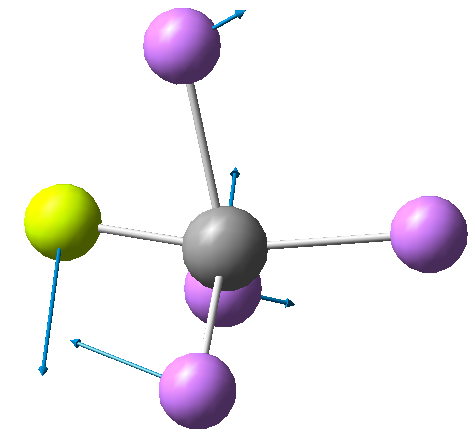 |
| δ(Be-C-Li) | 328.3 | 45.0 | 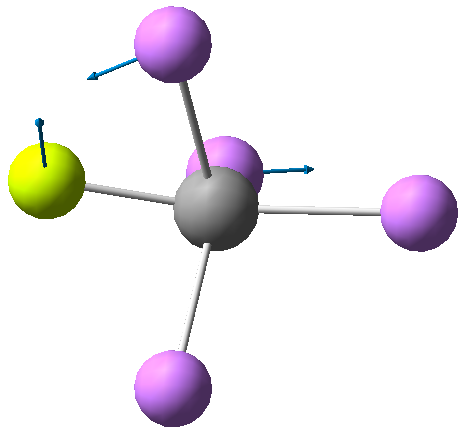 |
| ν(C-Li_3_) | 475.3 | 5.6 | 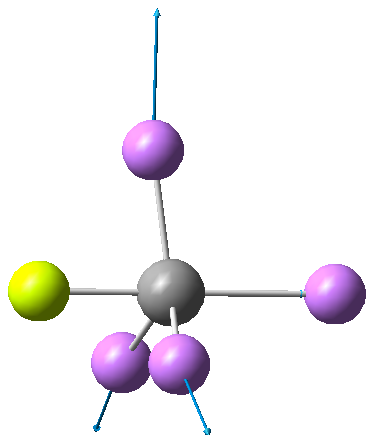 |
| ν(Be-C-Li) | 540.9 | 1.4 | 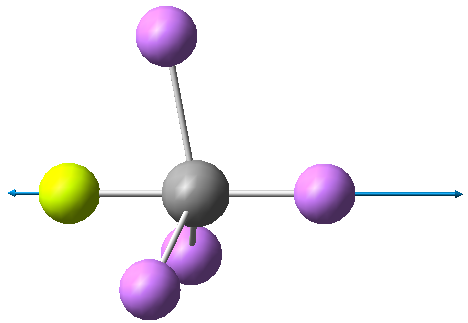 |
| ν(Li-C-Li) | 620.7 | 68.7 | 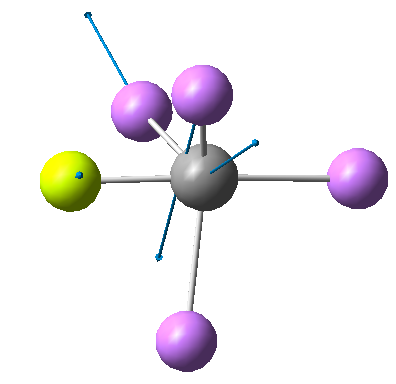 |
| ν(Li-C-Li) | 620.7 | 68.7 | 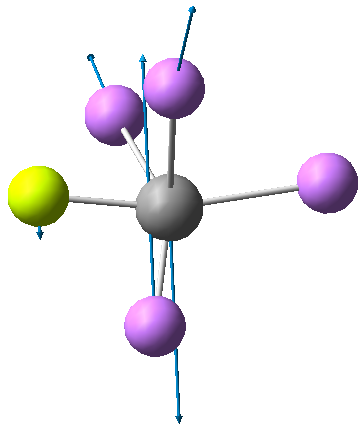 |
| ν(Li-Be-C) | 1016.3 | 6.6 | 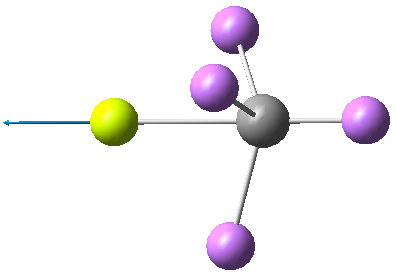 |
| 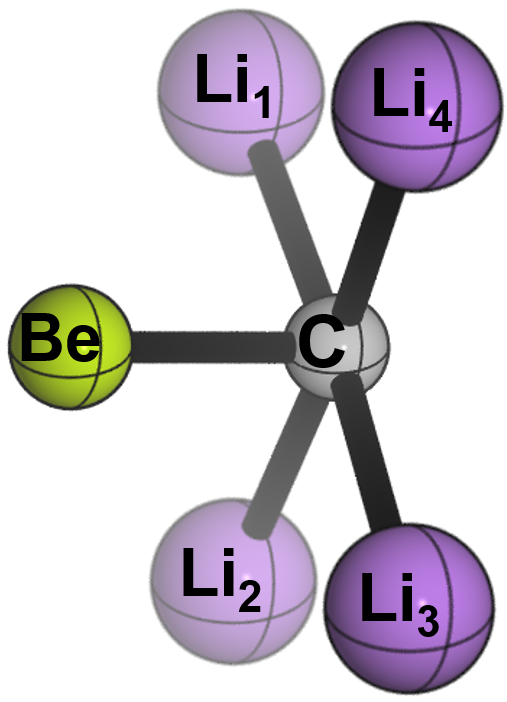  **BeCLi_4_ C_4_v** | | | |
| Assignment | Frequencies | Intensities |  |
| δ(CLi_4_) | 109.9 | 0.0 | 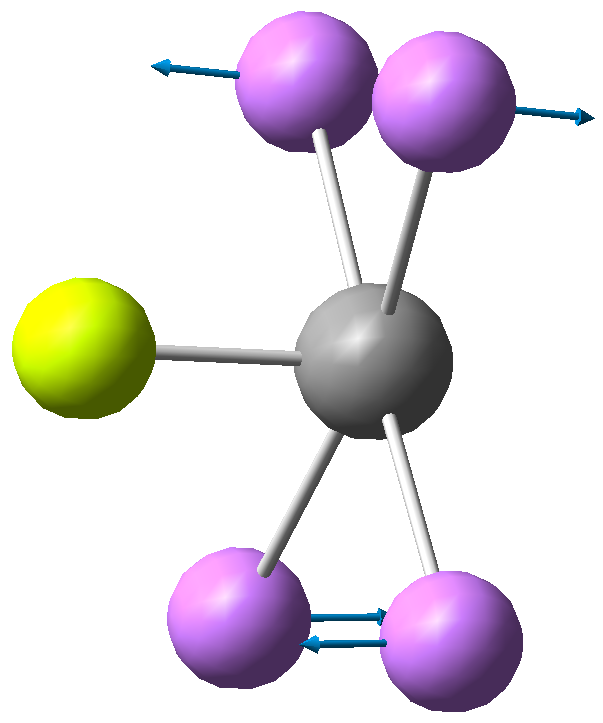 |
| δ(Li-C-Li) | 240.5 | 0.5 | 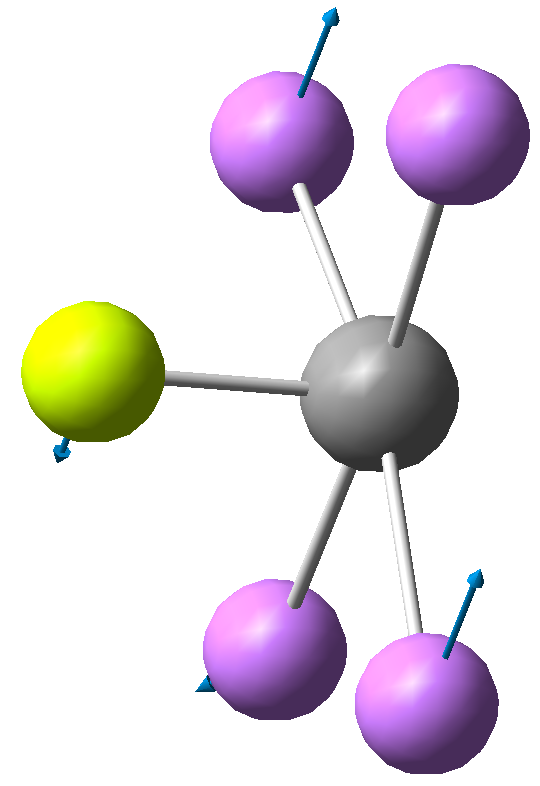 |
| δ(Li-C-Li) | 240.5 | 0.5 | 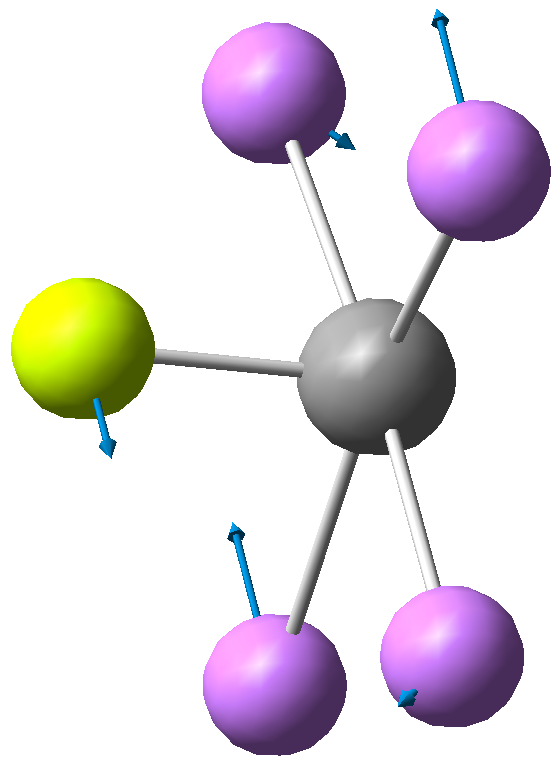 |
| δ(CLi_4_) | 243.8 | 33.3 | 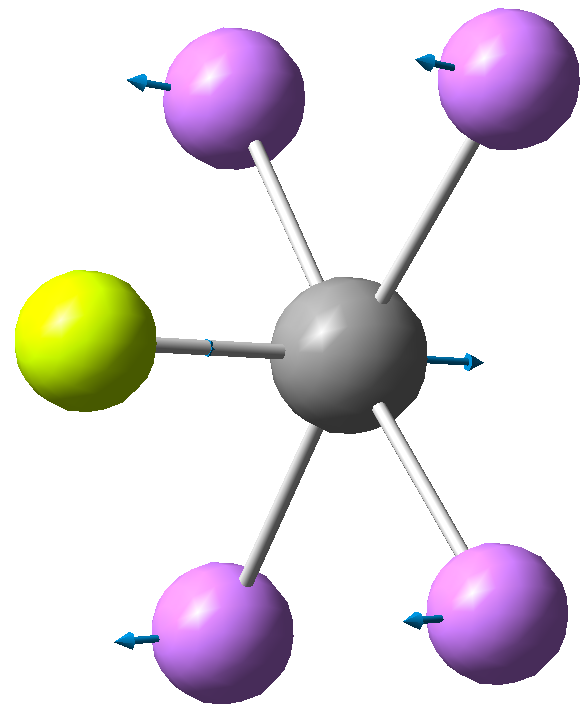 |
| δ(Be-C-Li) | 272.2 | 145.1 | 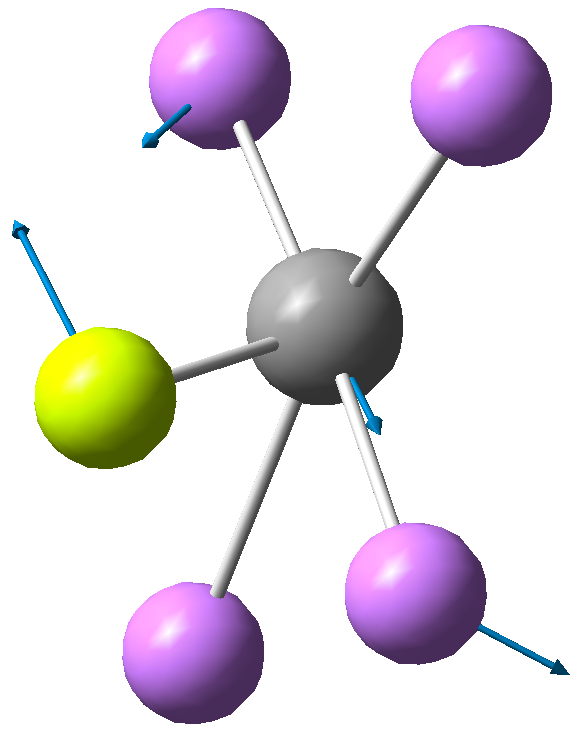 |
| δ(Be-C-Li) | 272.2 | 145.1 | 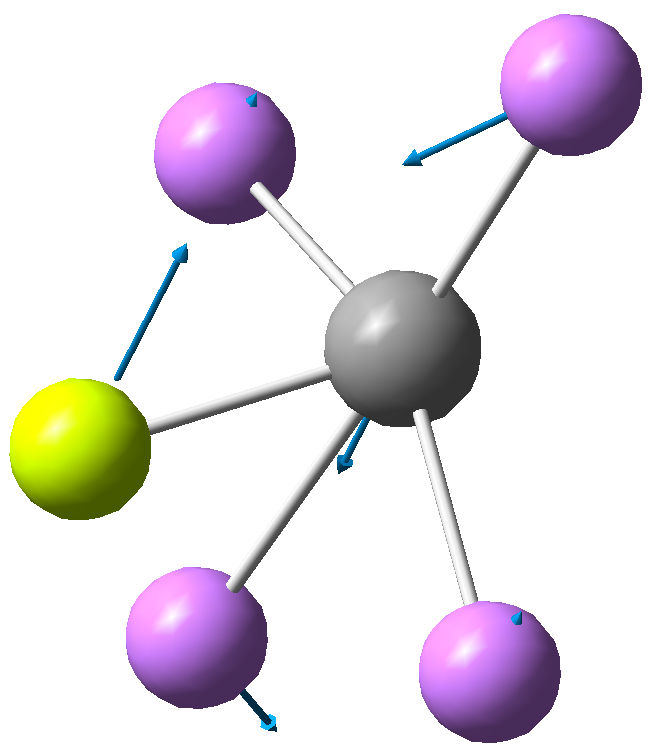 |
| δ(Li-C-Li) | 281.7 | 0.0 | 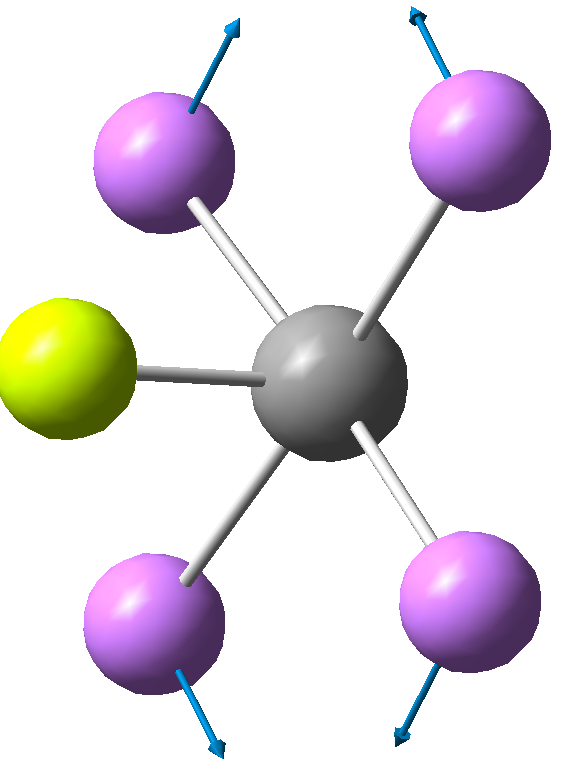 |
| ν(Li-C-Li) | 461.9 | 0.0 | 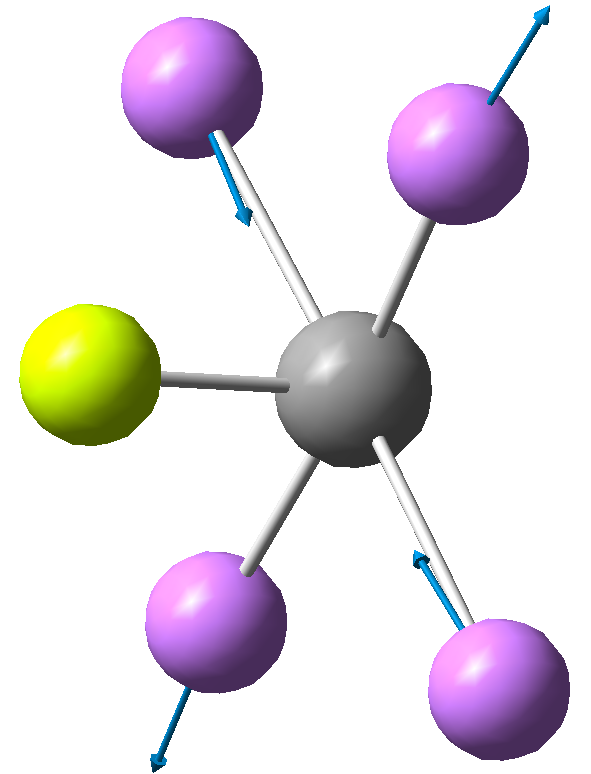 |
| ν(Li-C-Li) | 493.2 | 0.3 | 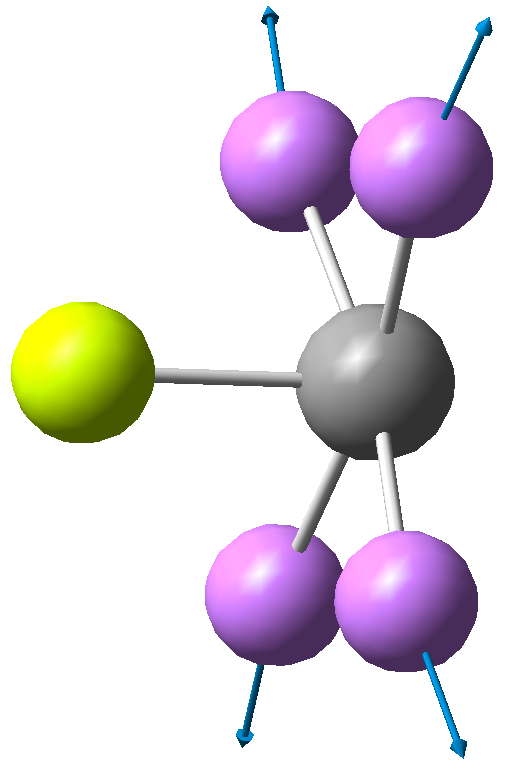 |
| ν(Li-C-Li) | 628.7 | 24.5 | 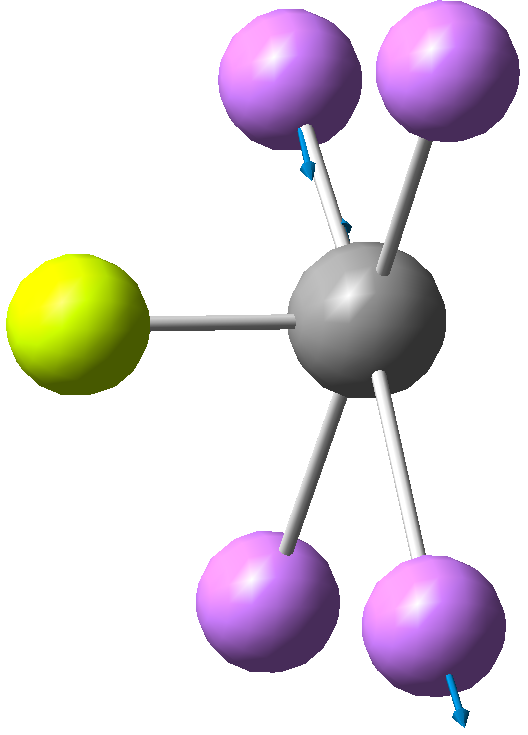 |
| ν(Li-C-Li) | 628.7 | 24.5 | 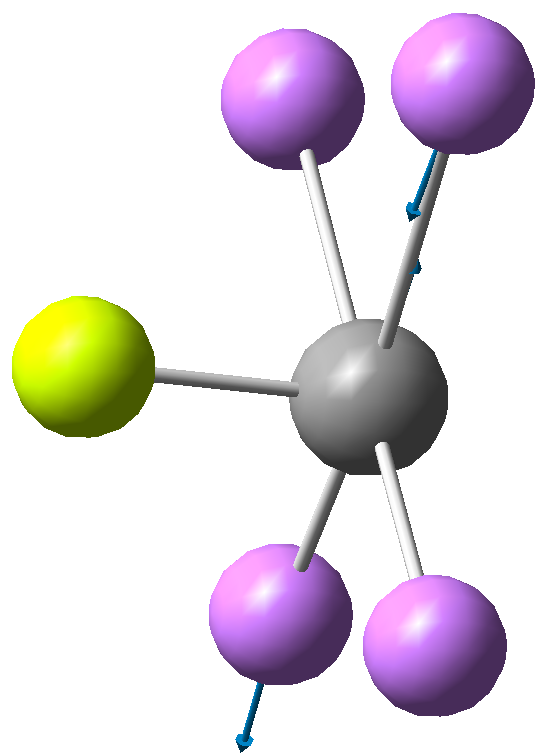 |
| ν(Be-C) | 1023.4 | 20.5 | 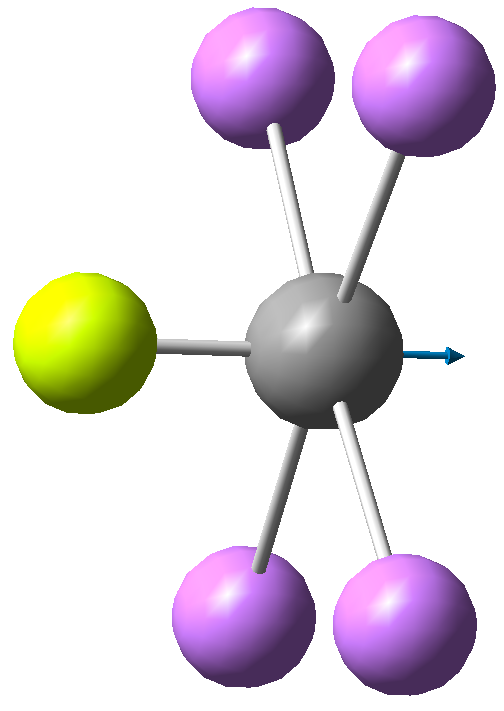 |
| 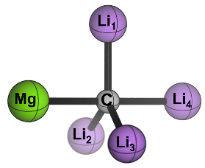  **MgCLi_4_ C_3v_** | | | |
| Assignment | Frequencies | Intensities |  |
| δ(Li-C-Li) | 51.7 | 4.3 | 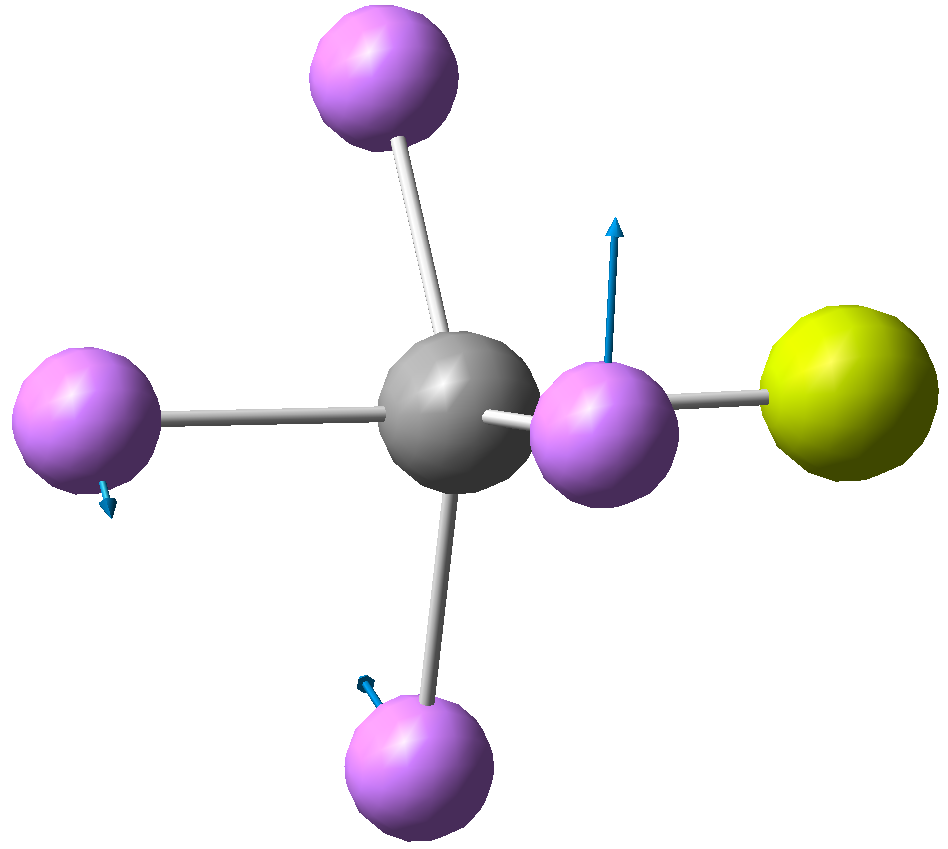 |
| δ(Li-C-Li) | 51.7 | 4.3 | 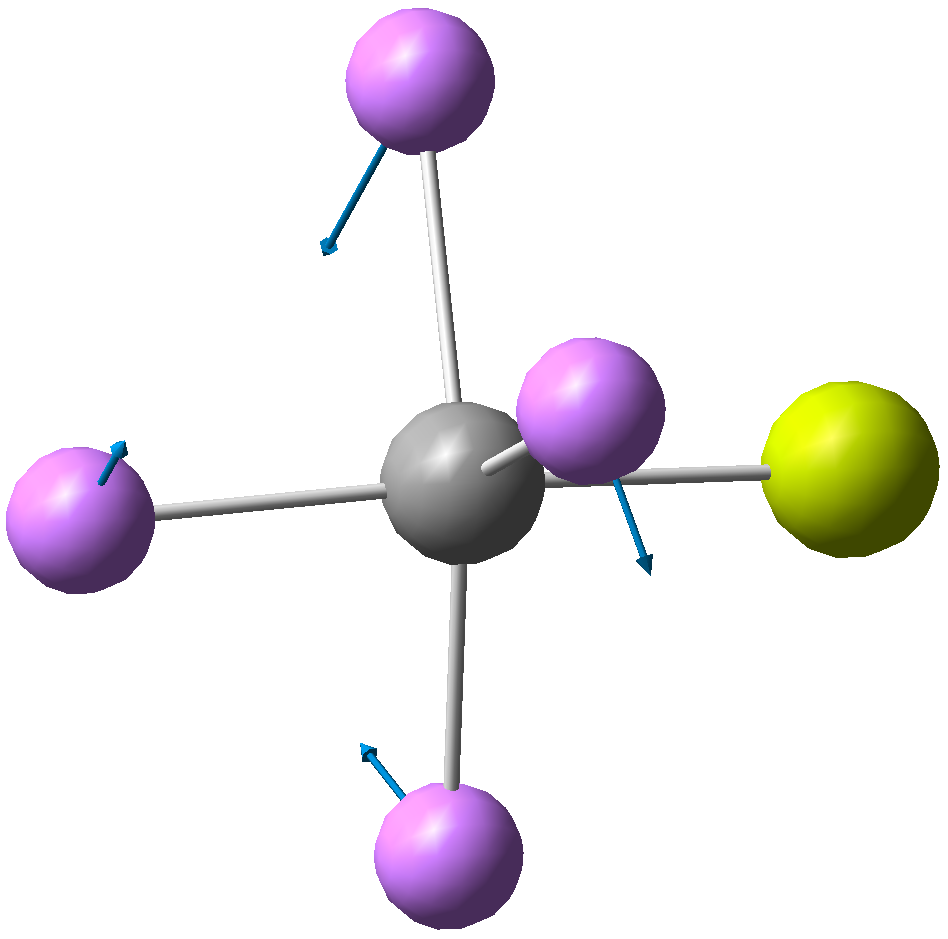 |
| δ(Li-C-Li-Mg) | 206.3 | 15.3 | 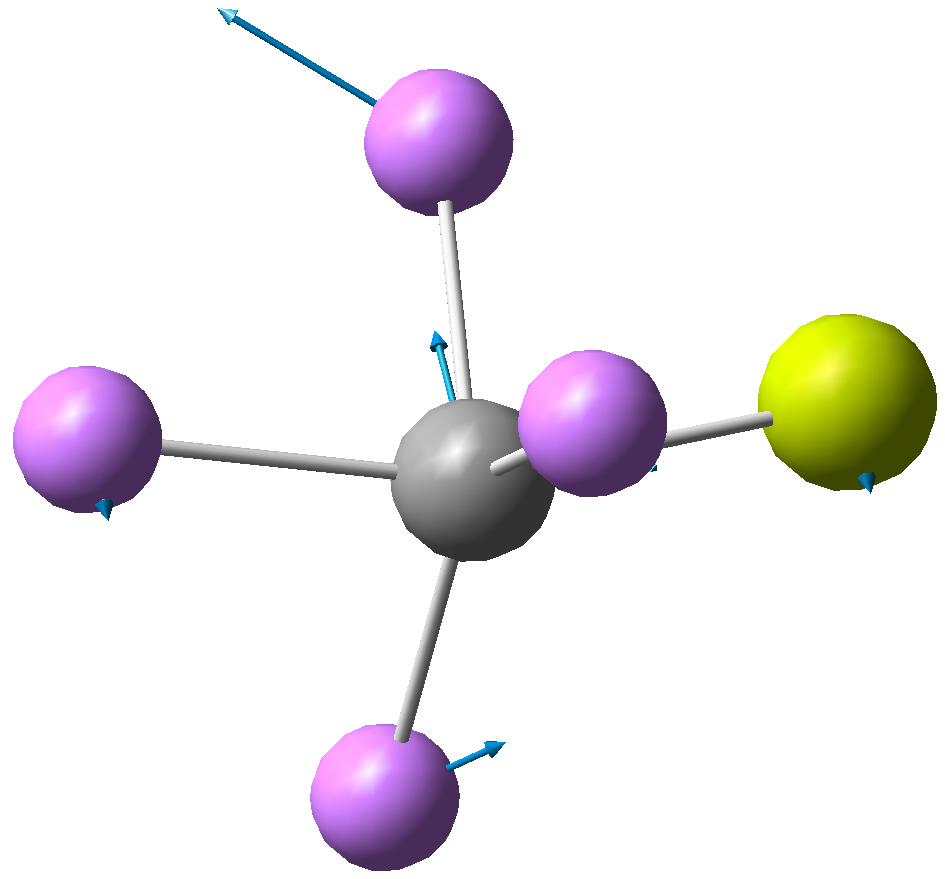 |
| δ(Li-C-Li-Mg) | 206.3 | 15.3 | 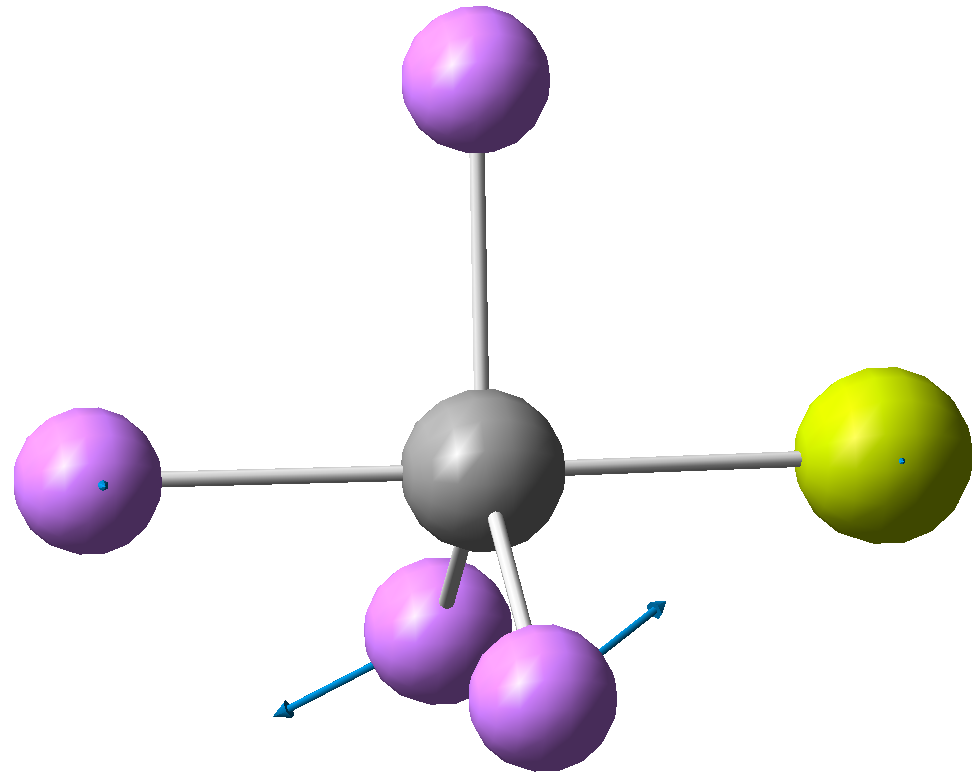 |
| δ(CLi_3_) | 248.3 | 5.4 | 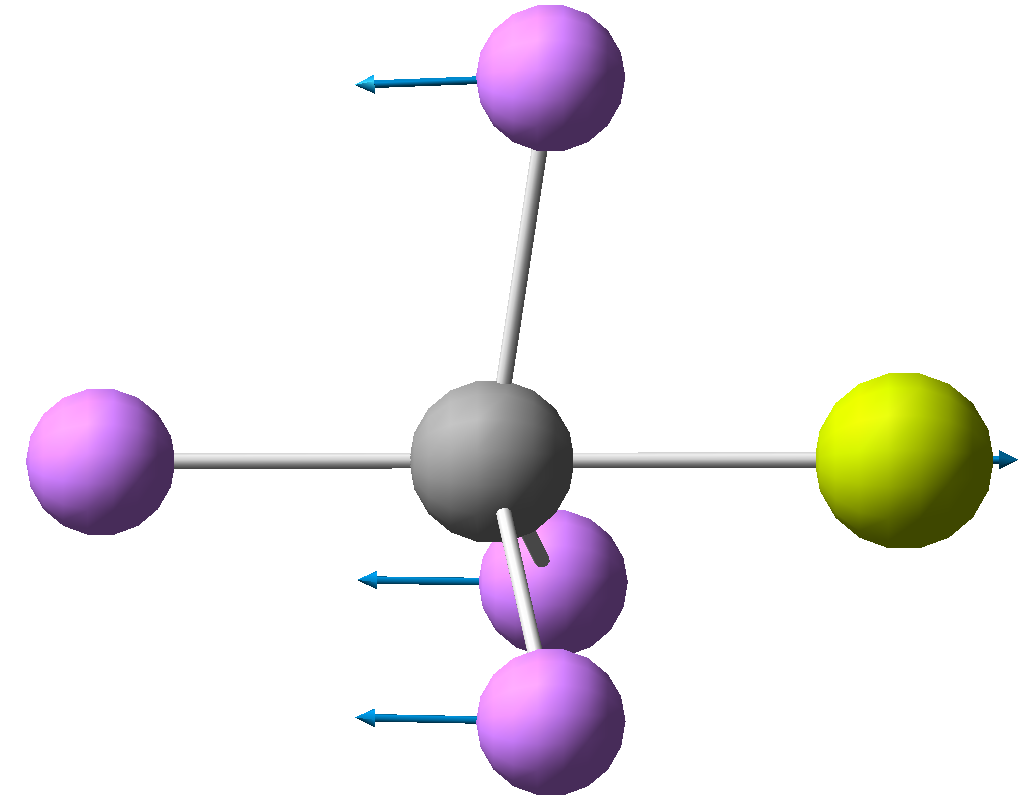 |
| δ(Li-C-Li) | 264.4 | 0.2 | 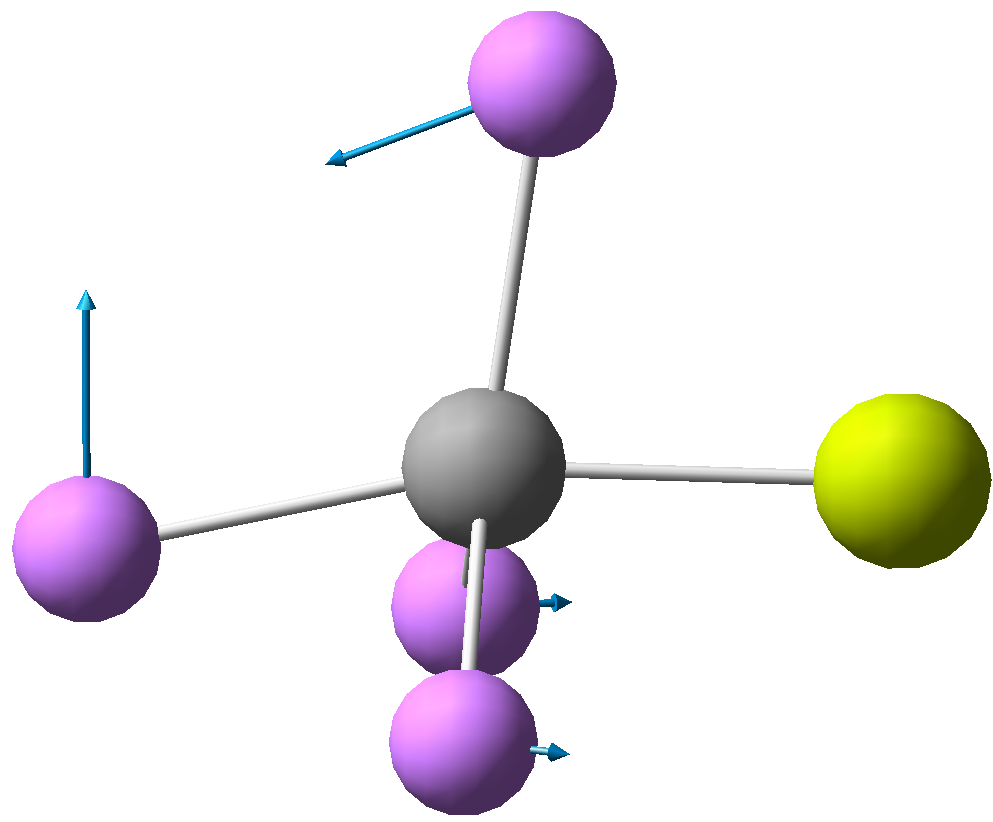 |
| δ(Li-C-Li) | 264.4 | 0.2 | 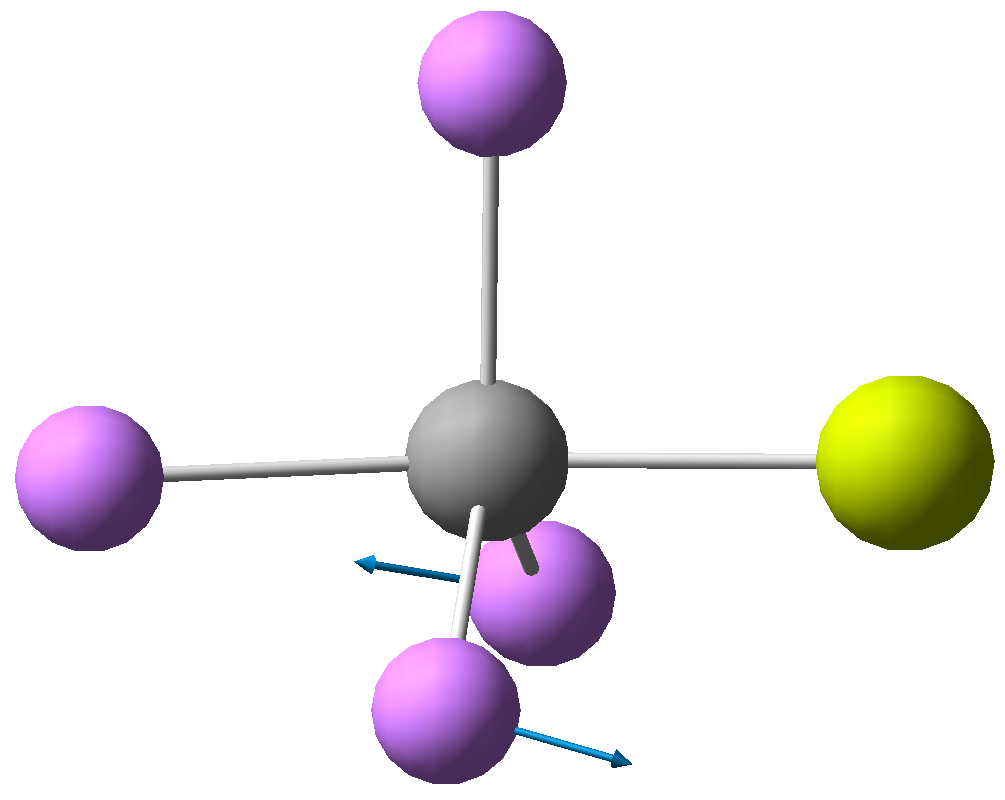 |
| ν(Li-C-Mg) | 388.1 | 14.2 | 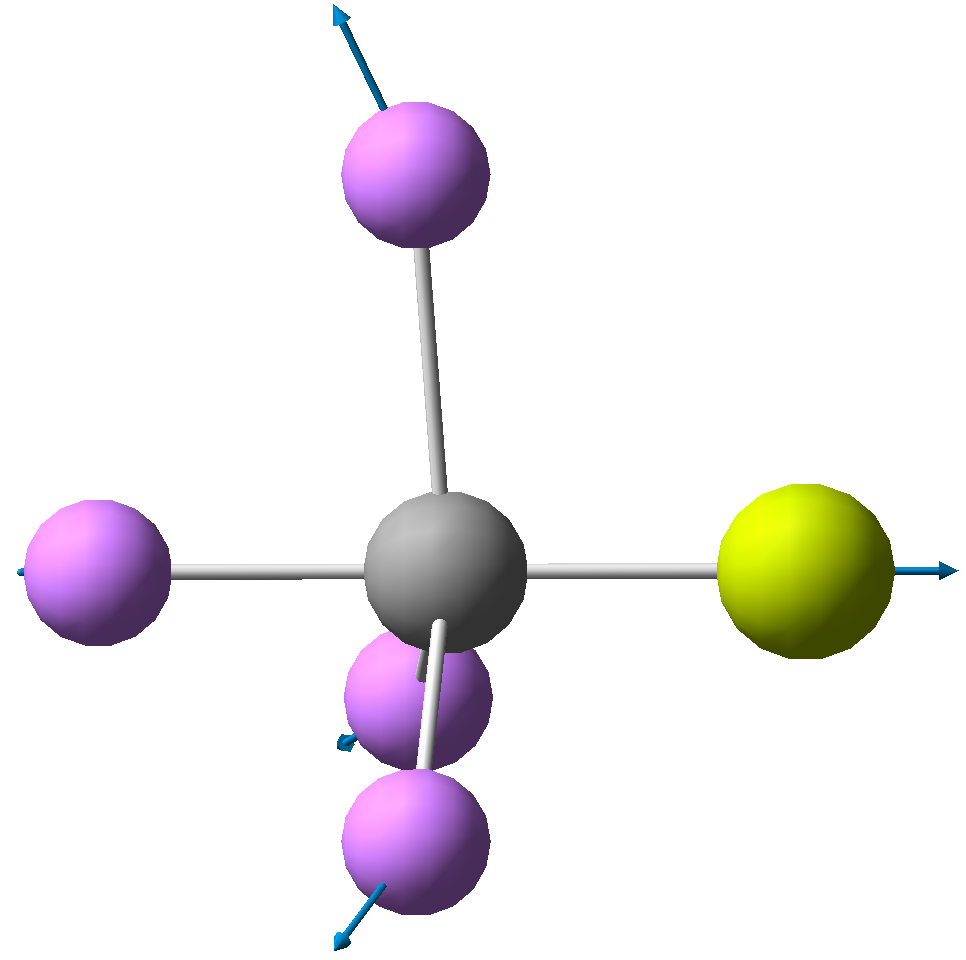 |
| ν(C-Li_3_) | 483.3 | 2.8 | 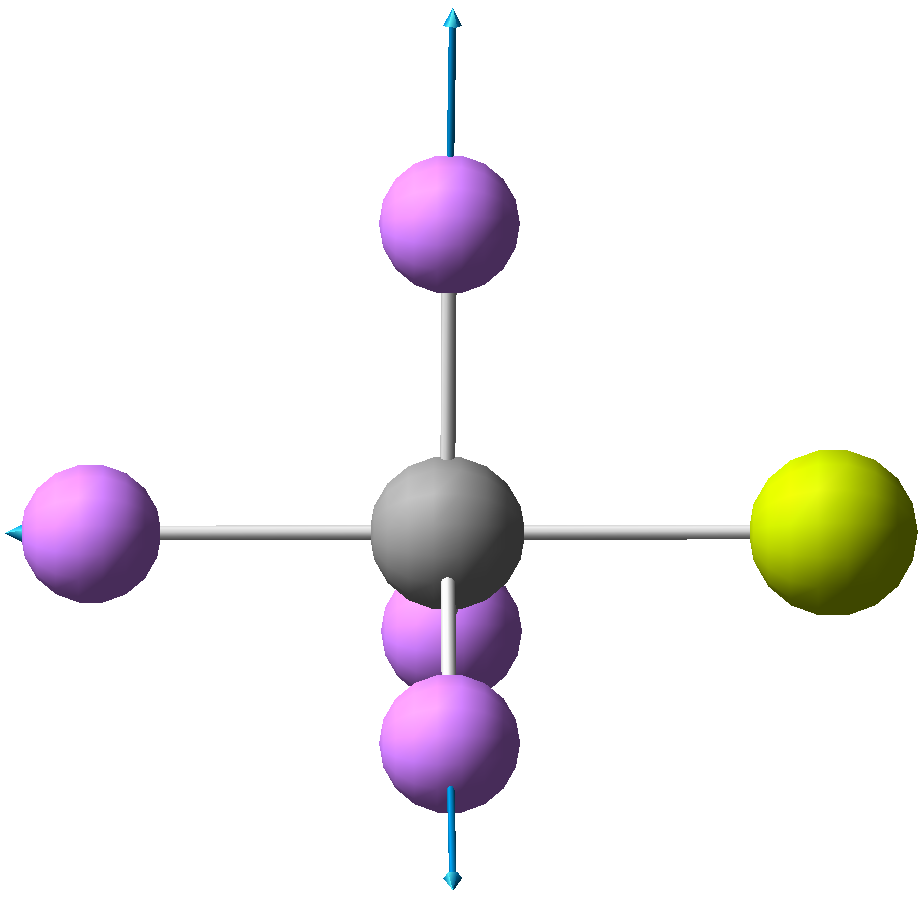 |
| ν(Mg-C-Li) | 620.4 | 1.4 | 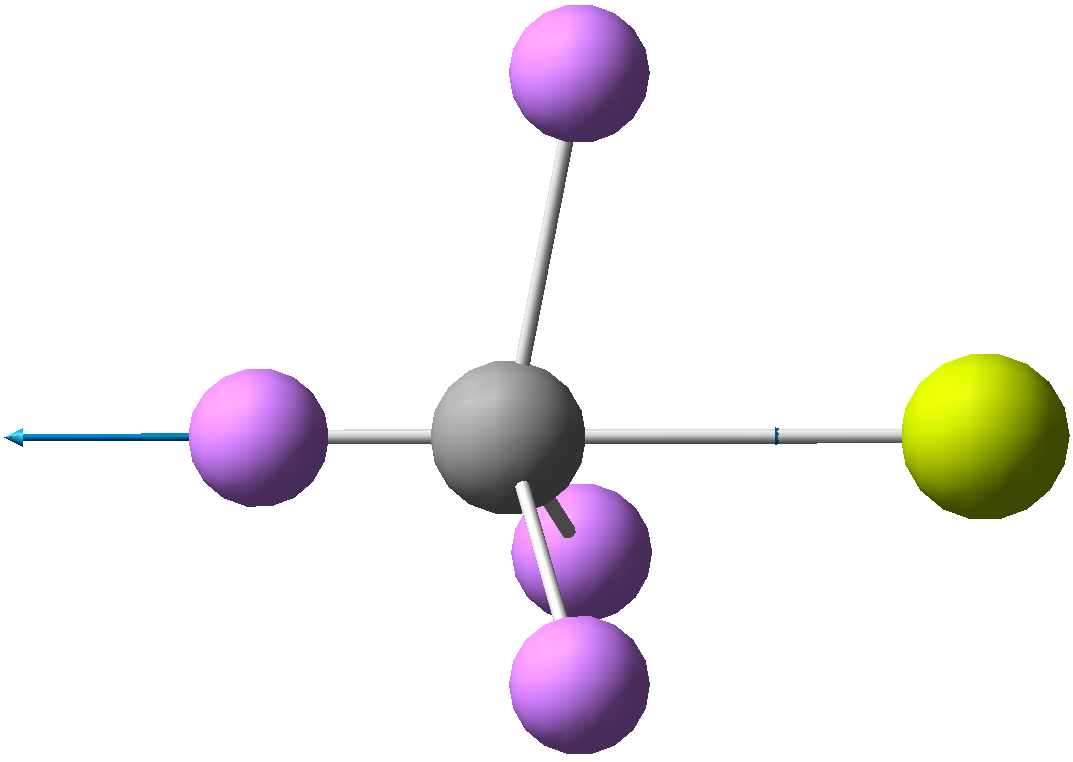 |
| ν(Li-C-Li) | 652.8 | 70.8 | 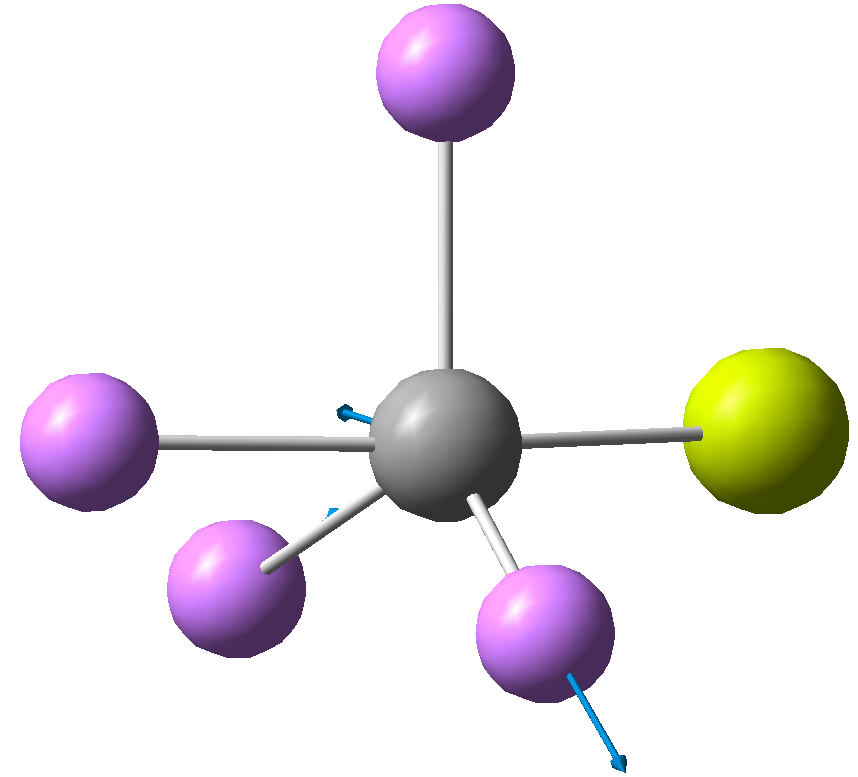 |
| ν(Li-C-Li) | 652.8 | 70.8 | 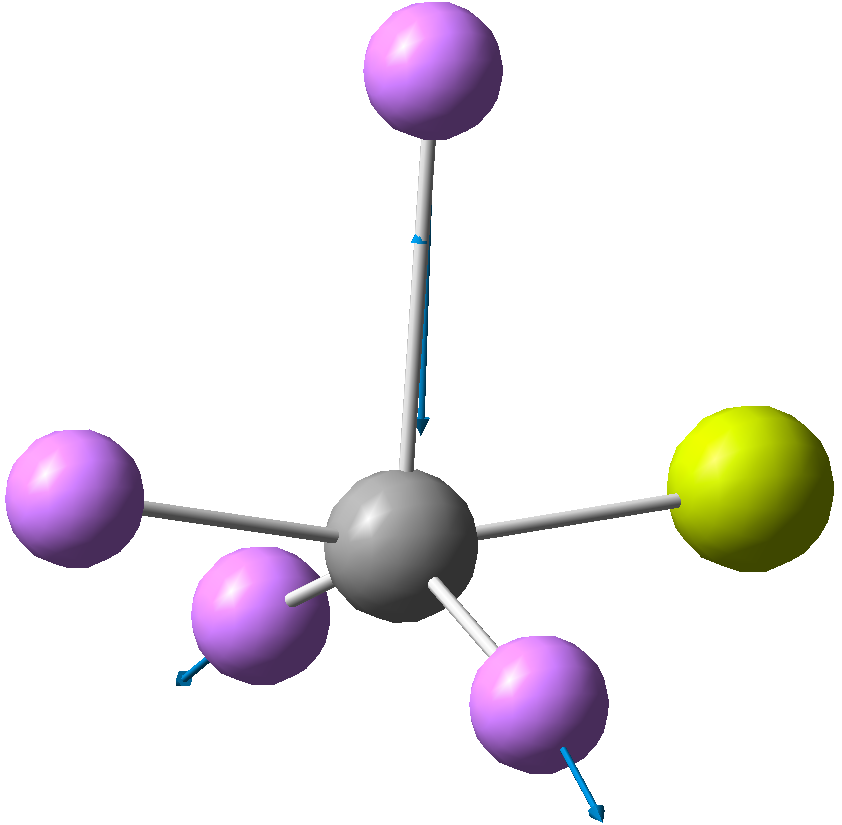 |
| 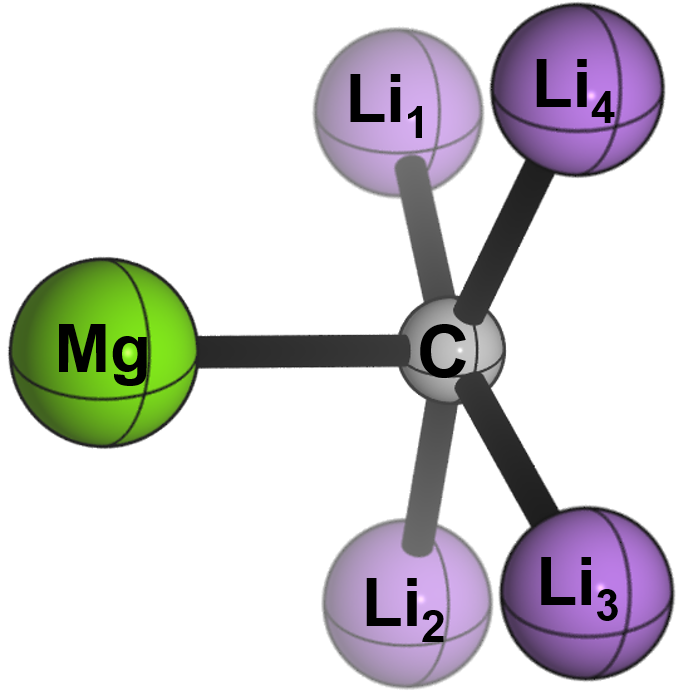  **MgCLi_4_ C_4v_** | | | |
| Assignment | Frequencies | Intensities |  |
| δ(Li-C-Li) | 49.1 | 0.0 | 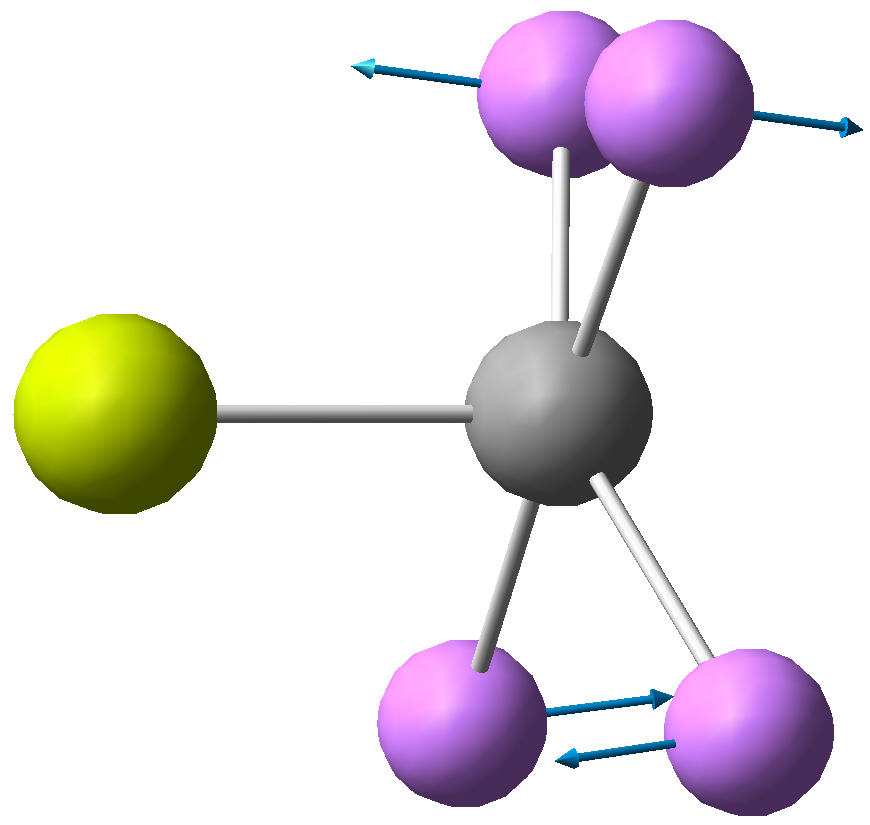 |
| δ(Li-C-Li-Mg) | 146.1 | 34.8 | 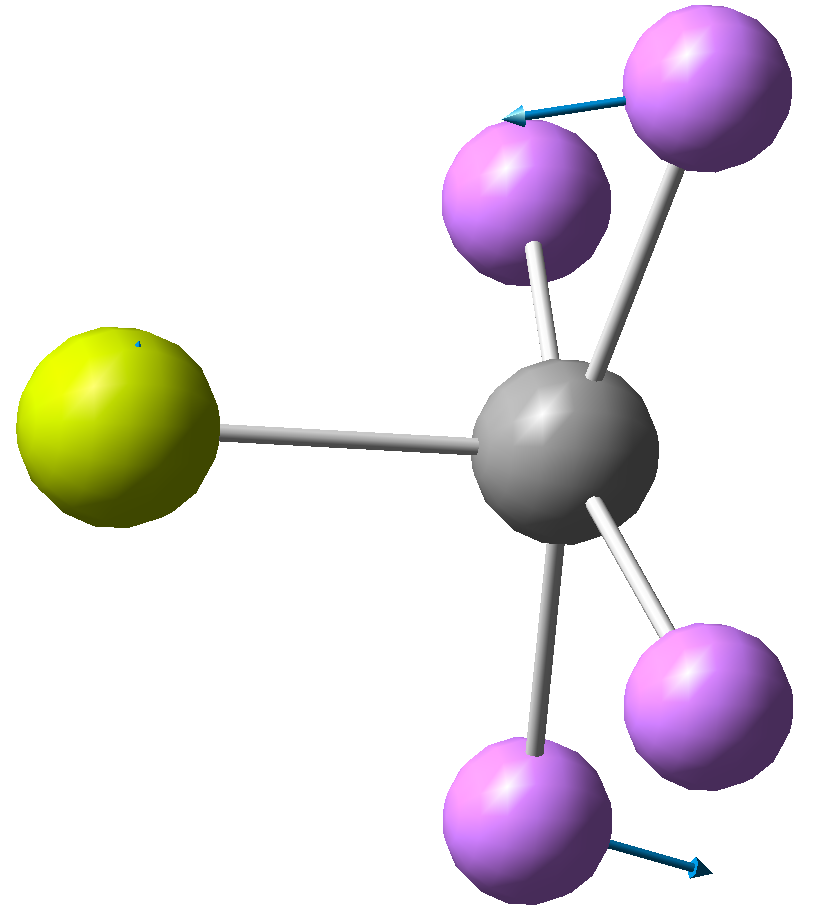 |
| δ(Li-C-Li-Mg) | 146.1 | 34.8 | 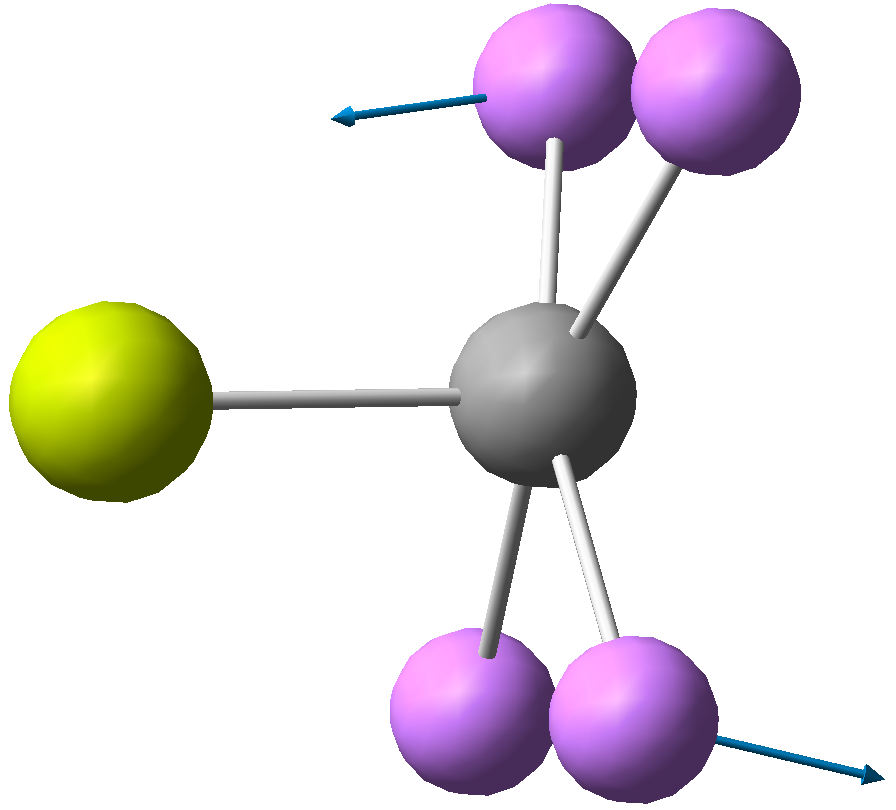 |
| δ(CLi_4_) | 195.9 | 14.7 | 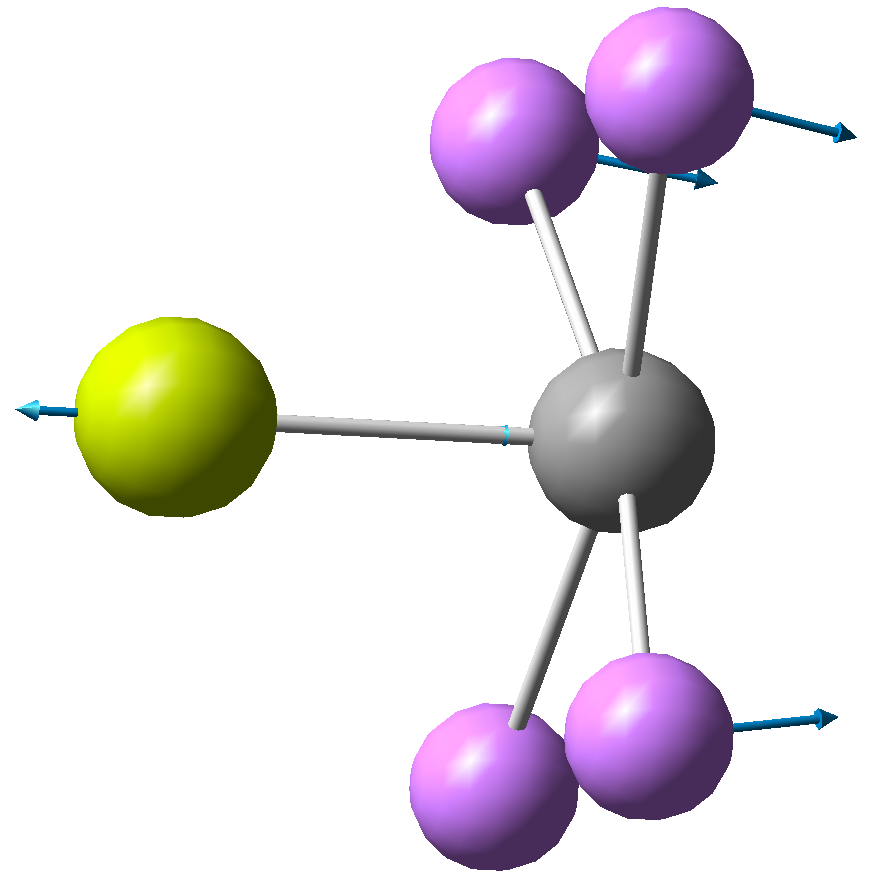 |
| δ(Li-C-Li) | 261.5 | 38.9 | 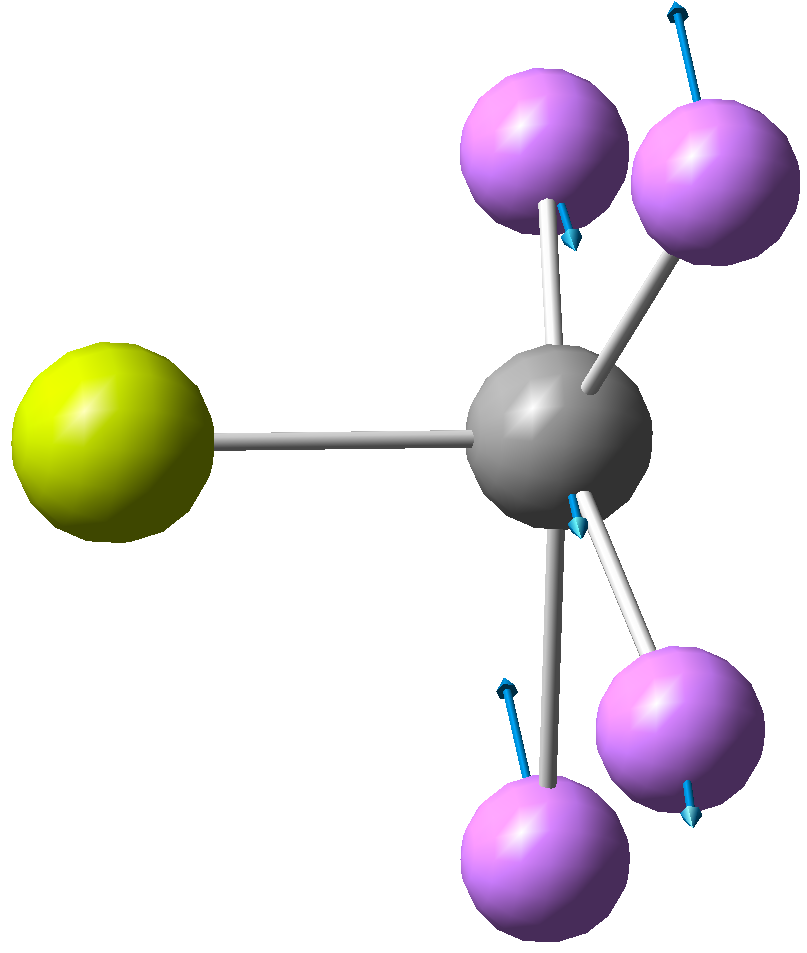 |
| δ(Li-C-Li) | 261.5 | 38.9 | 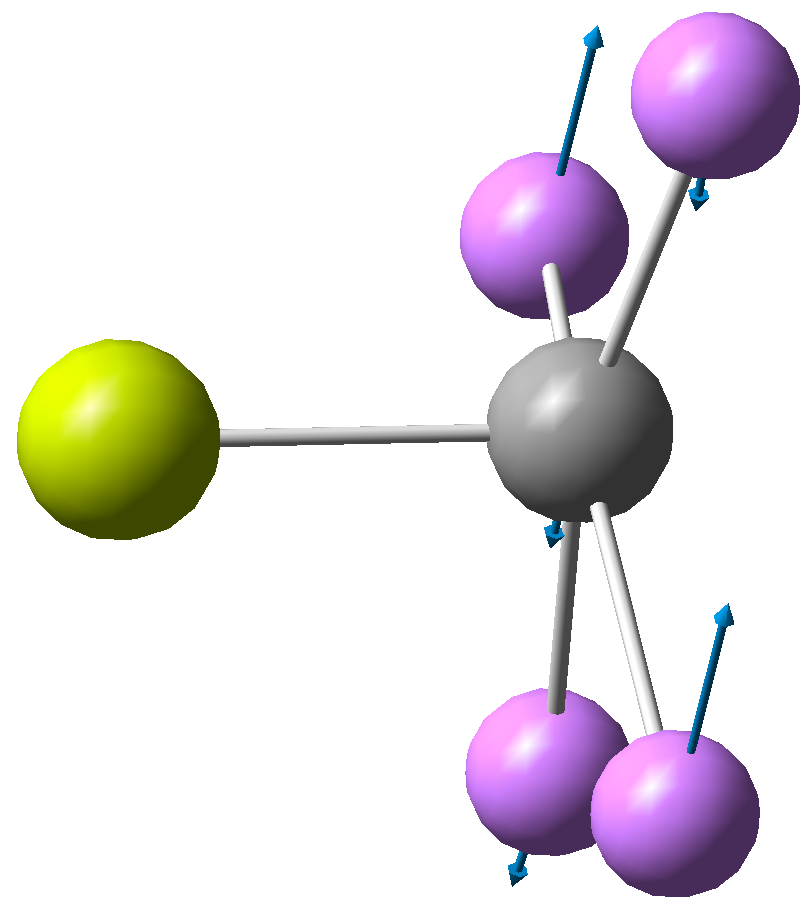 |
| δ(Li-C-Li) | 296.6 | 0.0 | 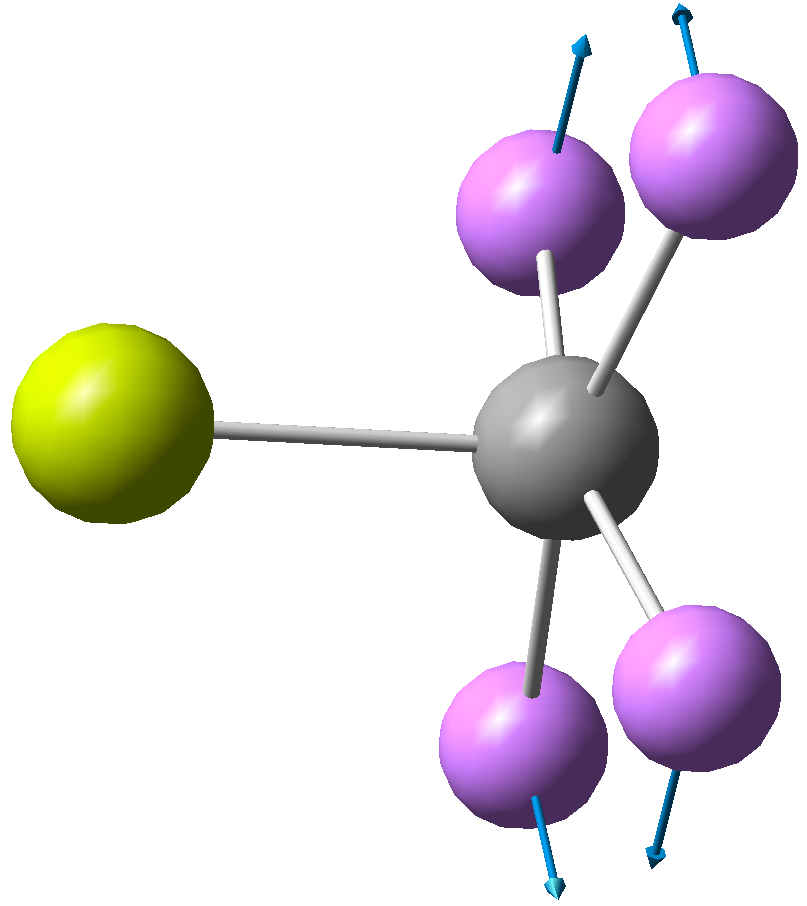 |
| ν(Li-C) | 462.1 | 0.0 | 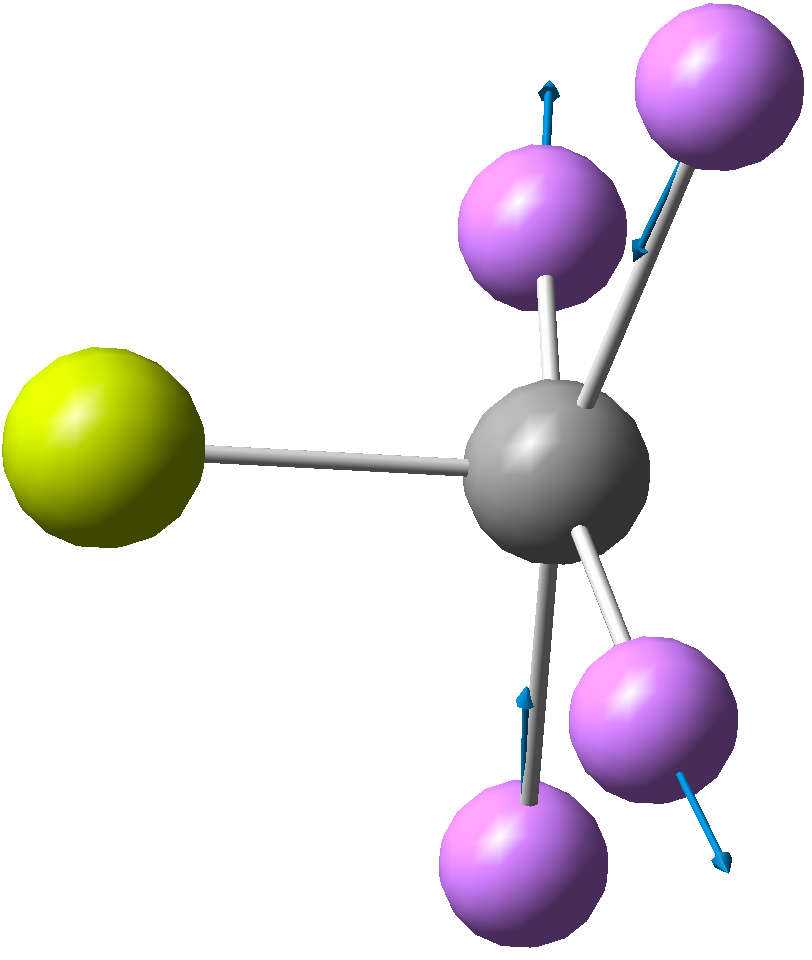 |
| ν(Li-C) | 481.8 | 3.7 | 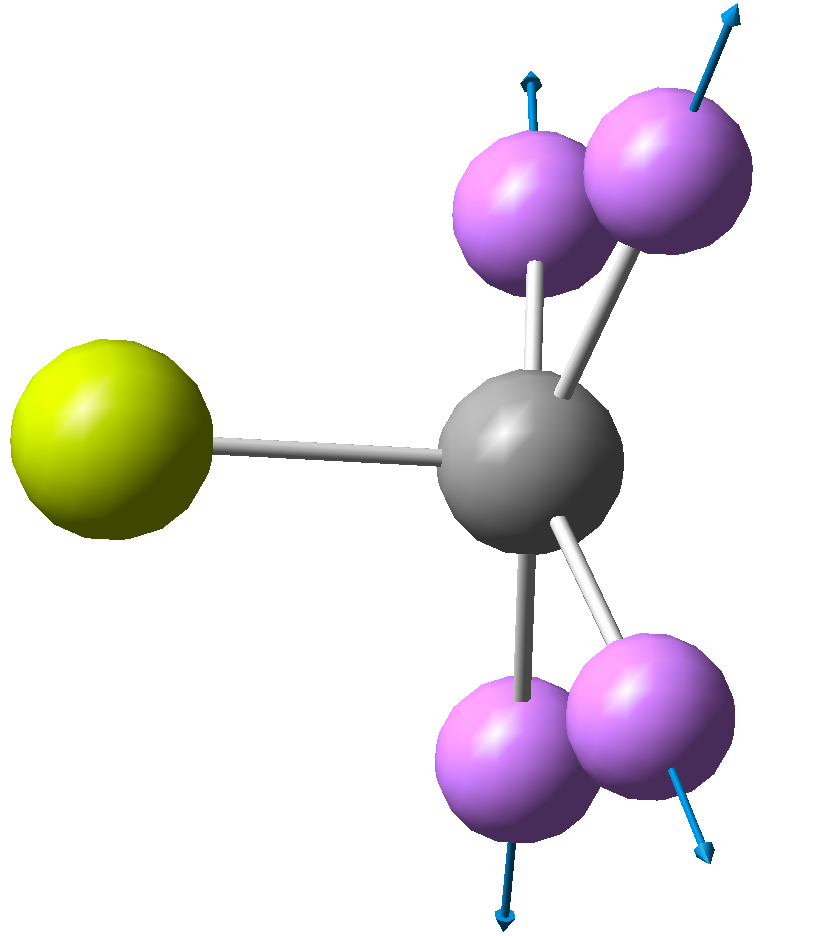 |
| ν(C-Mg) | 561.7 | 2.8 | 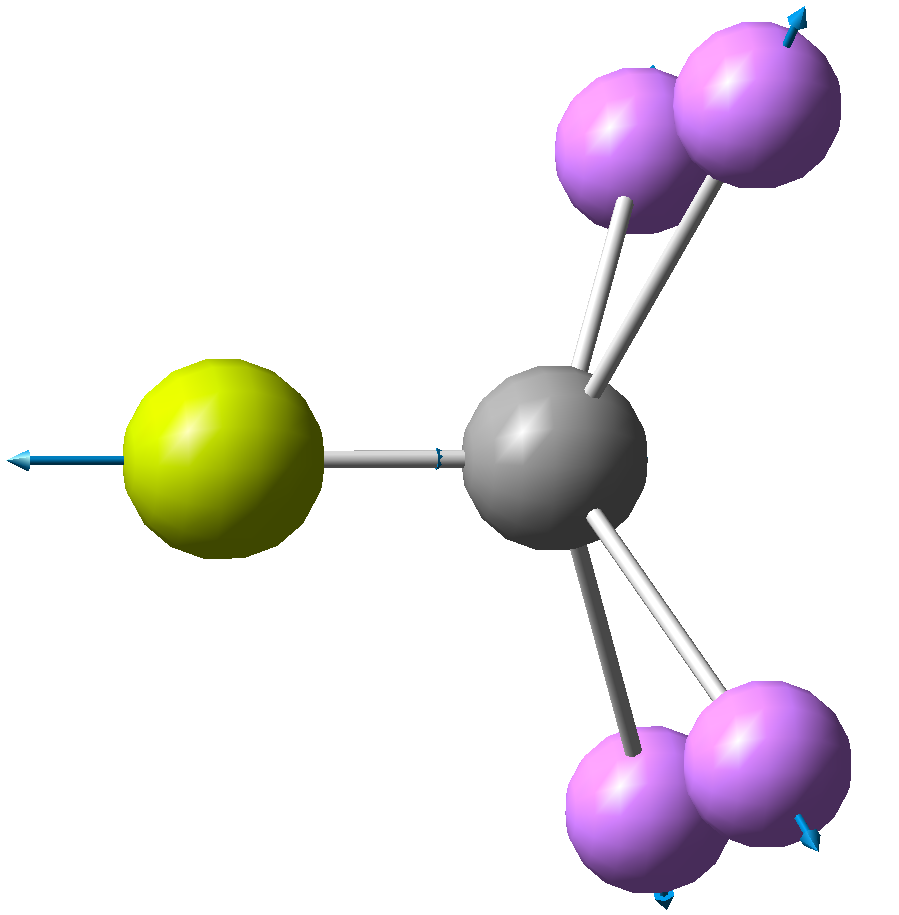 |
| ν(Li-C-Li) | 653.6 | 22.9 | 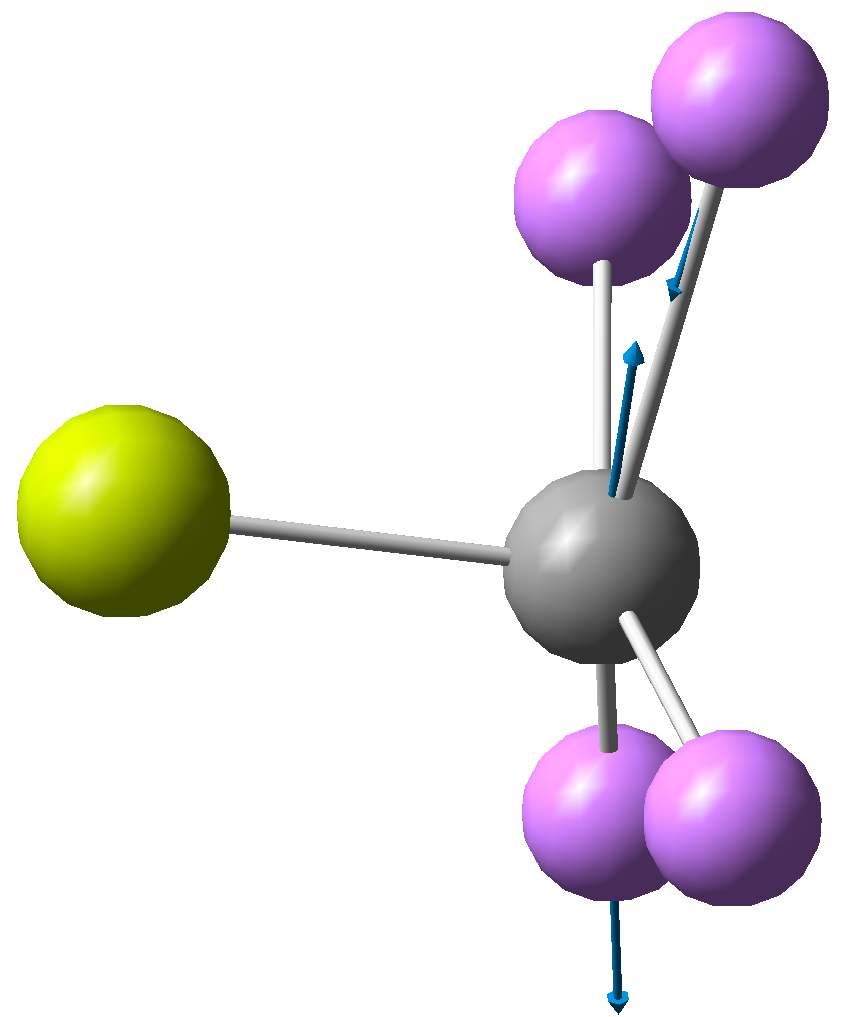 |
| ν(Li-C-Li) | 653.6 | 22.9 | 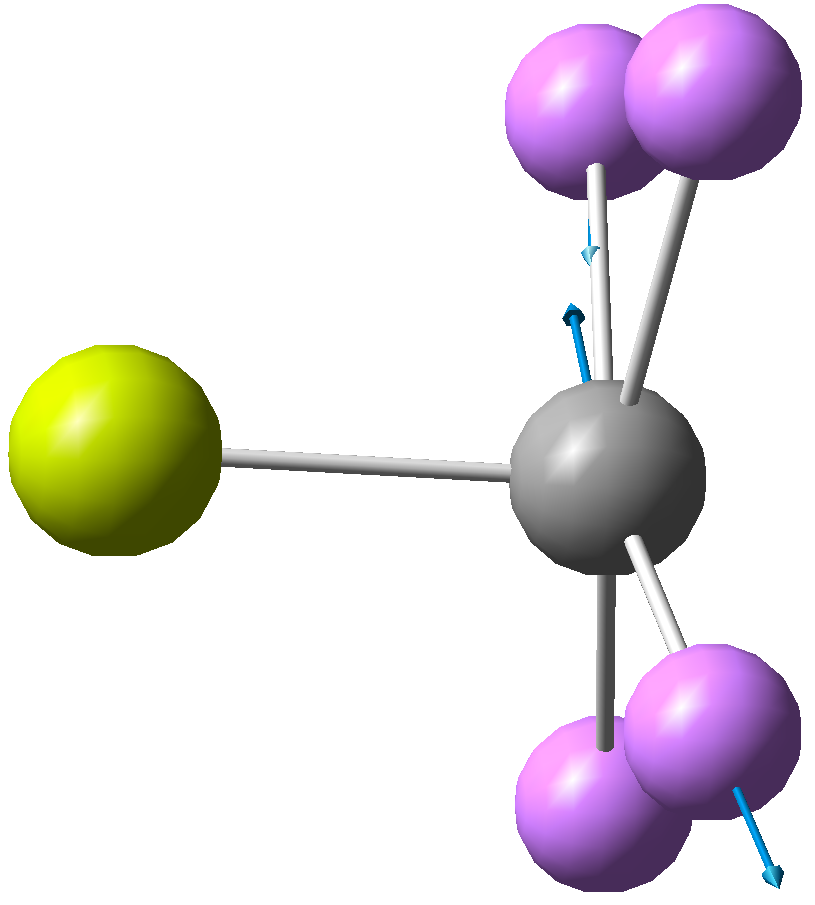 |
| 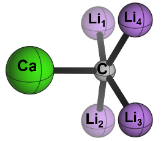  **CaCLi_4_ C_4v_** | | | |
| Assignment | Frequencies | Intensities |  |
| δ(Li-C-Li) | 69.2 | 0.0 | 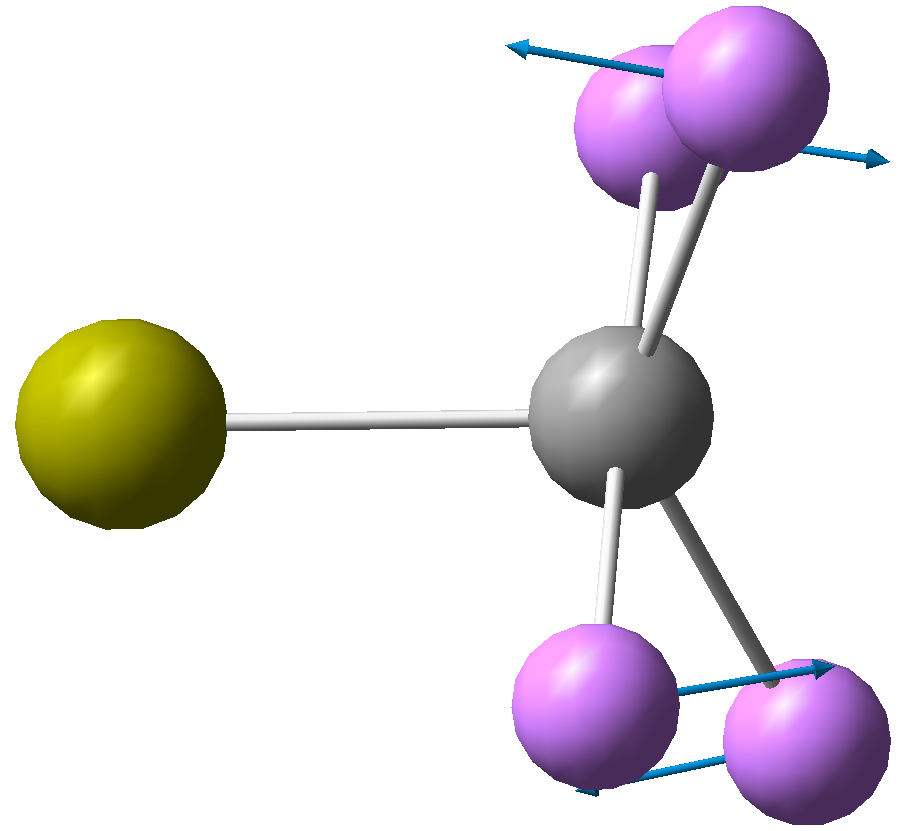 |
| δ(Li-C-Li-Ca) | 163.5 | 35.0 | 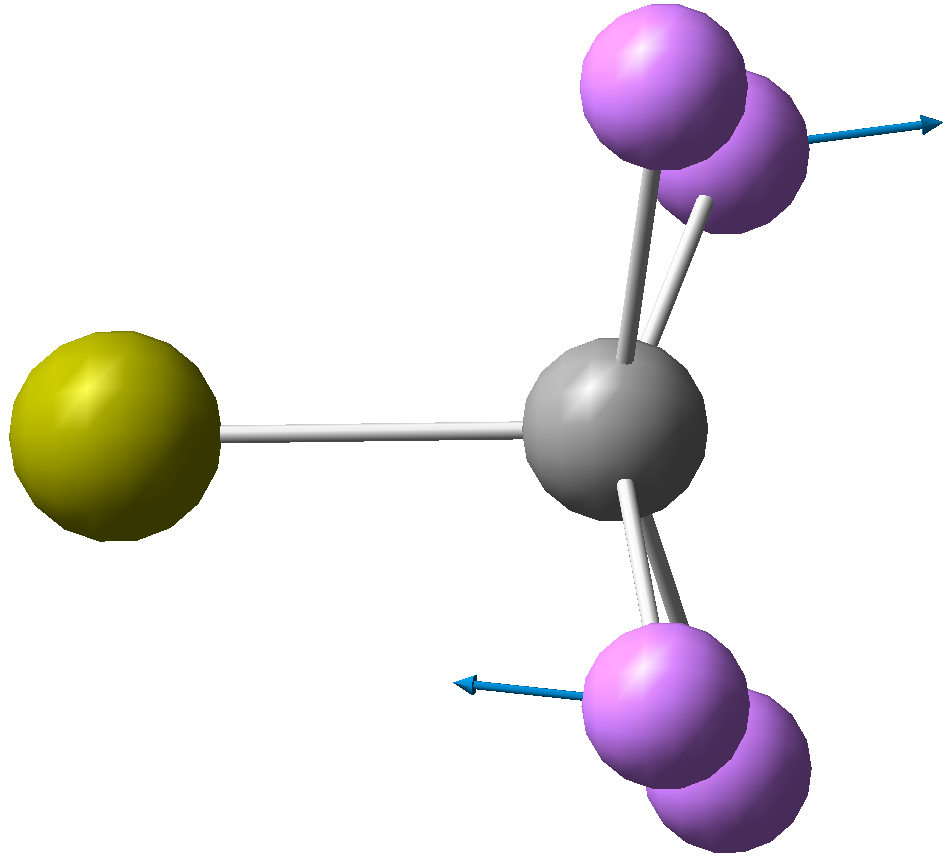 |
| δ(Li-C-Li-Ca) | 163.5 | 35.0 | 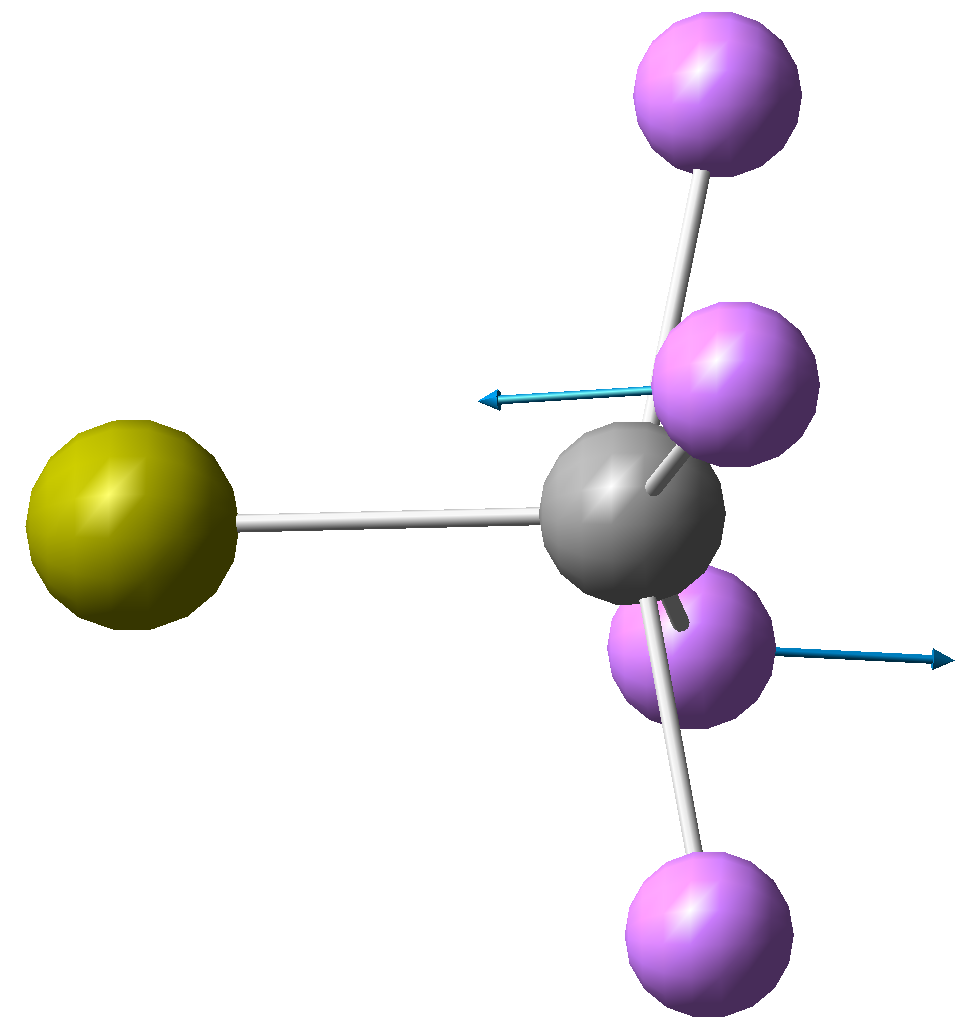 |
| δ(CLi_4_) | 207.1 | 13.9 | 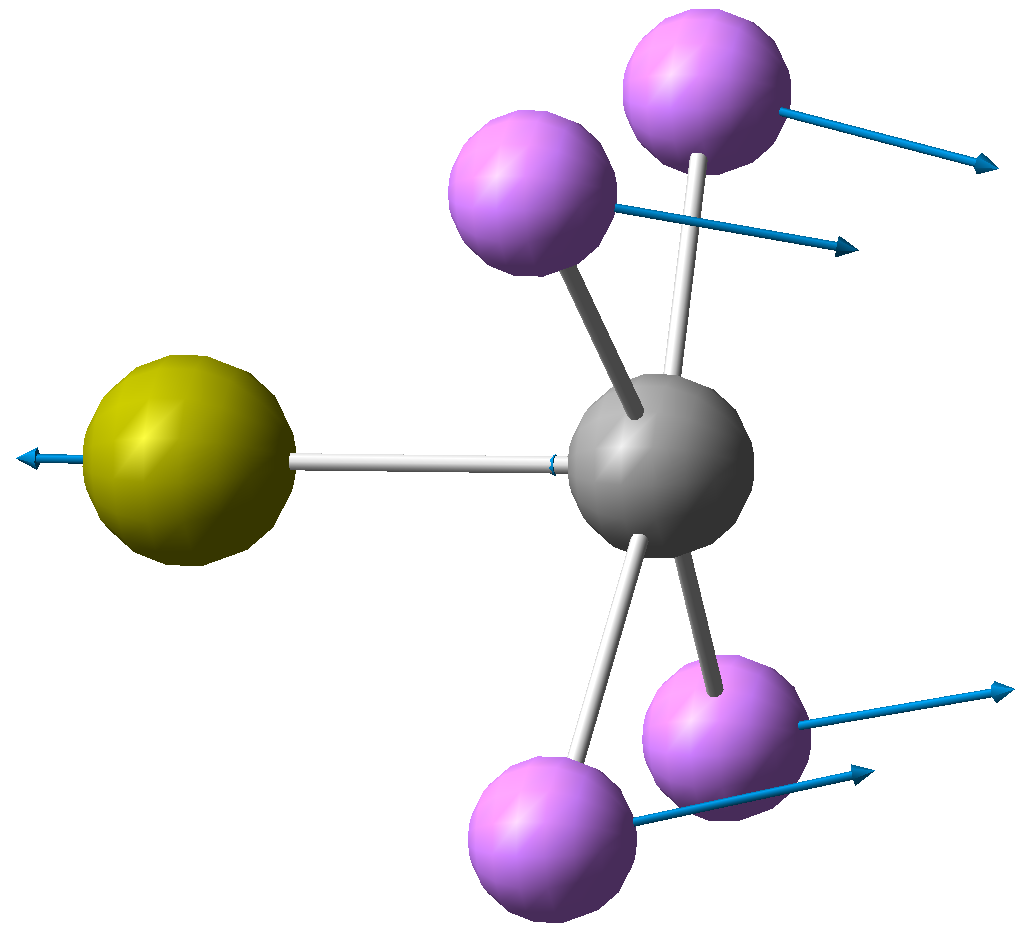 |
| δ(Li-C-Li) | 263.6 | 45.0 | 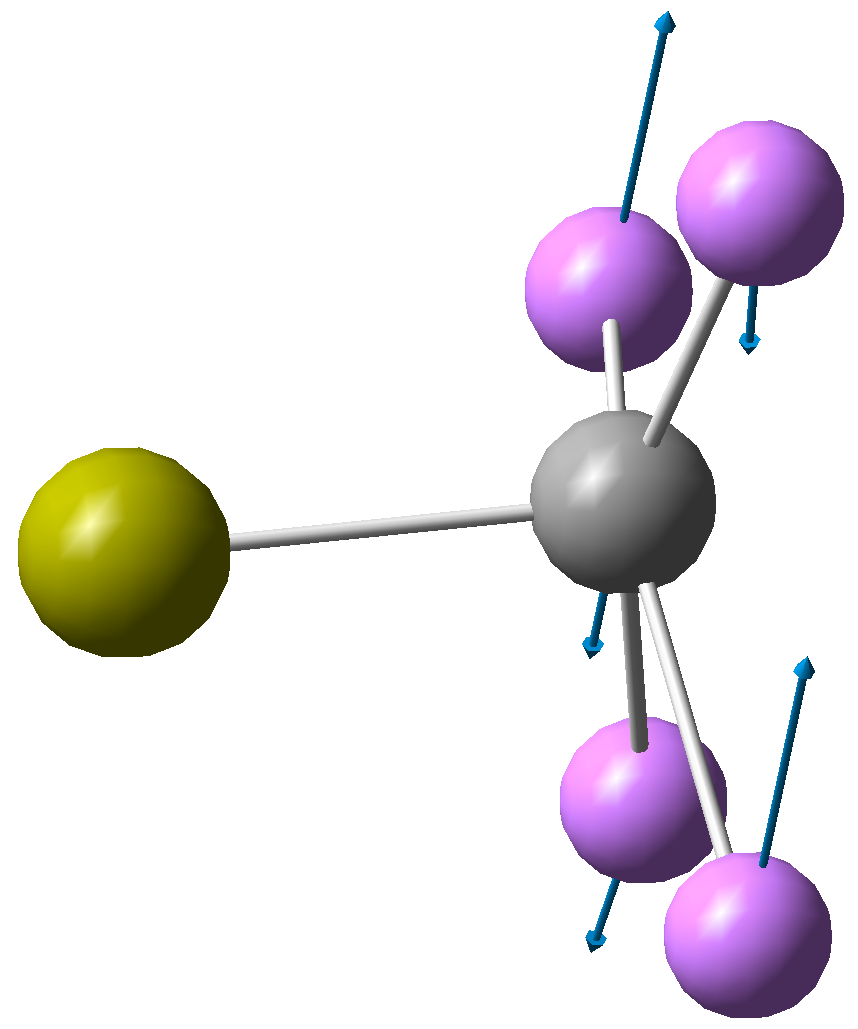 |
| δ(Li-C-Li) | 263.6 | 45.0 | 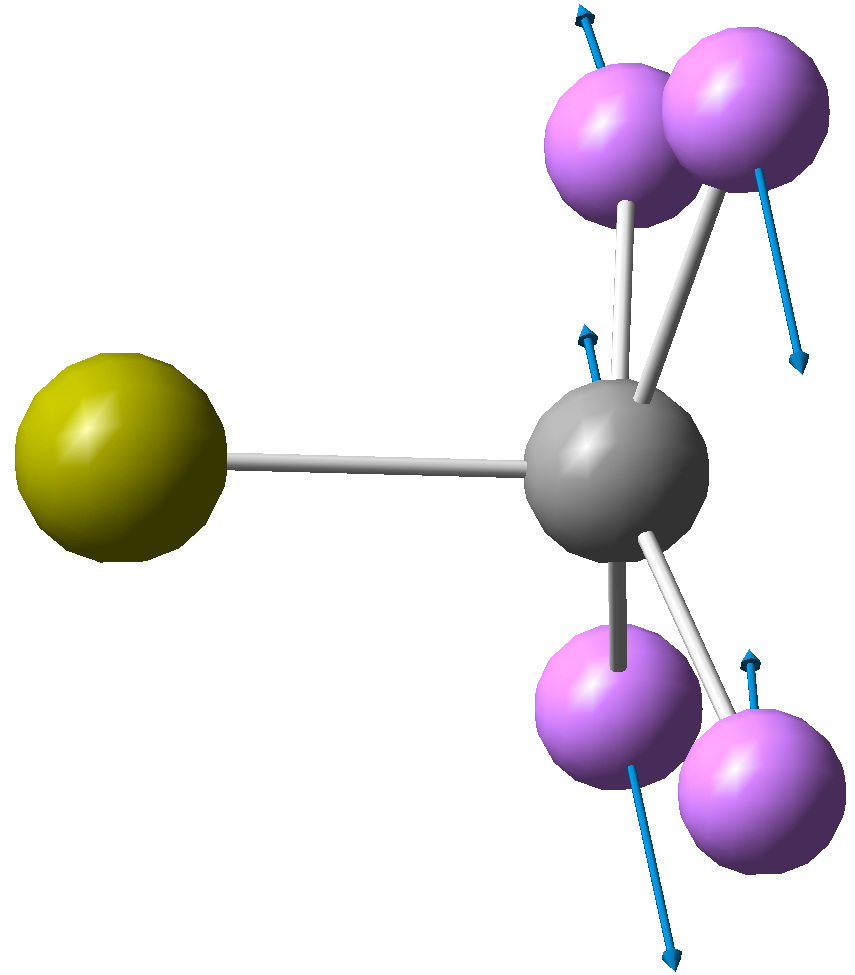 |
| δ(Li-C-Li) | 294.1 | 0.0 | 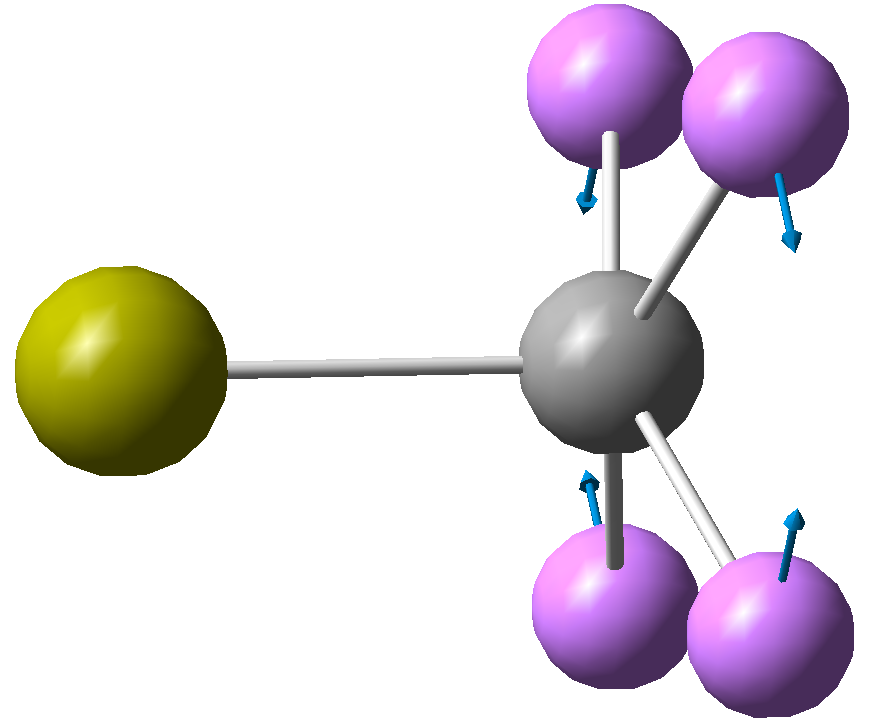 |
| ν(Li-C-Li) | 452.3 | 0.0 | 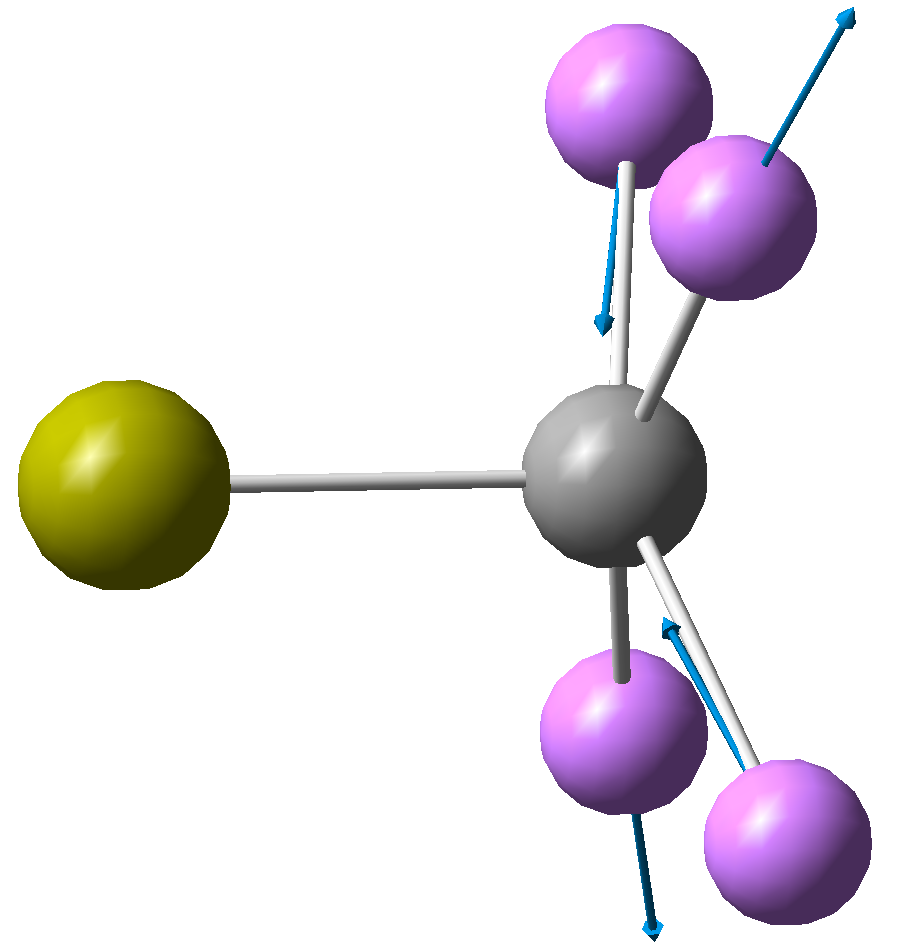 |
| ν(Li-C-Li) | 455.0 | 1.9 | 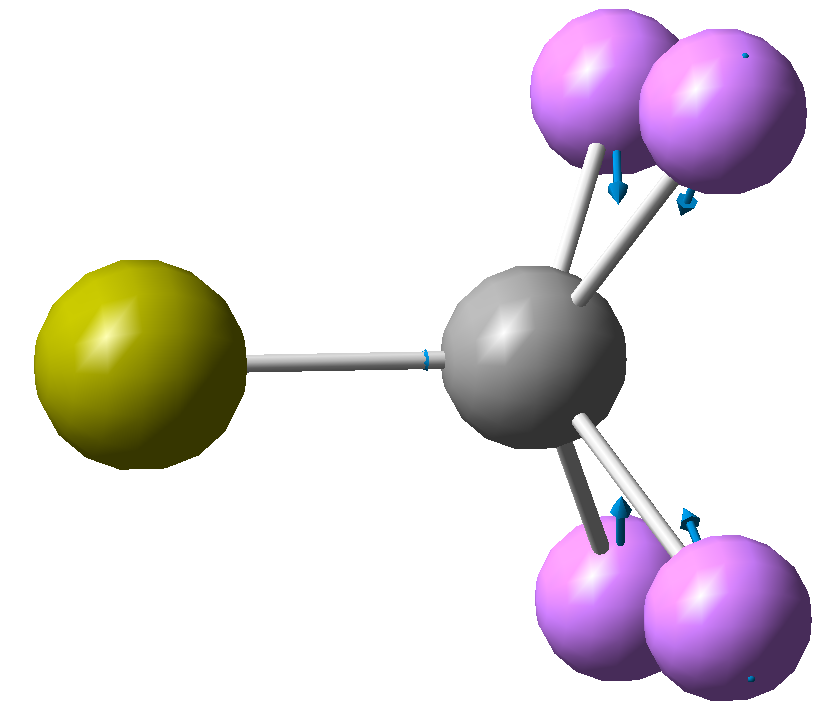 |
| ν(C-Ca) | 550.3 | 17.1 | 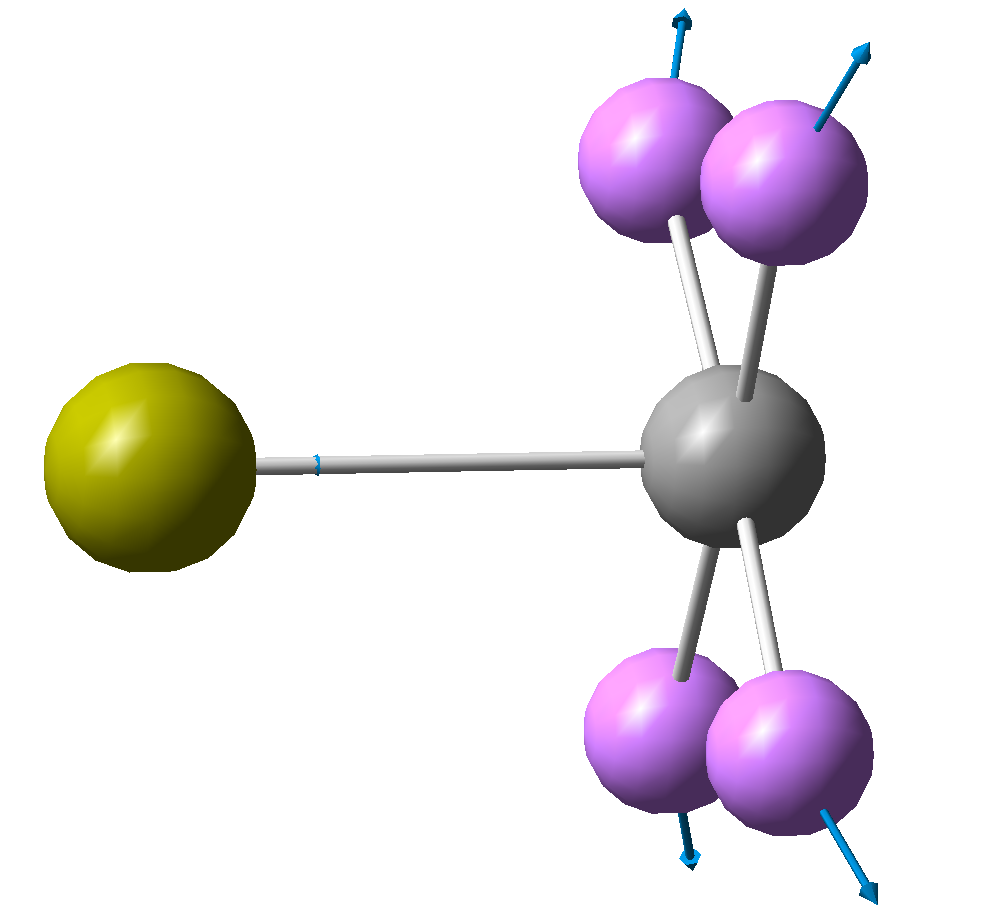 |
| ν(Li-C-Li) | 641.6 | 3.9 | 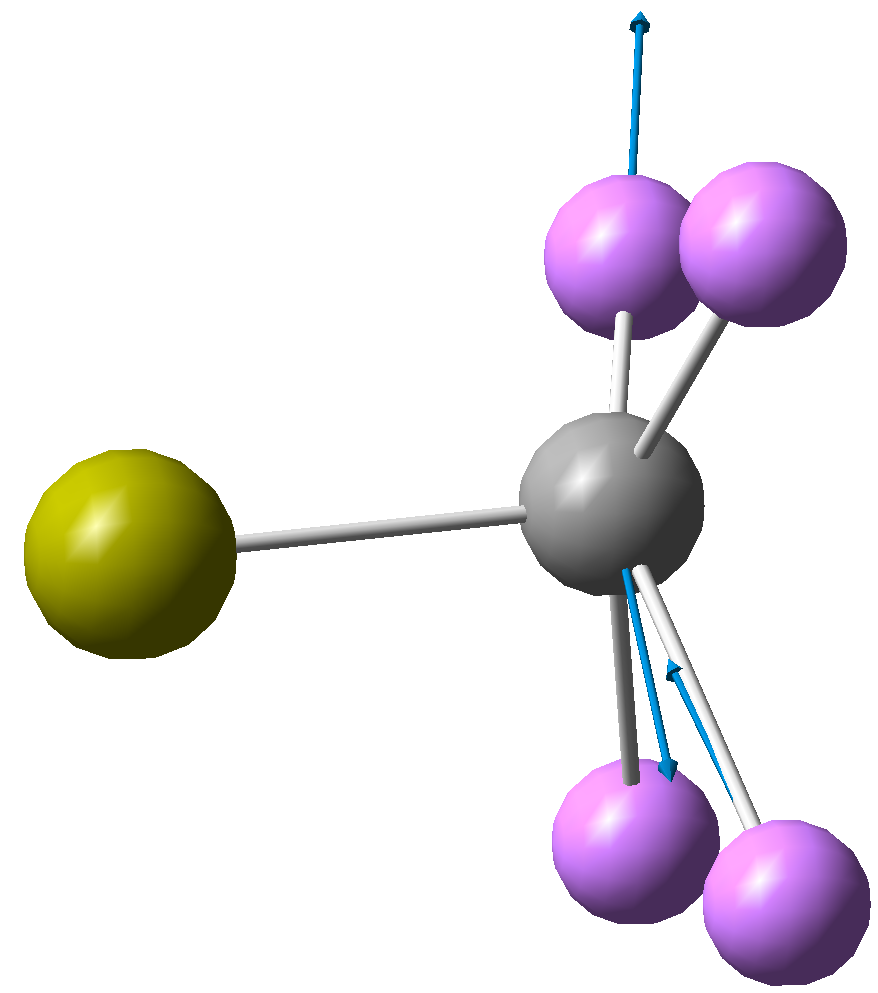 |
| ν(Li-C-Li) | 641.6 | 3.9 | 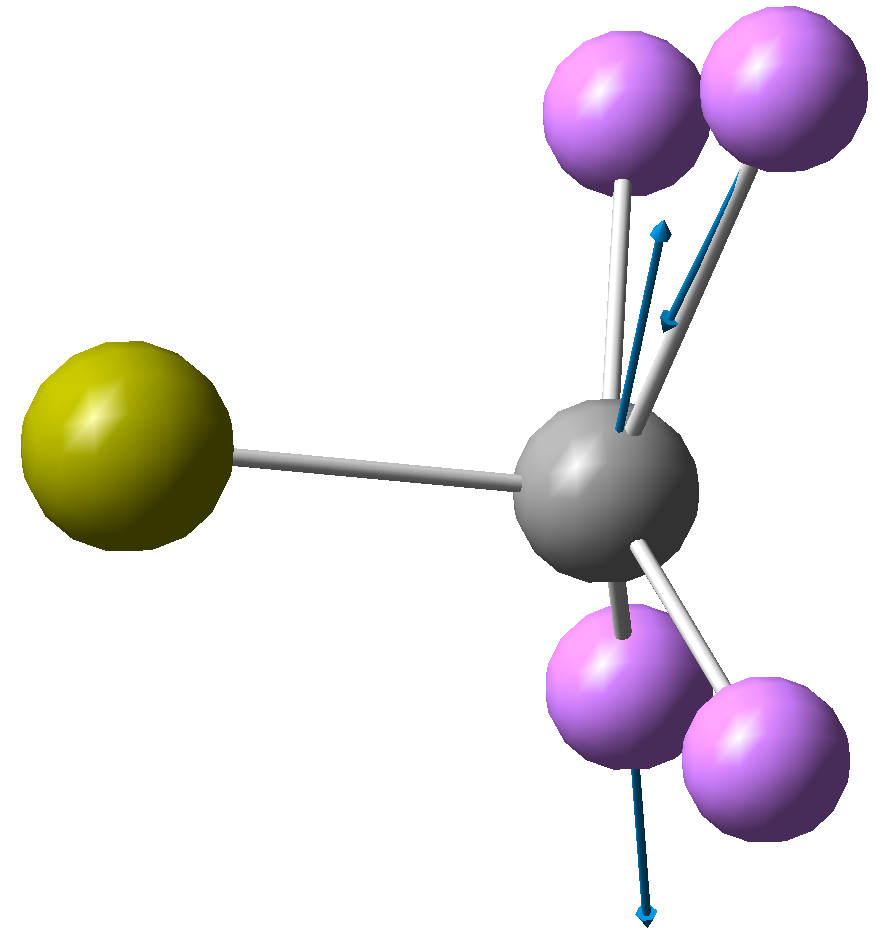 |
| 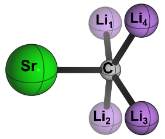  **SrCLi_4_ C_4v_** | | | |
| Assignment | Frequencies | Intensities |  |
| δ(Li-C-Li) | 60.1 | 0.0 | 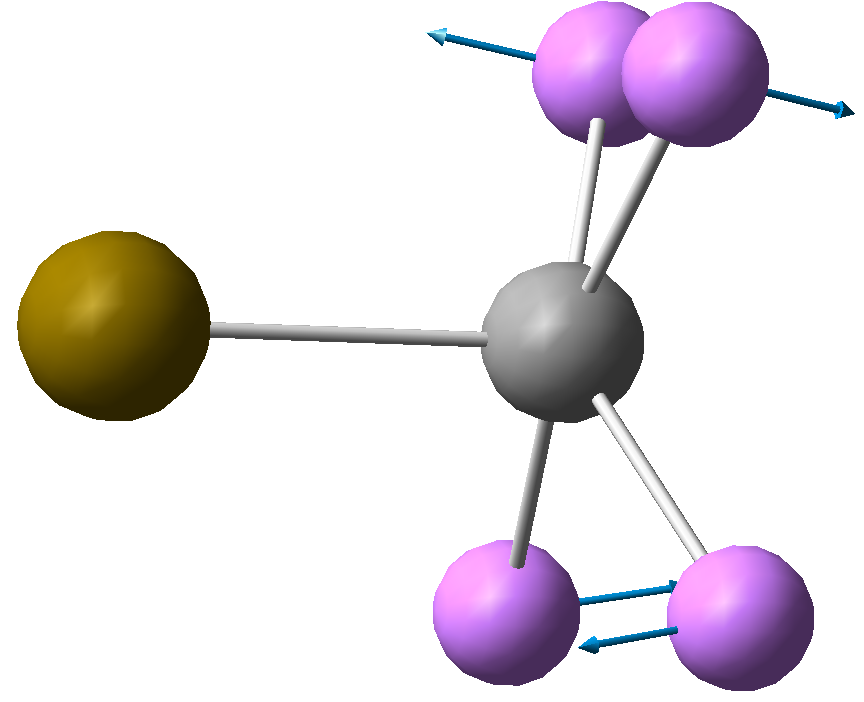 |
| δ(Li-C-Li-Sr) | 153.1 | 30.8 | 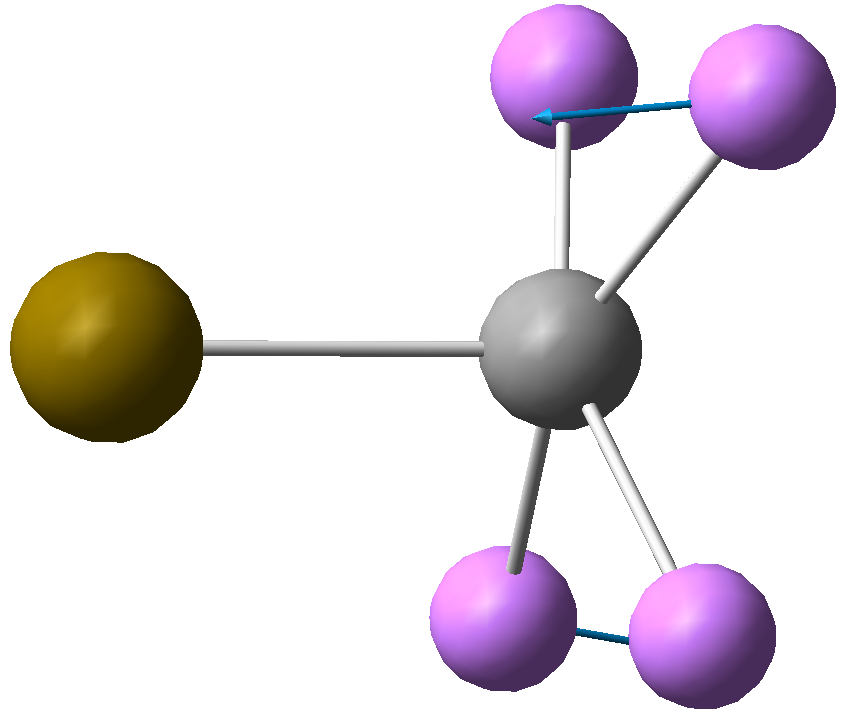 |
| δ(Li-C-Li-Sr) | 153.1 | 30.8 | 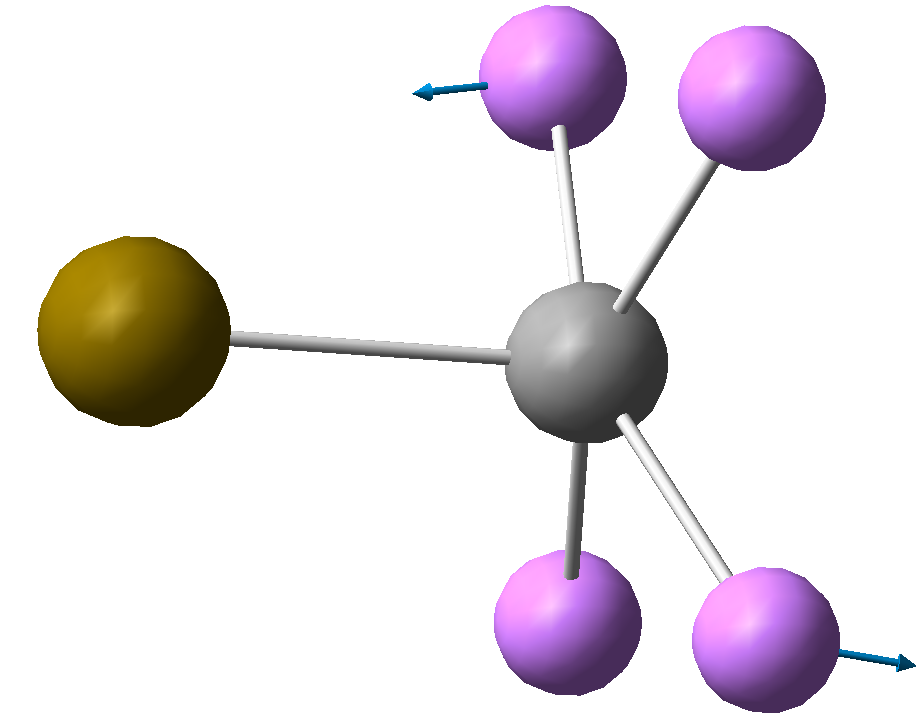 |
| δ(CLi_4_) | 184.3 | 8.0 | 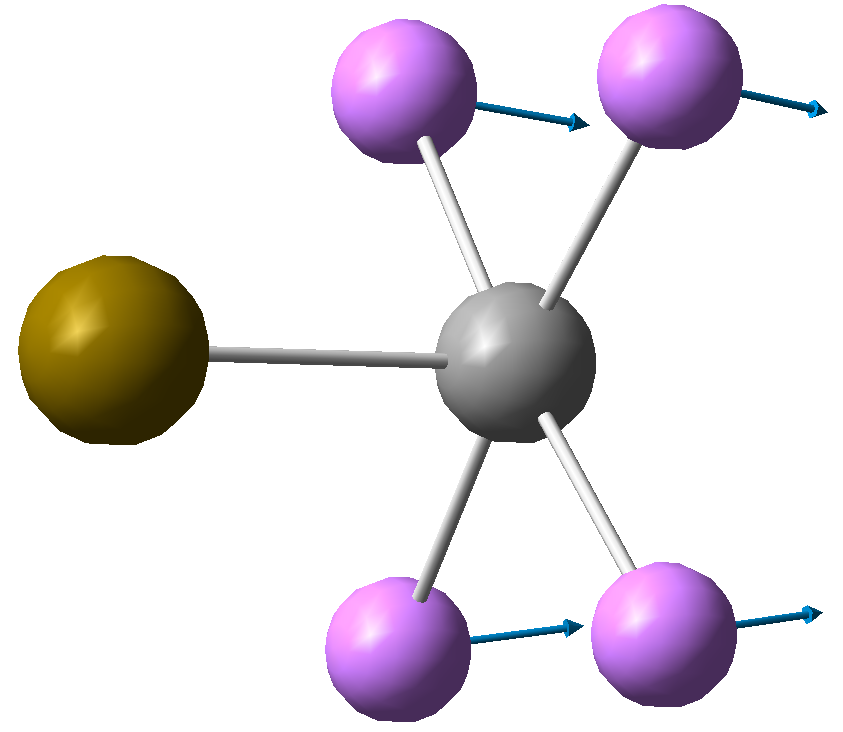 |
| δ(Li-C-Li) | 266.4 | 39.6 | 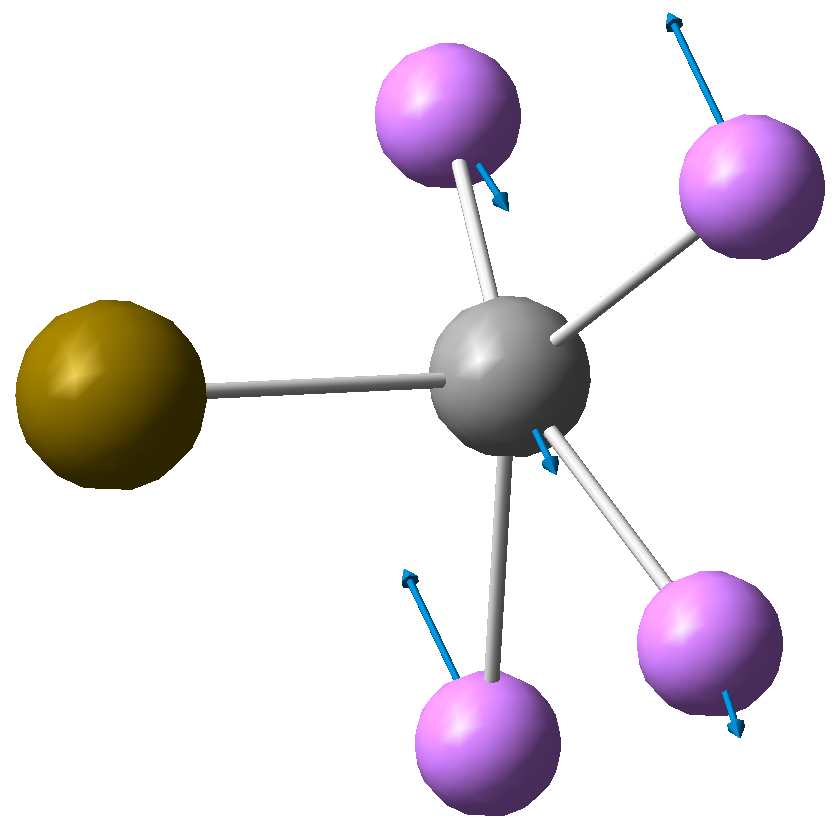 |
| δ(Li-C-Li) | 266.4 | 39.6 | 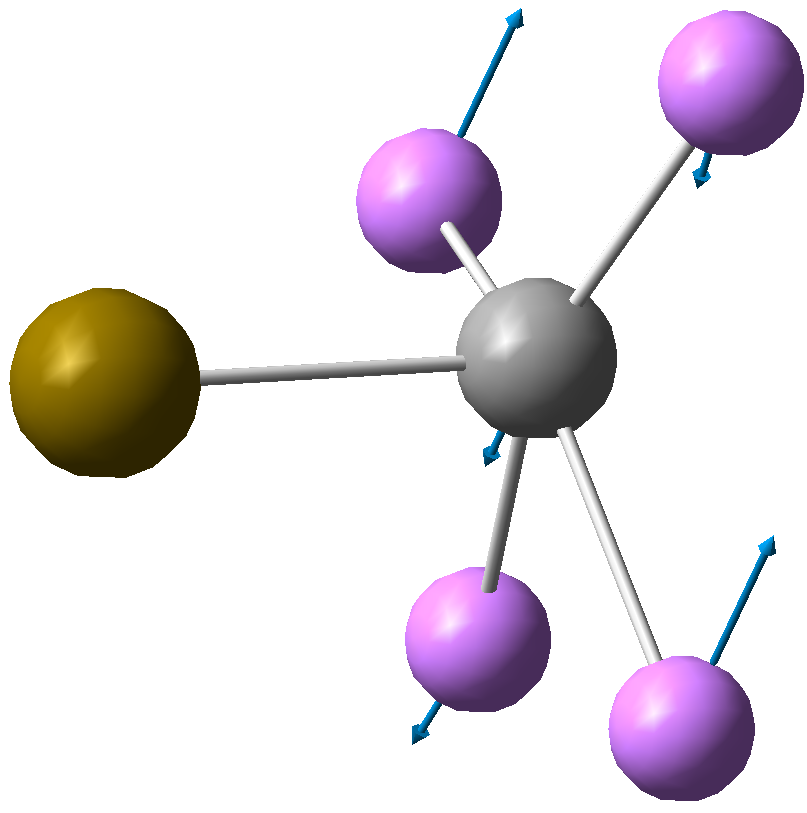 |
| δ(Li-C-Li) | 297.6 | 0.0 | 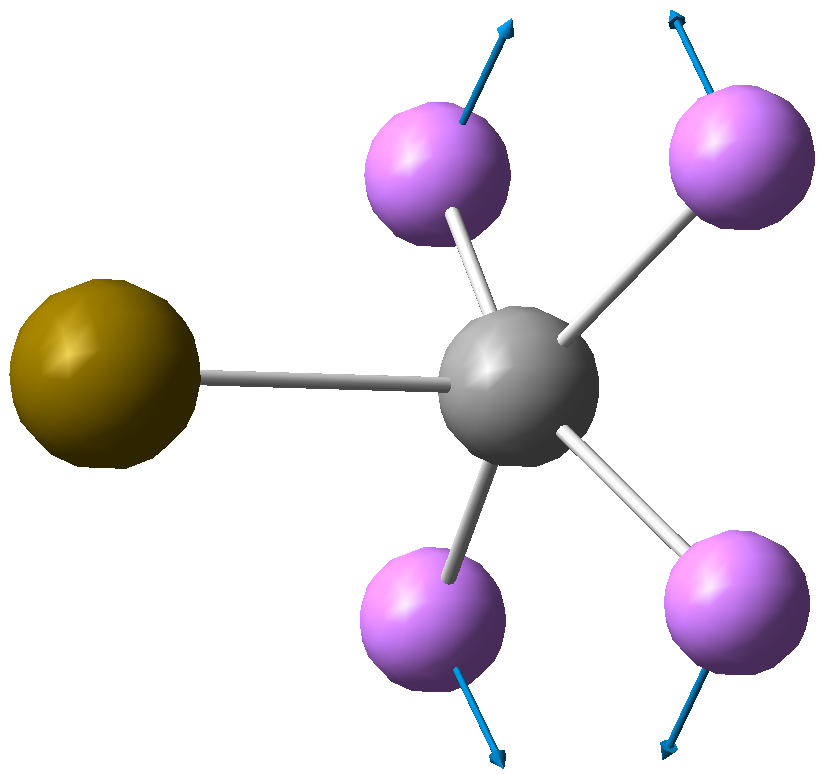 |
| ν(Sr-C) | 415.2 | 12.6 | 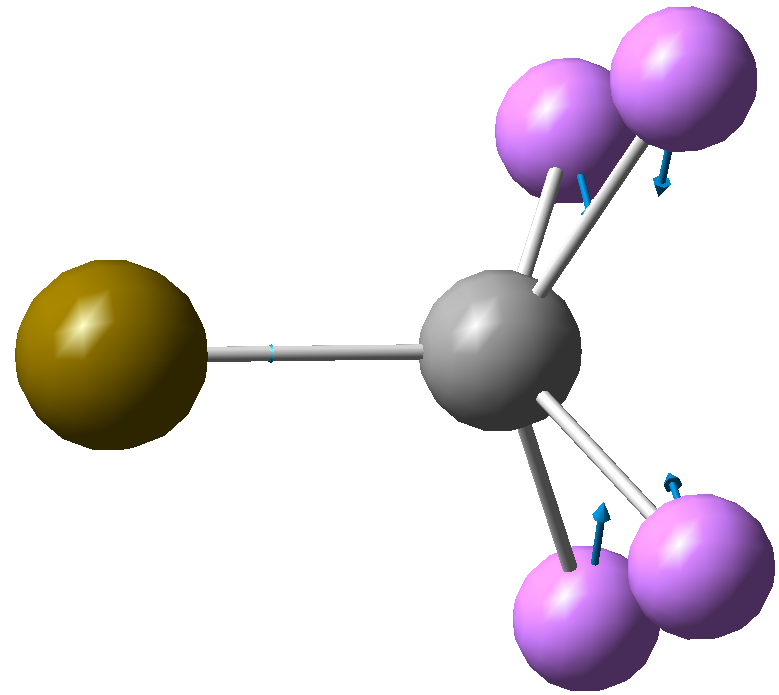 |
| ν(Li-C-Li) | 450.6 | 0.0 | 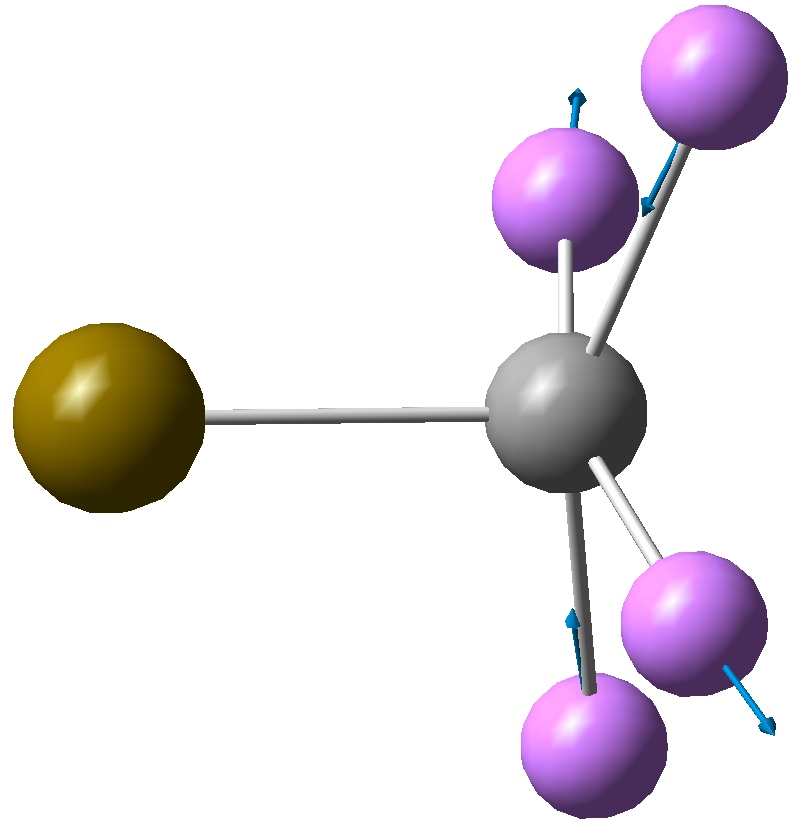 |
| ν(Li-C-Li) | 527.2 | 20.8 | 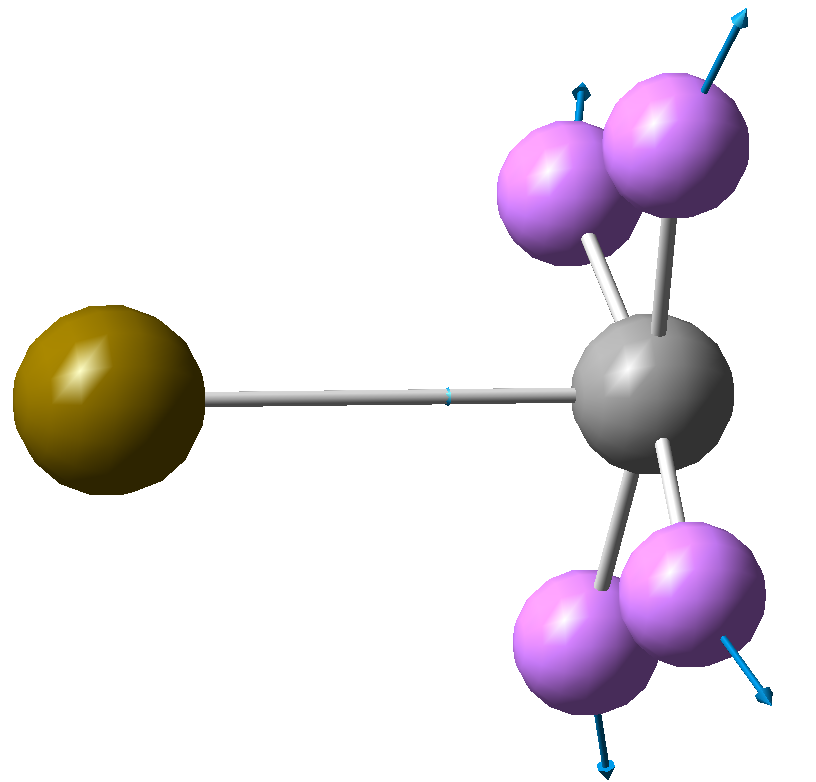 |
| ν(Li-C-Li) | 641.3 | 3.9 | 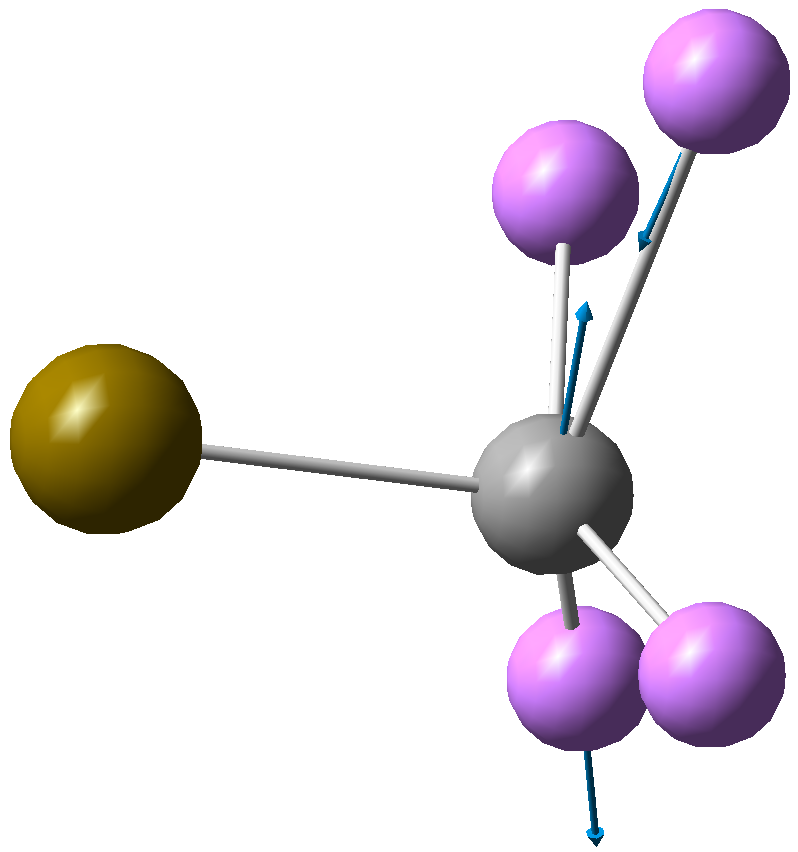 |
| ν(Li-C-Li) | 641.3 | 3.9 | 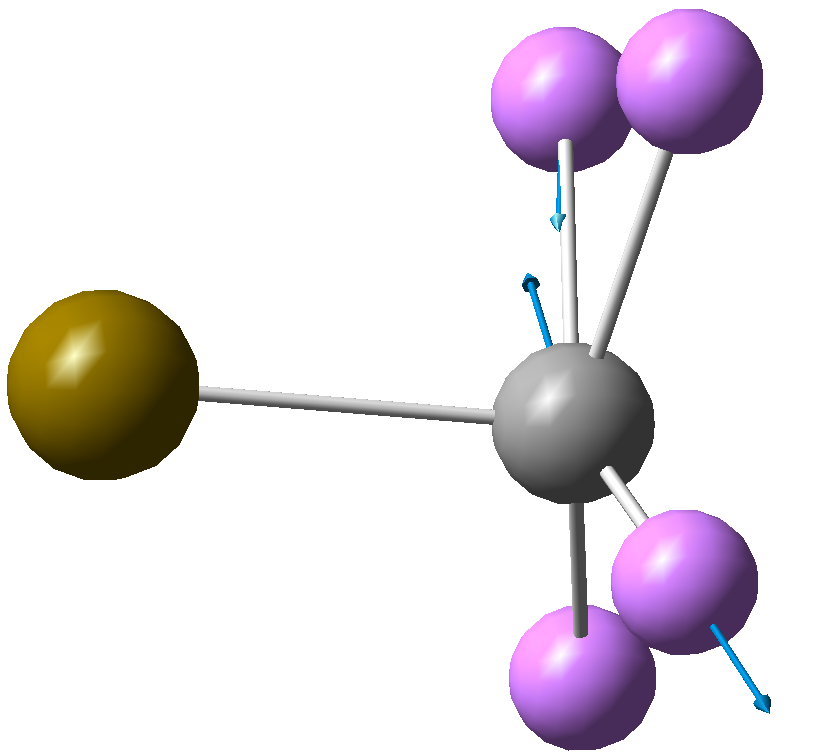 |
| 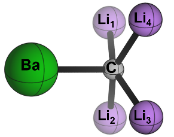  **BaCLi_4_ C_4v_** | | | |
| Assignment | Frequencies | Intensities |  |
| δ(Li-C-Li) | 64.5 | 0.0 | 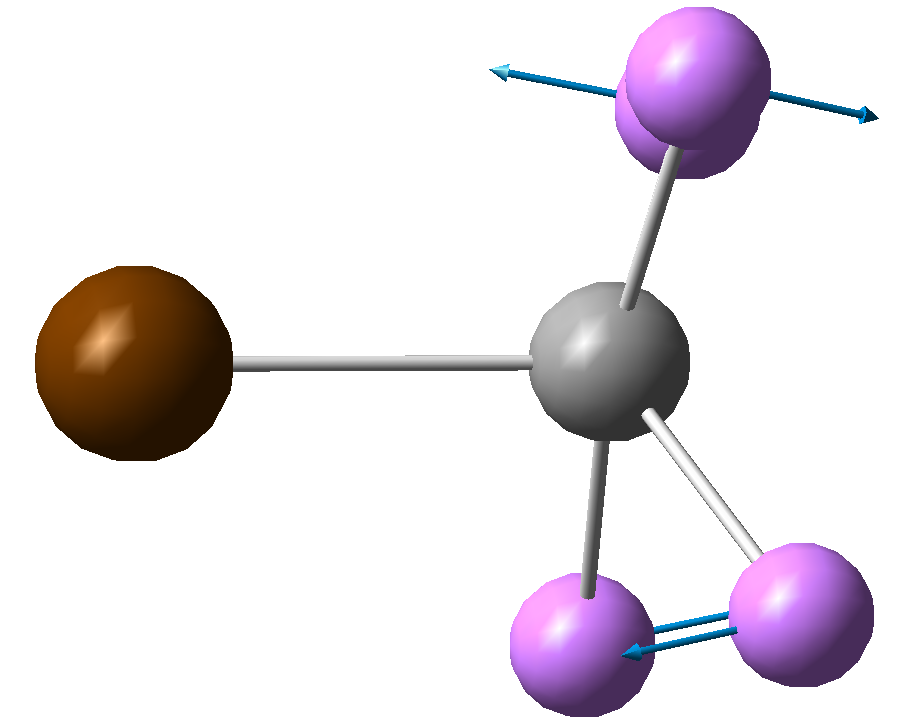 |
| δ(Li-C-Li-Ba) | 161.1 | 30.2 | 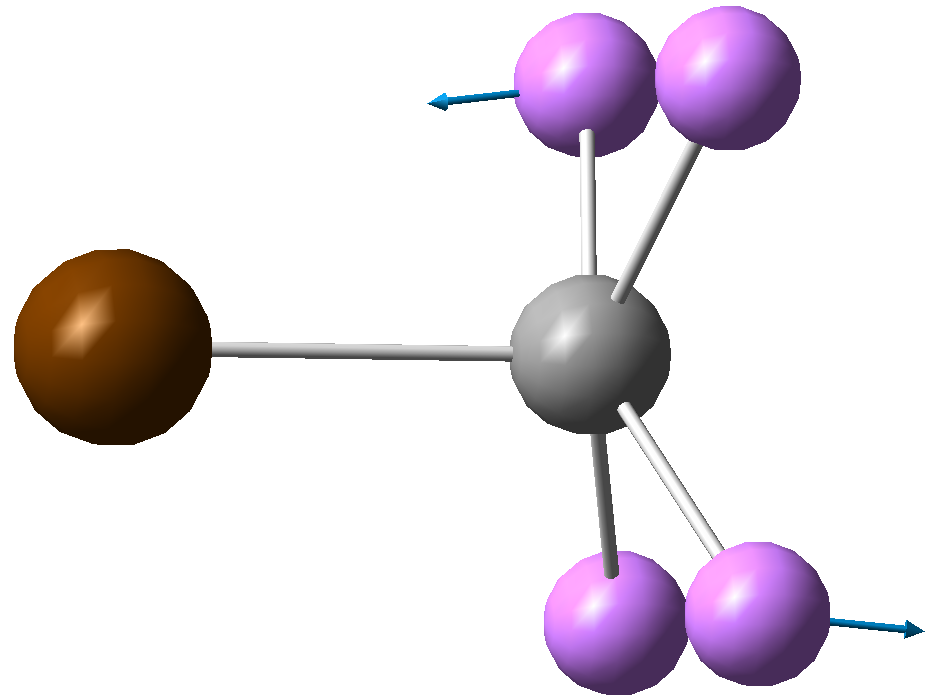 |
| δ(Li-C-Li-Ba) | 161.1 | 30.2 | 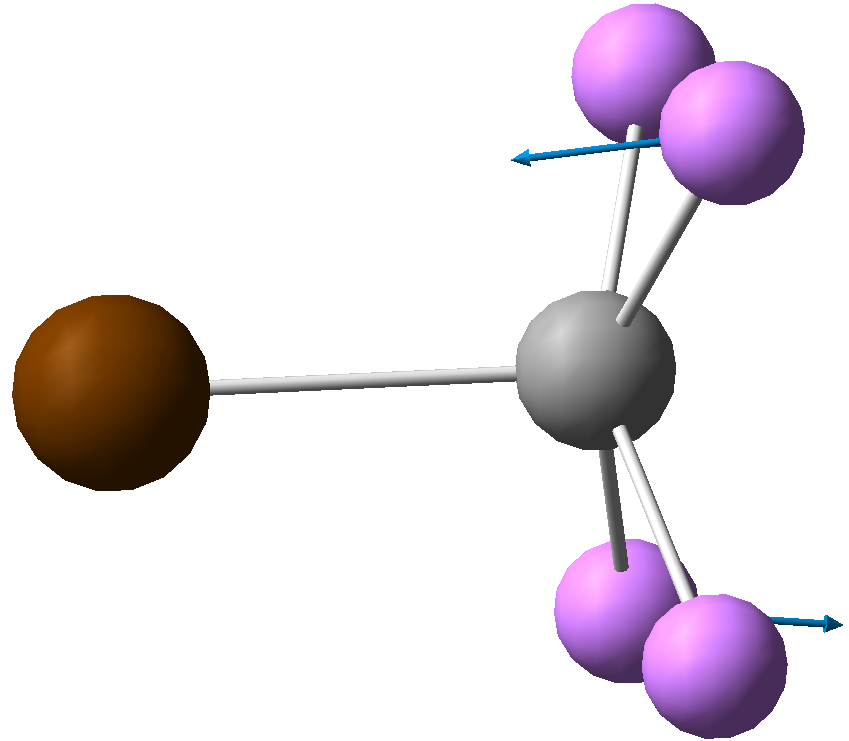 |
| δ(CLi_4_) | 184.3 | 0.3 | 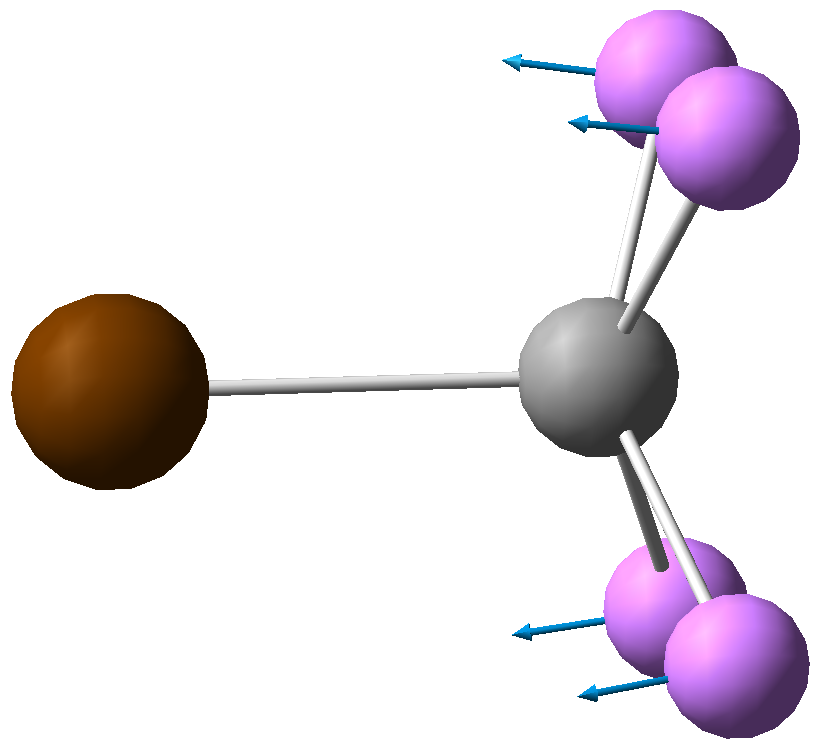 |
| δ(Li-C-Li) | 265.2 | 48.3 | 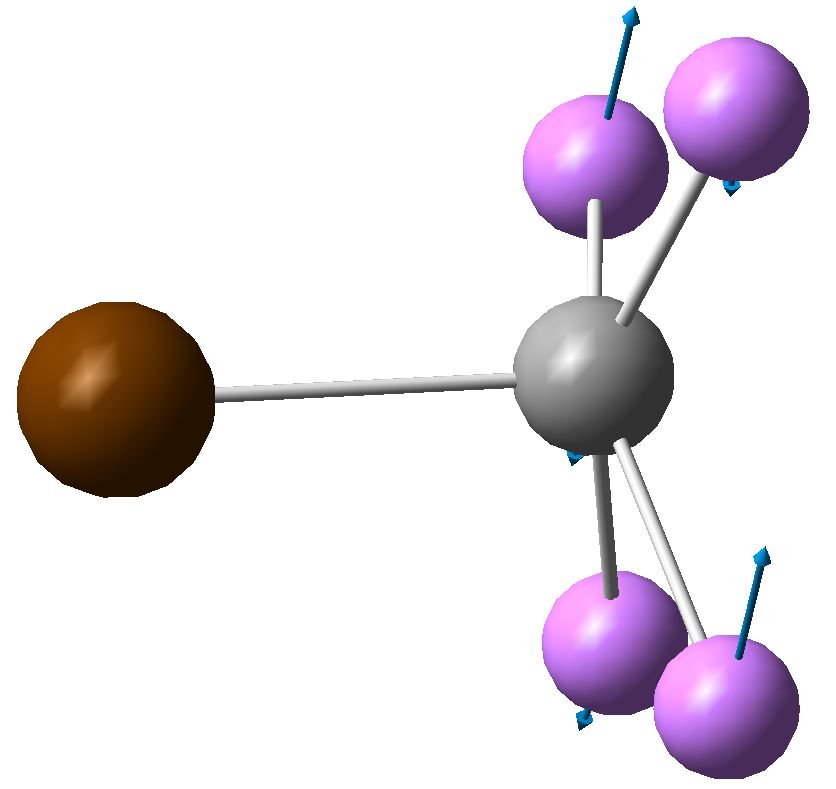 |
| δ(Li-C-Li) | 265.2 | 48.3 | 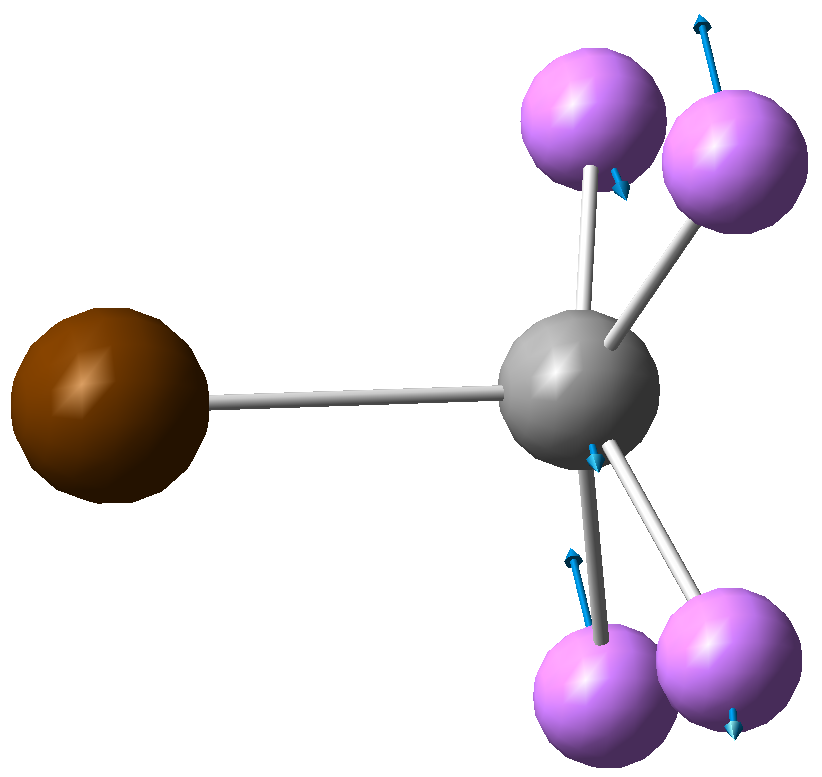 |
| δ(Li-C-Li) | 292.5 | 0.0 | 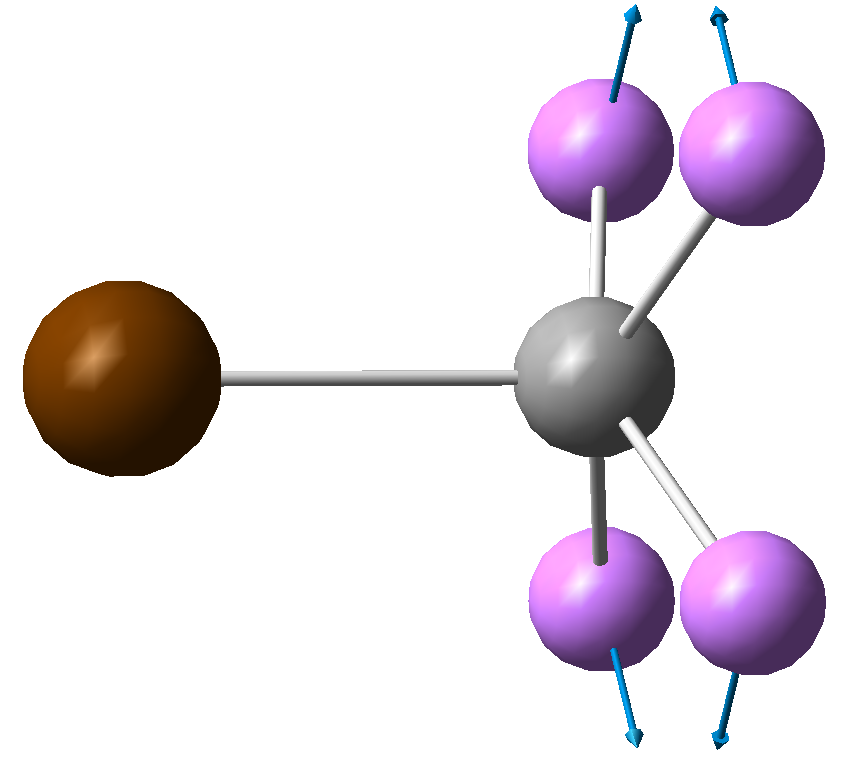 |
| ν(Ba-C) | 400.2 | 18.6 | 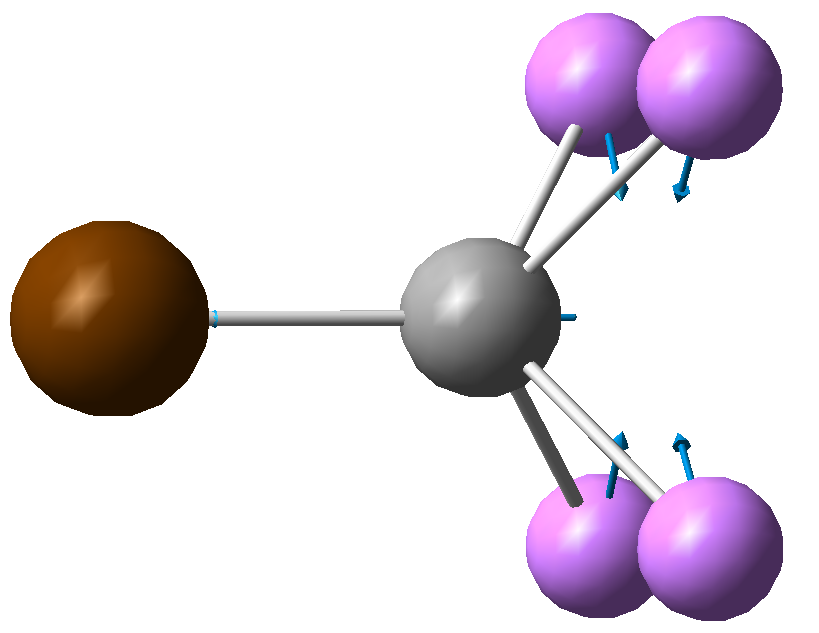 |
| ν(Li-C-Li) | 443.7 | 0.0 | 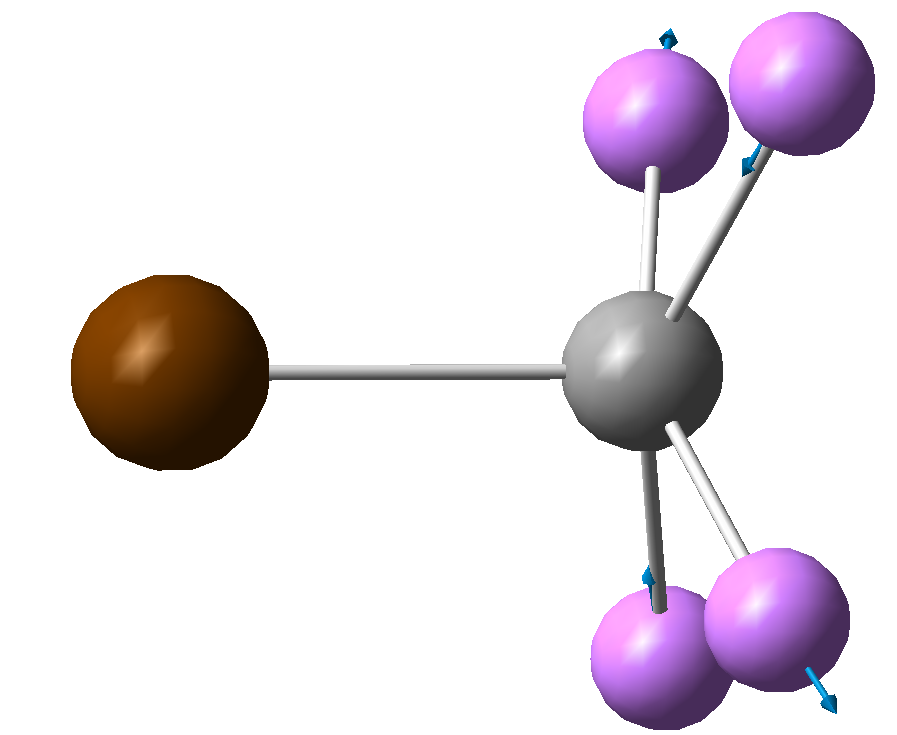 |
| ν(Li-C-Li) | 529.3 | 52.1 | 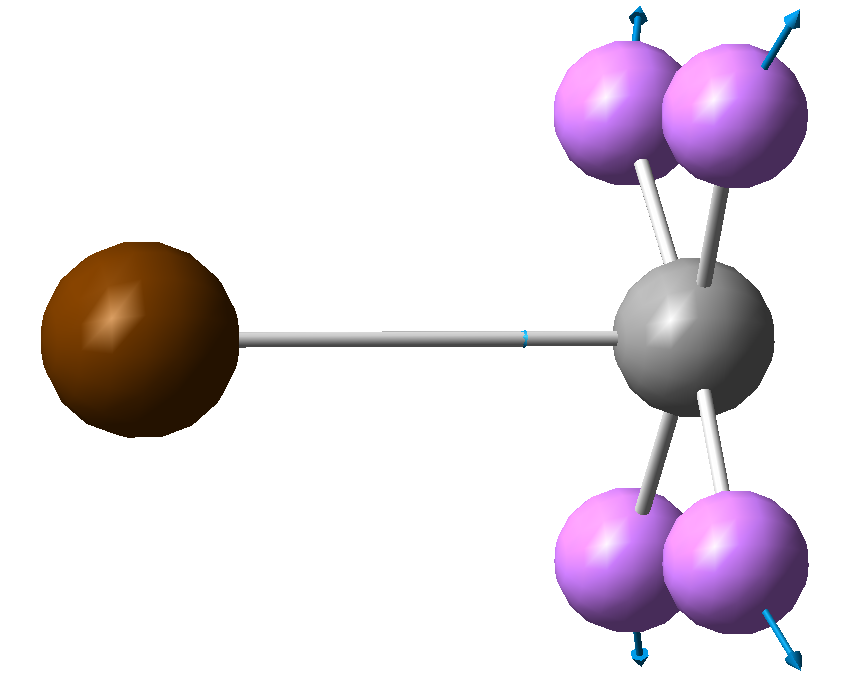 |
| ν(Li-C-Li) | 629.2 | 0.5 | 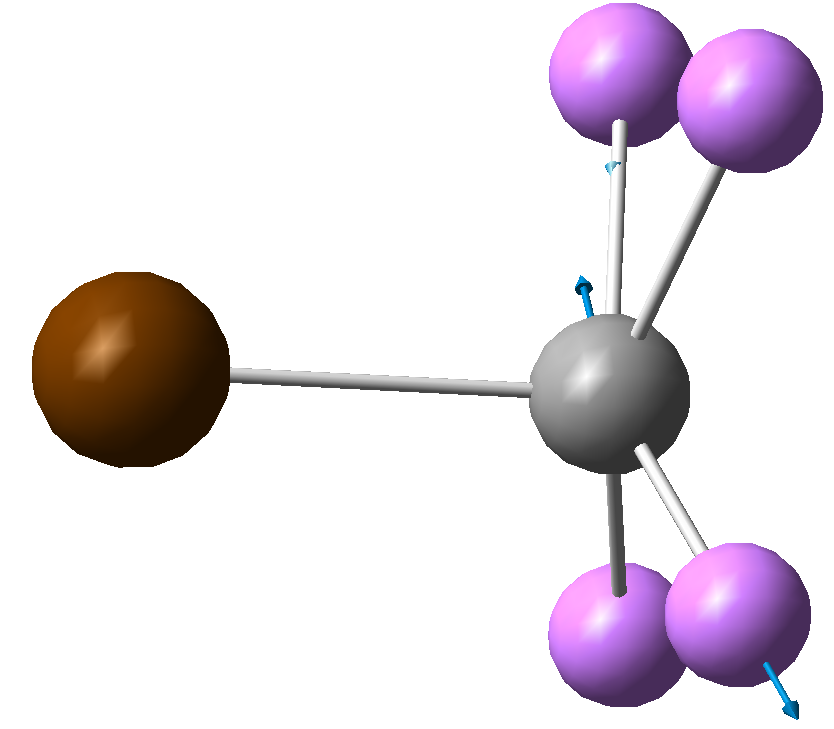 |
| ν(Li-C-Li) | 629.2 | 0.5 | 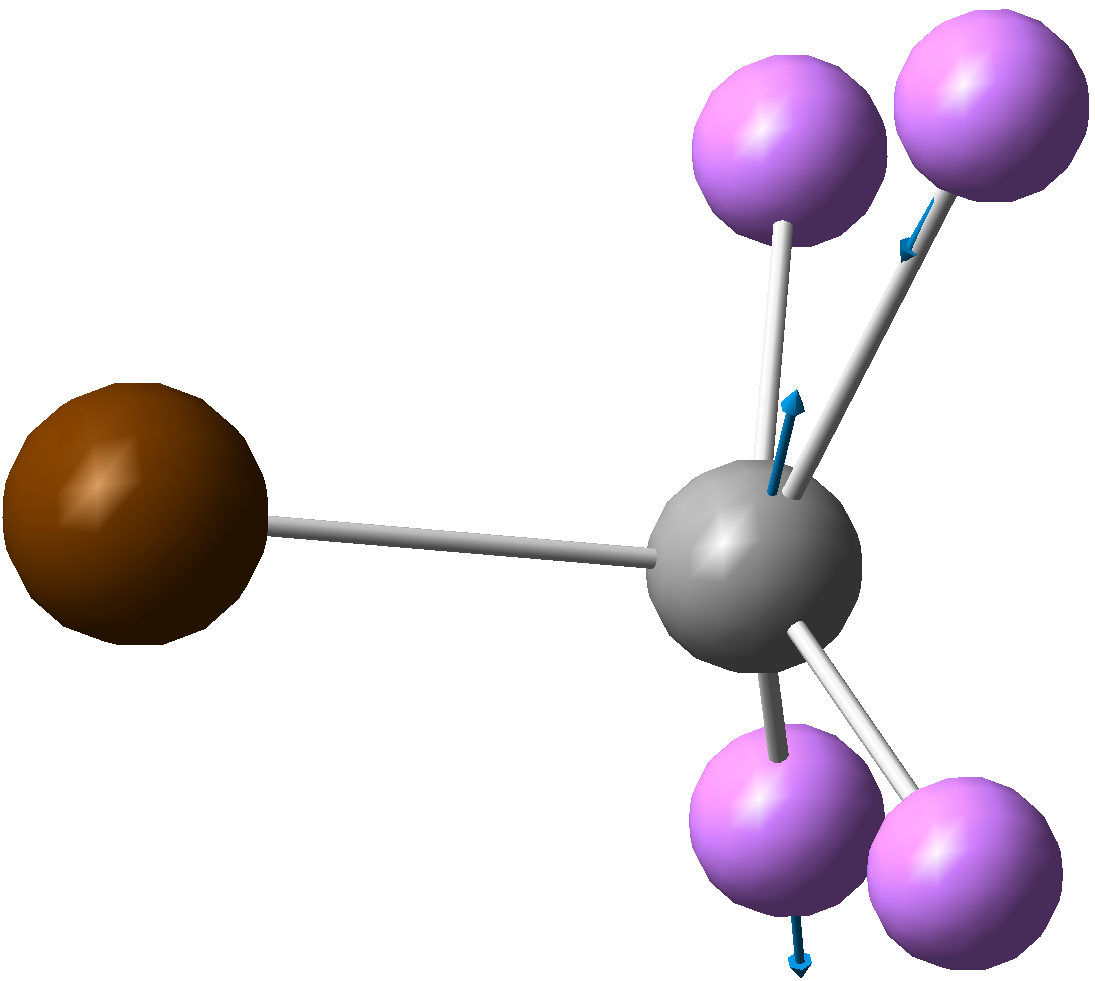 |

|  | Deformation density | Orbital | | | |
| --- | --- | --- | --- | --- | --- |
|  |  | MgCLi_4_ | Mg |  | CLi_4_ |
| ∆ρ_(1)_ | 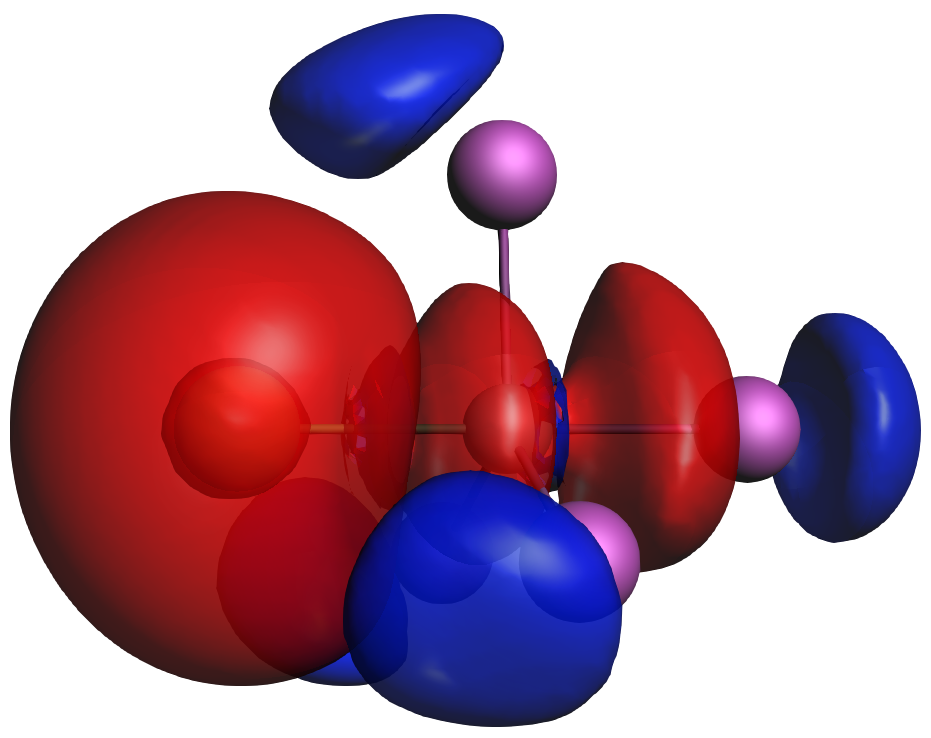  ∆E_orb(1)_ = -34.5 kcal/mol  \|ν_1_\| = 0.75 | 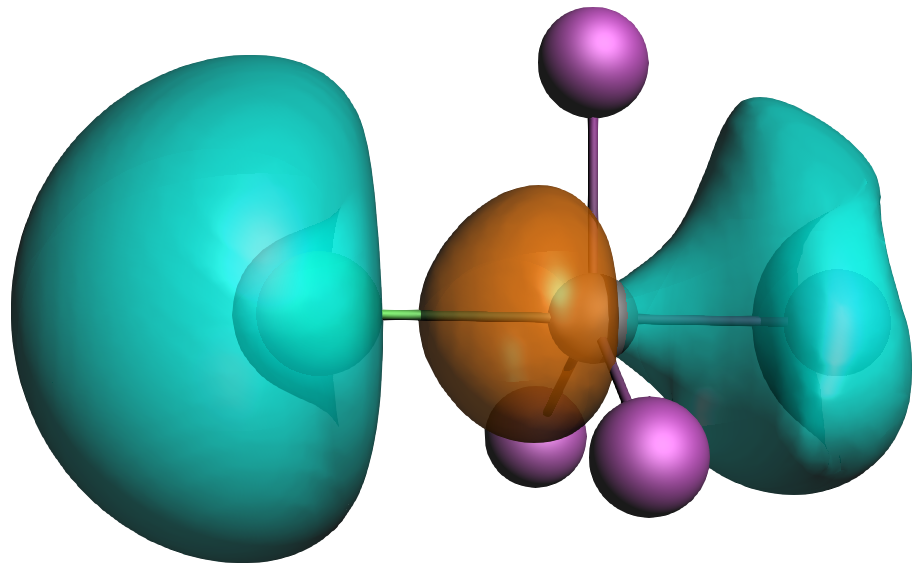  HOMO | 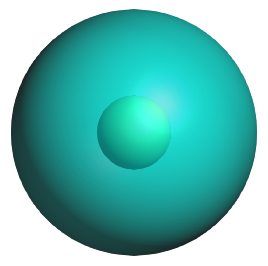  HOMO s  ν = -0.62 | **→** | 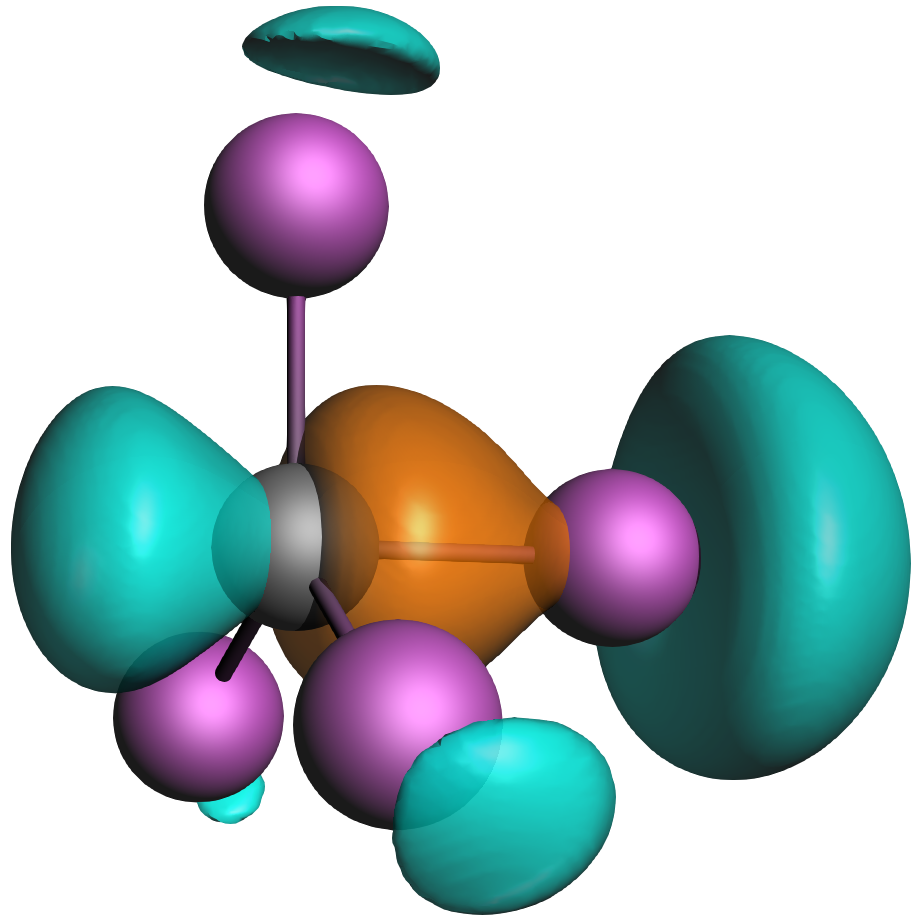  LUMO  ν = 0.51 |
| ∆ρ_(2)_ | 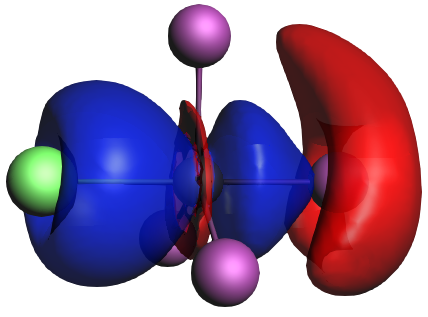  ∆E_orb(2)_ = -13.1 kcal/mol  \|ν_2_\| = 0.381 | 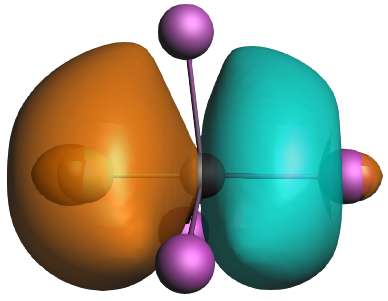  HOMO-2 | 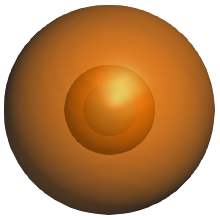  LUMO+1  ν = 0.10  +  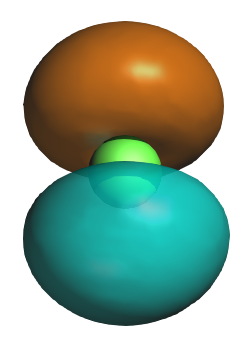  LUMO p_σ_  ν = 0.030 | **←** | 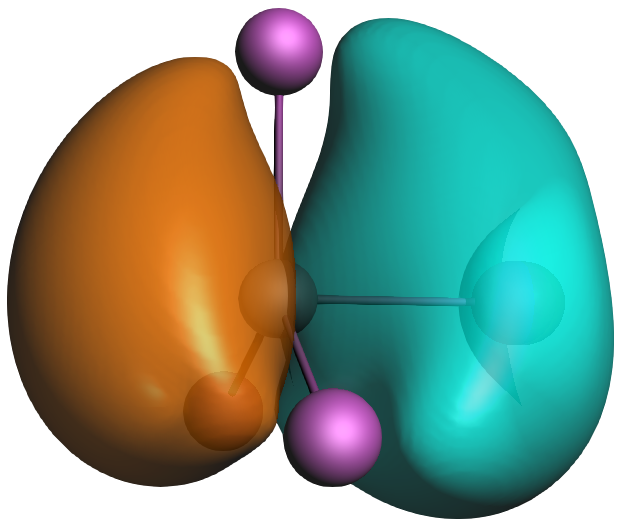  HOMO  ν = -0.18  +    HOMO-2  ν = -0.03  +    LUMO+3  ν = 0.03 |
| ∆ρ_(3)_ | ∆E_orb(3)_ = -10.1 kcal/mol  \|ν_3_\| = 0.38 | HOMO-1 | LUMO p_π_  ν = 0.15 | **←** | HOMO-1  ν = -0.20 |
| ∆ρ_(4)_ | ∆E_orb(4)_ = -10.1 kcal/mol  \|ν_4_\| = 0.38 | HOMO-1′ | LUMO′ p_π′_  ν = 0.110 | **←** | HOMO-1′  ν = -0.20 |

**Figure S2.** Plot of the deformation densities, ∆ρ_(1)-(4)_ shown as the sum of α and β electronic charge corresponding to ∆E_orb(1)-(4)_ and the related interacting orbitals in the singlet states of *C*_3_*_v_* symmetric MgCLi_4_ at the BP86-D3(BJ)/TZ2P-ZORA//BP86-D3(BJ)/def2-QZVPP level using Mg (3s^2^, ^1^S) + CLi_4_ (^1^A_1_) as interacting fragments. The eigenvalues ν indicate the size of the charge flow. The direction of charge flow is red → blue. The isovalue for ∆ρ_(1)-(4)_ is 0.0005 au.

|  | Deformation density | Orbital | | | |
| --- | --- | --- | --- | --- | --- |
|  |  | MgCLi_4_ | Mg |  | CLi_4_ |
| ∆ρ_(1)_ | ∆E_orb(1)_ = -63.5 kcal/mol  \|ν_1_\| = 1.10 | HOMO | HOMO s  ν = -0.72    LUMO p_σ_  ν = 0.14 | **→** | LUMO  ν = 0.82 |
|  |  |  |  |  | HOMO  ν = -0.47 |
| ∆ρ_(2)_ | ∆E_orb(2)_ = -13.5 kcal/mol  \|ν_2_\| = 0.34 | HOMO-2 | HOMO s  ν = 0.08  +    LUMO p_σ_  ν = 0.02 | **←** | HOMO  ν = -0.12  +    HOMO-2  ν = -0.03  +    LUMO+6  ν = 0.02 |
| ∆ρ_(3)_ | ∆E_orb(3)_ = -10.0 kcal/mol  \|ν_3_\| = 0.38 | HOMO-1 | LUMO p_π_  ν = 0.16 | **←** | HOMO-1  ν = -0.20 |
| ∆ρ_(4)_ | ∆E_orb(4)_ = -10.0 kcal/mol  \|ν_4_\| = 0.38 | HOMO-1′ | LUMO p_π′_  ν = 0.16 | **←** | HOMO-1**^′^**  ν = -0.20 |

**Figure S3.** Plot the deformation densities of MgCLi_4_ with C₄ᵥ symmetry, ∆ρ_(1)-(4)_ shown as the sum of α and β electronic charge corresponding to ∆E_orb(1)-(4)_ and the related interacting orbitals in the singlet states of MgCLi_4_ at the BP86-D3(BJ)/TZ2P-ZORA//BP86-D3(BJ)/def2-QZVPP level using Mg (2s^2^, ^1^S) + CLi_4_ (^1^A_1_) as interacting fragments. The eigenvalues ν indicate the size of the charge flow. The direction of charge flow is red → blue. The isovalue for ∆ρ_(1)-(4)_ is 0.0008 au.

|  | Deformation density | Orbital | | | |
| --- | --- | --- | --- | --- | --- |
|  |  | SrCLi_4_ | Sr |  | CLi_4_ |
| ∆ρ_(1)_ | ∆E_orb(1)_ = -33.4 kcal/mol  \|ν_1_\| = 0.93 | HOMO | HOMO s  ν = -0.76 | **→** | LUMO  ν = 0.70 |
| ∆ρ_(2)_ | ∆E_orb(2)_ = -15.4 kcal/mol  \|ν_2_\| = 0.514 | HOMO-1 | LUMO d_σ_  ν = 0.051 | **←** | ­  HOMO  ν = -0.141  +    LUMO  ν = 0.06  +    HOMO-2  ν = -0.01 |
| ∆ρ_(3)_ | ∆E_orb(3)_ = -12.8 kcal/mol  \|ν_3_\| = 0.41 | HOMO-2 | LUMO′ d_π_  ν = 0.09 | **←** | HOMO-1  ν = -0.18 |
| ∆ρ_(4)_ | ∆E_orb(4)_ = -12.8 kcal/mol  \|ν_4_\| = 0.41 | HOMO-2′ | LUMO′′ d_π′_  ν = 0.08 | **←** | HOMO-1′  ν = -0.18 |

**Figure S4.** Plot of the deformation densities, ∆ρ_(1)-(4)_ shown as the sum of α and β electronic charge corresponding to ∆E_orb(1)-(4)_ and the related interacting orbitals in the singlet states of Sr CLi_4_ at the BP86-D3(BJ)/TZ2P-ZORA//BP86-D3(BJ)/def2-QZVPP level using Sr (5s^2^, ^1^S) + CLi_4_ (^1^A_1_) as interacting fragments. The eigenvalues ν indicate the size of the charge flow. The direction of charge flow is red → blue. The isovalue for ∆ρ_(1)_ is 0.001 au for ∆ρ_(2)-(4)_ are 0.0005 au.

|  | Deformation density | Orbital | | | |
| --- | --- | --- | --- | --- | --- |
|  |  | BaCLi_4_ | Ba |  | CLi_4_ |
| ∆ρ_(1)_ | ∆E_orb(1)_ = -34.9 kcal/mol  \|ν_1_\| = 1.02 | HOMO | HOMO s  ν = -0.99 | **→** | LUMO  ν = 0.89 |
| ∆ρ_(2)_ | ∆E_orb(2)_ = -21.2 kcal/mol  \|ν_2_\| = 0.66 | HOMO-1 | LUMO d_σ_  ν = 0.12 | **←** | HOMO  ν = -0.19  +    LUMO  ν = 0.1  +    HOMO-2  ν = -0.02 |
| ∆ρ_(3)_ | ∆E_orb(3)_ = -14.9 kcal/mol  \|ν_3_\| = 0.47 | HOMO-2 | LUMO′ d_π_  ν = 0.15 | **←** | HOMO-1  ν = -0.26 |
| ∆ρ_(4)_ | ∆E_orb(4)_ = -14.9 kcal/mol  \|ν_4_\| = 0.47 | HOMO-2′ | LUMO′′ d_π′_  ν = 0.14 | **←** | HOMO-1′  ν = -0.26 |

**Figure S5.** Plot of the deformation densities, ∆ρ_(1)-(4)_ shown as the sum of α and β electronic charge corresponding to ∆E_orb(1)-(4)_ and the related interacting orbitals in the singlet states of BaCLi_4_ at the BP86-D3(BJ)/TZ2P-ZORA//BP86-D3(BJ)/def2-QZVPP level using Ba (6s^2^, ^1^S) + CLi_4_ (^1^A_1_) as interacting fragments. The eigenvalues ν indicate the size of the charge flow. The direction of charge flow is red → blue. The isovalue for ∆ρ_(1)_ is 0.001 au for ∆ρ_(2)-(4)_ are 0.0006 au.

**Table S3.** Coordinates of singlet AeCLi_4_ calculated at BP86-D3(BJ)/def2-QZVPP.

BeCLi_4_  C_3v_ E=-82.8970740 a.u.

C 0.000000000 0.000000000 0.124553000

Li 0.000000000 1.959524000 -0.118975000

Li -1.696997000 -0.979762000 -0.118975000

Li 1.696997000 -0.979762000 -0.118975000

Li 0.000000000 0.000000000 2.084480000

Be 0.000000000 0.000000000 -1.482497000

BeCLi_4_ C_4v_ E=-82.8969182 a.u.

C 0.000000000 0.000000000 0.329472000

Li 0.000000000 1.982138000 0.256838000

Li -1.982138000 0.000000000 0.256838000

Li 1.982138000 0.000000000 0.256838000

Li 0.000000000 -1.982138000 0.256838000

Be 0.000000000 0.000000000 -1.264723000

MgCLi_4_ C_3v_ E=-268.2611468 a.u.

C 0.000000000 0.000000000 -0.647589000

Li -0.000000000 1.955182000 -0.617745000

Li 1.693237000 -0.977591000 -0.617745000

Li -1.693237000 -0.977591000 -0.617745000

Li 0.000000000 0.000000000 -2.640693000

Mg 0.000000000 0.000000000 1.447277000

MgCLi_4_ C_4v_ E=-268.2602595 a.u.

C 0.000000000 0.000000000 -0.725317000

Li 0.000000000 1.961702000 -0.966885000

Li 1.961702000 0.000000000 -0.966885000

Li -1.961702000 0.000000000 -0.966885000

Li 0.000000000 -1.961702000 -0.966885000

Mg 0.000000000 0.000000000 1.329544000

CaCLi_4_ C_4v_ E=-745.8350945 a.u.

C 0.000000000 0.000000000 -1.056065000

Li -0.000000000 1.946882000 -1.414367000

Li 1.946882000 -0.000000000 -1.414367000

Li -1.946882000 0.000000000 -1.414367000

Li -0.000000000 -1.946882000 -1.414367000

Ca 0.000000000 0.000000000 1.165439000

SrCLi_4_ C_4v_ E=-98.9186021 a.u.

C 0.000000000 0.000000000 -1.530163000

Li 0.000000000 1.941098000 -1.926220000

Li 1.941098000 -0.000000000 -1.926220000

Li -1.941098000 0.000000000 -1.926220000

Li -0.000000000 -1.941098000 -1.926220000

Sr 0.000000000 0.000000000 0.849885000

BaCLi_4_ C_4v_ E=-93.7192409 a.u.

C 0.000000000 0.000000000 -1.807682000

Li -0.000000000 1.936501000 -2.240989000

Li 1.936501000 -0.000000000 -2.240989000

Li -1.936501000 0.000000000 -2.240989000

Li -0.000000000 -1.936501000 -2.240989000

Ba 0.000000000 0.000000000 0.673892000

**Table S4.** Coordinates of singlet AeCLi_4_ calculated at CCSD(T)/def2-QZVPP.

BeCLi_4_ C_3v_ E=-82.5661624 a.u.

C -0.121275000 -0.000435000 0.000435000

Li 0.111857000 -1.964106000 0.257617000

Li 0.116701000 1.206931000 1.569990000

Li 0.118909000 0.759670000 -1.828260000

Li -2.099306000 0.002547000 -0.001611000

Be 1.495791000 -0.003129000 0.001045000

MgCLi_4_ C_3v_ E=-267.5383524 a.u.

C 0.650702000 -0.001399000 0.000659000

Li 0.617598000 -1.962899000 0.275660000

Li 0.622211000 0.745164000 -1.833661000

Li 0.621117000 1.228144000 1.553146000

Li 2.662875000 -0.005694000 0.002496000

Mg -1.456301000 -0.000480000 0.000260000

CaCLi_4_ C_4v_ E=-744.6871306 a.u.

C -1.117116000 0.000489000 -0.000245000

Li -1.490715000 0.063173000 -1.963760000

Li -1.487649000 -1.965058000 -0.063797000

Li -1.487307000 1.965506000 0.063480000

Li -1.491542000 -0.064198000 1.963911000

Ca 1.228717000 -0.000060000 0.000098000

SrCLi_4_ C_4v_ E=-98.2694715 a.u.

C 0.000000000 -0.000000000 -1.608289000

Li 0.000000000 1.960132000 -2.017887000

Li 1.960132000 -0.000000000 -2.017887000

Li -1.960132000 0.000000000 -2.017887000

Li -0.000000000 -1.960132000 -2.017887000

Sr -0.000000000 0.000000000 0.891168000

BaCLi_4_ C_4v_ E=-93.0219180 a.u.

C 0.000000000 -0.000000000 1.944827000

Li 0.000000000 -1.958649000 2.375669000

Li 1.958649000 0.000000000 2.375669000

Li -1.958649000 -0.000000000 2.375669000

Li -0.000000000 1.958649000 2.375669000

Ba -0.000000000 0.000000000 -0.717446000

**Table S5.** Coordinates of the transition state from C_3v_ to C₄_v_ for singlet BeCLi₄ and MgCLi₄ calculated at the BP86-D3(BJ)/def2-QZVPP level.

TS (BeCLi₄ C_3v_ to C₄_v_)

C 0.00025100 0.14940200 -0.13232200

Li 1.98267800 0.06251900 0.13956700

Li -0.00194300 -1.45450100 -1.27118300

Li 0.00300600 2.08914000 -0.28567600

Li -1.98252300 0.06784800 0.13935300

Be -0.00129000 -0.79785800 1.15693800

TS (MgCLi₄ C_3v_ to C₄_v_)

C 0.00025100 0.14940200 -0.13232200

Li 1.98267800 0.06251900 0.13956700

Li -0.00194300 -1.45450100 -1.27118300

Li 0.00300600 2.08914000 -0.28567600

Li -1.98252300 0.06784800 0.13935300

Mg -0.00180066 -1.11176222 1.58417461

**Table S6**. Coordinates of singlet AeCLi4 calculated at HF/def2-QZVPP.

BeCLi_4_ C_3v_ E= -82.2341944 a.u.

C 0.000000000 0.000000000 0.150838000

Li 0.000000000 1.978603000 -0.155412000

Li -1.713521000 -0.989302000 -0.155412000

Li 1.713521000 -0.989302000 -0.155412000

Li 0.000000000 0.000000000 2.094987000

Be 0.000000000 0.000000000 -1.447821000

BeCLi_4_ C_4v_ E= -82.2339083 a.u.

C 0.000000000 0.000000000 0.304243000

Li 0.000000000 1.995945000 0.273146000

Li -1.995945000 0.000000000 0.273146000

Li 1.995945000 0.000000000 0.273146000

Li 0.000000000 -1.995945000 0.273146000

Be 0.000000000 0.000000000 -1.275802000

MgCLi_4_ C_3v_ E= -267.2107953 a.u.

C 0.000000000 0.000000000 -0.661084000

Li 0.000000000 1.965462000 -0.594725000

Li 1.702140000 -0.982731000 -0.594725000

Li -1.702140000 -0.982731000 -0.594725000

Li 0.000000000 0.000000000 -2.655293000

Mg 0.000000000 0.000000000 1.440409000

MgCLi_4_ C_4v_ E= -267.2108148 a.u.

C 0.000000000 0.000000000 -0.710785000

Li 0.000000000 1.966884000 -0.982260000

Li 1.966884000 0.000000000 -0.982260000

Li -1.966884000 0.000000000 -0.982260000

Li 0.000000000 -1.966884000 -0.982260000

Mg 0.000000000 0.000000000 1.337652000

CaCLi_4_ C_4v_ E= -744.3643448 a.u.

C 0.000000000 0.000000000 -1.099257000

Li 0.000000000 1.943951000 -1.499637000

Li 1.943951000 0.000000000 -1.499637000

Li -1.943951000 0.000000000 -1.499637000

Li 0.000000000 -1.943951000 -1.499637000

Ca 0.000000000 0.000000000 1.229559000

SrCLi_4_ C_4v_ E= -97.9490597 a.u.

C 0.000000000 0.000000000 -1.581040000

Li 0.000000000 1.938065000 -2.019477000

Li 1.938065000 0.000000000 -2.019477000

Li -1.938065000 0.000000000 -2.019477000

Li 0.000000000 -1.938065000 -2.019477000

Sr 0.000000000 0.000000000 0.887368000

BaCLi_4_ C_4v_ E= -92.7064207 a.u.

C 0.000000000 0.000000000 -1.858728000

Li 0.000000000 1.933050000 -2.340764000

Li 1.933050000 0.000000000 -2.340764000

Li -1.933050000 0.000000000 -2.340764000

Li 0.000000000 -1.933050000 -2.340764000

Ba 0.000000000 0.000000000 0.700742000
